# Switchable whiteness in liquid crystal networks via solvent-induced microphase segregation

**DOI:** 10.1038/s41467-026-77442-y

**Published:** 2026-09-08

**Authors:** Roshan Nasare, Hilma Panula, Chen Liang, Tero-Petri Ruoko, Hang Zhang, Hongshuang Guo, Arri Priimagi

**Affiliations:** 1 Faculty of Engineering and Natural Sciences, Tampere University, Tampere, Finland; 2 Department of Applied Physics, Aalto University, Espoo, Finland; 3 Present Address: Department of Biomedical Engineering, College of Engineering, Eastern Institute of Technology, Ningbo, China

**Keywords:** Polymers, Liquid crystals, Halogen bonding, Self-assembly

## Abstract

Dynamic control over optical whiteness is promising for applications ranging from energy-efficient coatings to adaptive displays. However, current approaches rely on macroscopic structural transformations or externally imposed refractive-index contrasts, offering limited tunability and scalability. Here, we introduce a liquid crystal network (LCN) that undergoes reversible whitening through solvent-triggered disruption of supramolecular interactions. Exposure to polar protic solvents breaks hydrogen bonds and drives nanoscale phase segregation, generating domains that efficiently scatter visible light. This whitening mechanism enables rapid, robust, and fatigue-resistant switching, with scattering efficiencies exceeding 90% in 100 μm LCN films. By tailoring the molecular design, incorporating halogen-bonding dopants, and post-treatment with acid or base, the optical contrast and switching kinetics can be tuned. We demonstrate potential applications in biometric pattern recognition, underwater visibility enhancement, and solvent discrimination, establishing a materials platform towards dynamic scattering materials.

## Abstract (web summary)

Dynamic control over optical whiteness is promising for applications ranging from energy-efficient coatings to adaptive displays but current approaches offer limited tunability and scalability. Here the authors introduce a liquid crystal network that undergoes reversible whitening through solvent-triggered disruption of supramolecular interactions.

## Introduction

White materials are essential in both natural systems^1,2^ and technological applications^3,4^, playing crucial roles in thermal regulation^5–7^, optical camouflage^8,9^, and smart displays^10,11^. In synthetic materials, whiteness is typically achieved by embedding high-refractive-index inclusions, such as TiO_2_^12^ or ZnO^13^, within polymer matrices to scatter light across the visible spectrum. While effective, these materials have fixed optical properties after fabrication, yielding a bright white, yet static, appearance^14^. In contrast, dynamically tunable whiteness, i.e., reversible switching between transparent and white states, holds promise for adaptive coatings, energy-efficient building materials, and responsive displays^15,16^. However, achieving such dynamic tunability is challenging, as most existing strategies depend on irreversible structural changes or complex control mechanisms, limiting their practical functionality.

Hydrogels exploit water absorption to induce swelling and alter the refractive-index contrast between the polymer and surrounding solvent. While this enables reversible whitening^17–21^, it typically involves substantial volumetric expansion and suffers from mechanical fatigue or degradation under repeated cycling^22,23^. Hydrogels based on poly(*N*-isopropylacrylamide) (PNIPAm) can also undergo phase-transition-induced whitening^5,15,24,25^, but these transitions generally require external stimuli, such as temperature changes. In addition, the low elastic modulus and mechanical softness of conventional hydrogels make them prone to creep, fracture, and collapse under cyclic operation^26,27^. Similarly, liquid-infused elastomers, relying on deformation-induced air pockets for light scattering^28^, and polymer-dispersed liquid crystals, commonly used in smartwindows^29–31^, often face issues with mechanical instability and scalability, limiting their long-term applicability. Thus, there remains a need for materials that achieve dynamic whitening through an intrinsic nanoscale mechanism that eliminates large-scale swelling, air-pocket formation, and complex refractive-index tuning, while maintaining mechanical robustness and flexibility.

Here, we present a supramolecularly functionalized liquid crystal network (LCN) that overcomes these limitations by utilizing a different whitening mechanism: solvent-induced phase segregation. Unlike systems that rely on bulk expansion or external triggers, our material undergoes spontaneous microphase segregation upon water absorption, forming nanoscale scattering domains within the polymer matrix. By precisely balancing hydrogen and halogen bonds within the LCN, we control phase separation dynamics and modulate optical contrast. Hydrophobic dopants engaging in halogen bonding further enhance whitening efficiency, while acid–base treatments enable fine-tuning of switching speed and contrast. 100 µm-thick films exhibit scattering efficiencies up to 90 % under wet conditions and retain stable optical performance over repeated cycles, underscoring their robustness. Leveraging these features, we showcase potential applications in biometric pattern recognition, underwater visibility enhancement, and solvent discrimination. This scalable, durable, and mechanically resilient platform offers a new route to dynamic white materials for smart coatings and adaptive optical technologies.

## Results

**Fig. 1:**
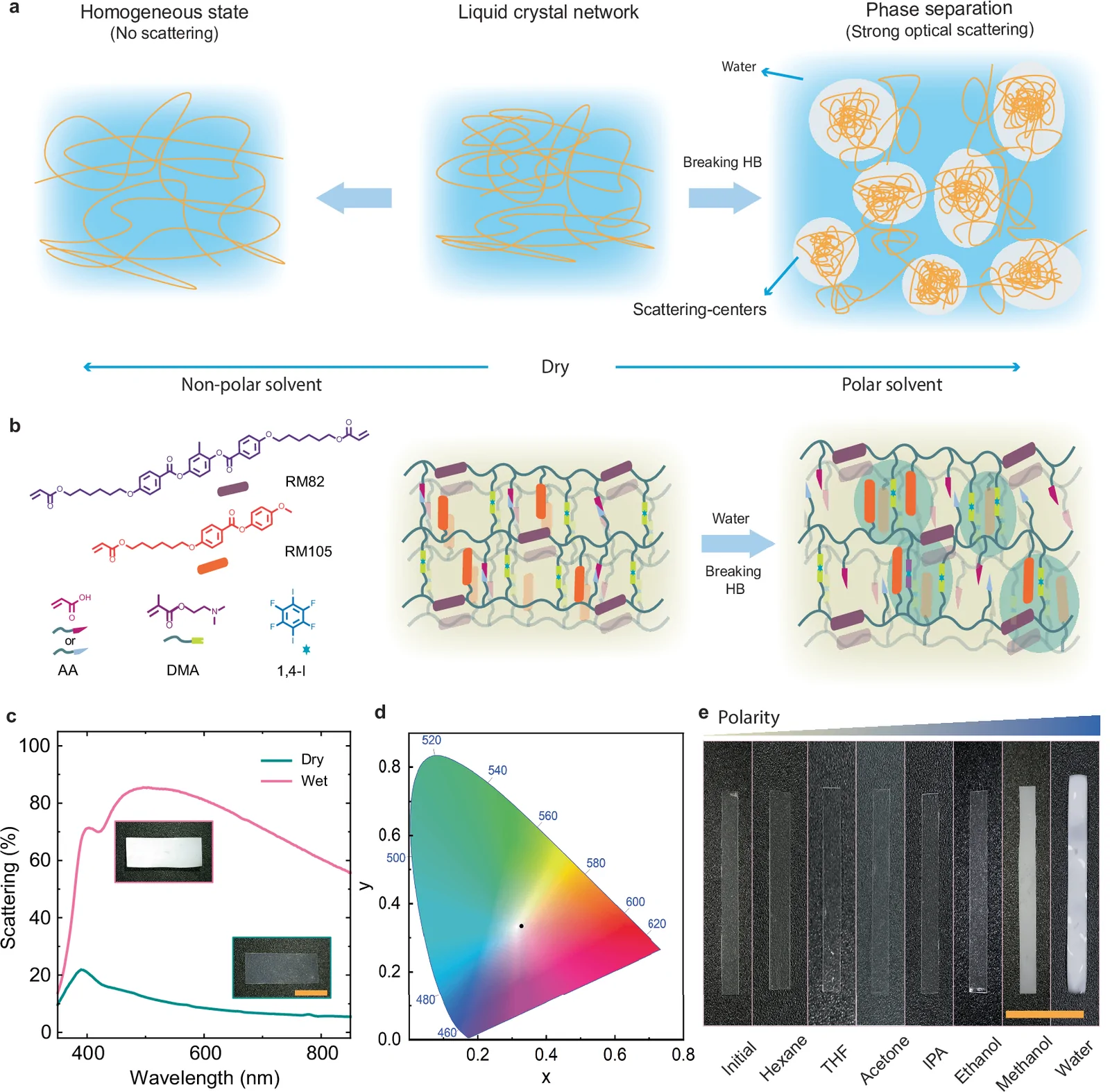
Dynamic whitening in LCNs. **a** Schematic illustration of the reversible structural transition in an LCN film into a phase-separated scattering state upon exposure to polar protic solvents. In non-polar solvents, the film remains homogeneous and transparent. **b** Chemical structures of LCN components (RM82, RM105, AA, DMA, and 1,4-I), and schematic representation of HB disruption and scattering center formation upon hydration. **c** Total scattering spectra of 100 µm LCN_AA+DMA+I_ film in the dry and hydrated states. Insets: photographs of the transparent (dry) and white (wet) films. **d** CIEXYZ chromaticity diagram of the hydrated LCN_AA+DMA+I_ film confirming the white appearance. **e** Photographs of 100 µm LCN_AA+DMA+I_ film immersed in solvents of increasing polarity, showing a transition from transparent to white state in methanol and water. Scale bar: 1 cm.

Our design premise is that dynamic whitening in LCNs can be achieved by integrating hydrophilic and hydrophobic domains within a single supramolecularly functionalized framework. Hydrogen bonding (HB), a quintessential hydrophilic interaction widely used to sensitize LCNs to humidity^32–35^, is readily disrupted by polar solvents. We hypothesize that when coupled to hydrophobic motifs, such disruption can trigger nanoscale phase segregation within the network. Halogen bonding (XB) represents such hydrophobic supramolecular interaction^36^. As schematized in Fig. 1a, we expect that exposure to polar protic solvents, such as water, disrupts HB and solvates hydrophilic segments, thereby driving aggregation of hydrophobic domains and forming phase-separated scattering centers. The resulting refractive-index mismatch would yield broadband light scattering and dynamic whitening in polar solvents, whereas nonpolar solvents leave the network intact and optically transparent.

The above hypothesis was tested with polymer networks composed of the compounds shown in Fig. 1b and Supplementary Fig. 1. The LCN matrix consists of liquid crystalline mesogens RM82 and RM105, which form the anisotropic backbone of the network. Acrylic acid (AA) was used as the hydrophilic co-monomer. Its carboxylic acid groups form water-sensitive HB dimers that can be disrupted via alkaline treatment, yielding hygroscopic carboxylate salt^37,38^. Both the pristine and base-treated states are water-sensitive and provide distinct functionalities to the LCN^39–41^. Dimethylaminoethyl methacrylate (DMA) was used as a more hydrophobic co-monomer that serves as an XB acceptor^42,43^ and also as an acid-sensitive component to make the film hygroscopic by acid treatment^39^. As the XB donor interacting with the DMA units, 1,4-diiodotetrafluorobenzene (1,4-I) was introduced into the LCN. A typical film composition, denoted as LCN_AA+DMA+I_, contains ca. 10 mol% LC mesogens (RM82:RM105 in a 1:5 ratio), 60 mol% AA, and 30 mol% DMA. As each 1,4-I molecule has two XB-donating iodine atoms, the DMA:1,4-I ratio was fixed to 2:1. The resulting polymer films (thickness range of 5–100 µm) are optically transparent and macroscopically isotropic yet exhibit birefringence under moderate mechanical stretching (10%), confirming the liquid crystalline nature of the network (Supplementary Fig. 2). Further details of the film preparation are provided in the Methods section.

As shown in Fig. 1c, the initially transparent LCN film indeed turns white upon immersion in water. This transition is fully reversible, and the film regains transparency after drying on a hot plate (Supplementary Movie 1 and Supplementary Fig. 3). As the films are relatively thin, they exhibit both backward (reflective) and forward (transmissive) scattering. Therefore, total light scattering was quantified as the sum of both components, as detailed in Supplementary Fig. 4 and the Methods section. The scattering spectra (Fig. 1c) reveal a significant enhancement in visible light scattering (400–800 nm) upon hydration, with total scattering efficiency exceeding 80% in 100 µm films. This value is comparable to that of conventional white materials^14,44–46^, yet achieved without incorporating rigid inorganic fillers. The corresponding CIE XYZ chromaticity coordinates confirm the visual perception of white in the hydrated state, with a correlated color temperature (CCT) of 5681 K (Fig. 1d). Consistent with the underlying hypothesis, the dynamic whiteness of LCN_AA+DMA+I_ film is solvent-polarity-dependent, remaining transparent in low-polarity solvents, such as hexane and toluene, but becoming opaque in methanol and water (Fig. 1e).

**Fig. 2:**
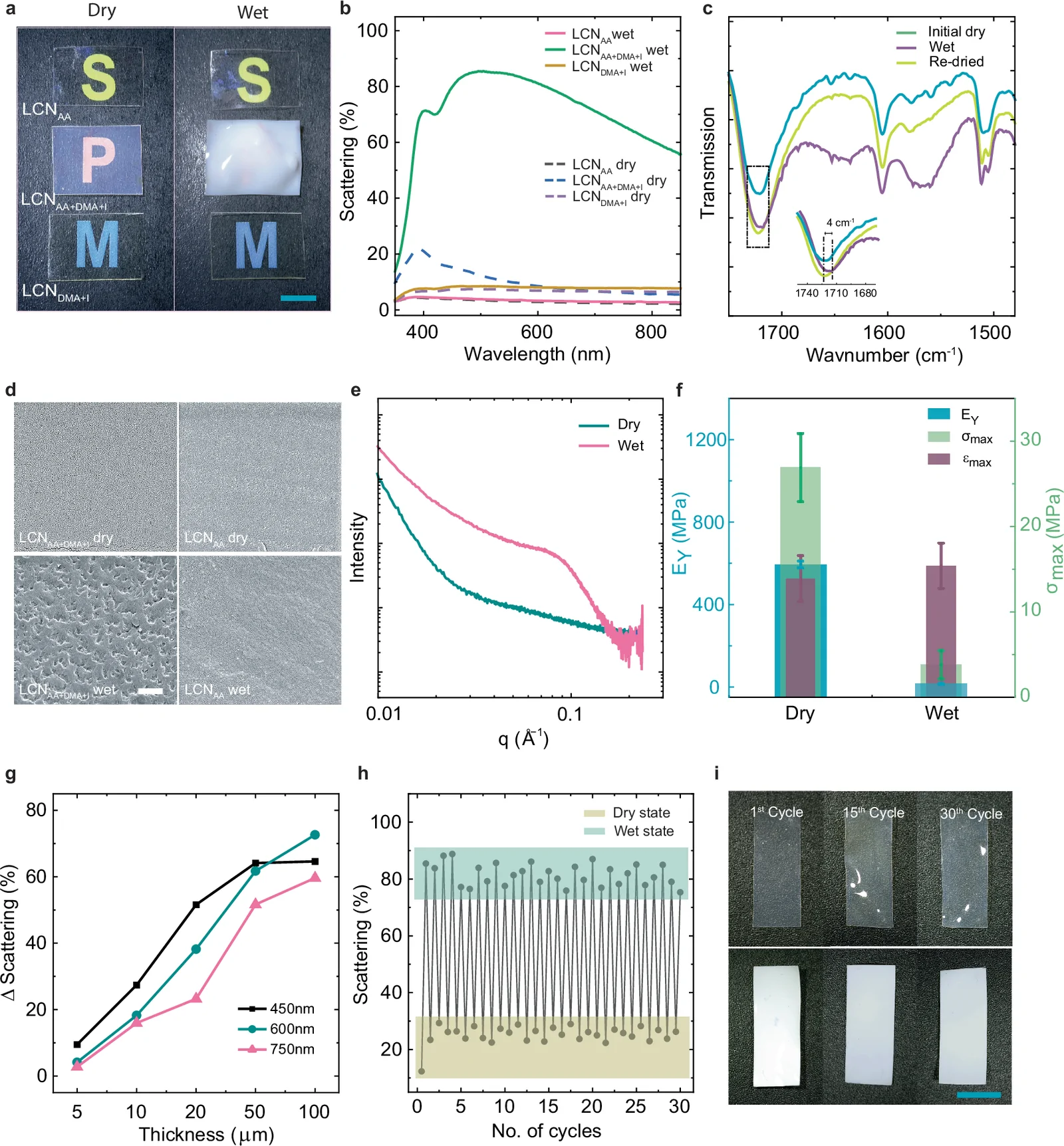
Solvent-induced phase segregation in LCN_AA+DMA+I_, LCN_AA_, and LCN_DMA+I_. **a** Photographs of LCN films (LCN_AA_, LCN_AA+DMA+I_, and LCN_DMA+I_) in dry and wet states. **b** Total scattering spectra of respective LCNs in dry and wet states. **c** FTIR spectra of LCN_AA+DMA+I_ in the initial dry, wet, and re-dried states. **d** SEM images of LCN_AA+DMA+I_ and LCN_AA_ under dry and wet states. Scale bar: 800 nm. **e** Small-angle X-ray scattering (SAXS) profiles of LCN_AA+DMA+I_ film in dry and wet states. **f** Mechanical properties (Young’s modulus, tensile strength, and fracture strain) of dry and wet LCN_AA+DMA+I_ film. Error bars represent the SD obtained from *n* = 3 measurements. **g** Thickness-dependent light scattering change of LCN_AA+DMA+I_ film at different wavelengths. **h** Reversible switching of total scattering of LCN_AA+DMA+I_ film at 500 nm over 30 dry-wet cycles, demonstrating reversibility. **i** Photographs of the same film over the dry-wet cycles. Scale bar: 1 cm. Film thickness: 100 µm.

To further elaborate on the material requirements governing the switchable whitening, we varied the hydrophilic–hydrophobic balance of the polymer network by removing either AA or DMA, while keeping the mesogen content and the DMA:1,4-I ratio constant (Supplementary Table 1). As shown in Fig. 2a, b, the control films composed solely of AA (LCN_AA_) or DMA and 1,4-I (LCN_DMA+I_) remained optically transparent in both dry and wet states, indicating that neither hydrophilic nor hydrophobic character alone is sufficient to induce dynamic whitening. In contrast, LCN_AA+DMA+I_, containing both AA and DMA, exhibited pronounced water-induced whitening, confirming that a delicate balance between hydrophilic and hydrophobic moieties is essential for the formation of the water-triggered scattering domains. Fourier transform infrared (FTIR) spectra (Fig. 2c) confirmed the disruption of HB dimers, leading to a shift in the carbonyl (C = O) stretching band from 1722 cm^−1^ to 1718 cm^−1^ upon water exposure and reverting to the original position after drying^47^. Scanning Electron Microscopy (SEM) analysis (Fig. 2d) revealed distinct nanoscale phase separation in the water-treated LCN_AA+DMA+I_ sample, while no phase separation was observed in wet LCN_AA_.

Structural characterization by small-angle X-ray scattering (SAXS) further confirmed these findings (Fig. 2e). In the dry state, the LCN_AA+DMA+I_ film exhibits low scattering intensity across the measured *q*-range, indicating a relatively homogeneous morphology with minimal contrast between domains. Upon swelling in water, a pronounced increase in scattering intensity in the low-*q* region occurs, characteristic of nanoscale phase separation and domain formation^48,49^. In contrast, the SAXS profile of LCN_AA_ confirms the absence of such phase separation even in the wet state (Supplementary Fig. 5c). Mechanical testing revealed that the dry LCN_AA+DMA+I_ film (100 µm) has a Young’s modulus, tensile strength, and fracture strain of 594 MPa, 27 MPa, and 28%, respectively. Upon hydration, the modulus and breaking stress sharply decrease to 12 MPa and 4 MPa, respectively, presumably due to disruption of the carboxylic dimers acting as supramolecular crosslinks, while the fracture strain remains nearly constant at 31% (Fig. 2f, Supplementary Fig. 6, and Supplementary Table 2). We further evaluated the fracture toughness of LCN_AA+DMA+I_ by introducing a 0.5 mm notch into the 5 mm wide LCN film and subsequently performing tensile testing (Supplementary Fig. 7). The presence of the notch reduced the breaking stress and strain in the dry state from 27 MPa to 21 MPa and from 28% to 4%, respectively. The corresponding fracture energy was calculated to be 36 kJ m^−2^. In the wet state, the breaking stress decreased from 4 MPa to 0.7 MPa and the breaking strain from 31% to 11%. The corresponding fracture energy was 3.42 kJ m^−2^. The reduction in mechanical performance can be attributed to the notch acting as a stress concentrator, promoting crack propagation and lowering the material’s resistance to deformation and fracture. Nevertheless, the notched film retained the reversible whitening behavior and remained functional under repeated use.

Thickness-dependent optical scattering data (Fig. 2g) reveal that the scattering efficiency, and thus the perceived whiteness of LCN_AA+DMA+I_, increase with film thickness across the visible wavelength range (full spectra are given in Supplementary Fig. 8). The characteristic attenuation length of wet LCN_AA+DMA+I_ was determined by measuring the total transmittance (*T*_total_) of samples with different thicknesses and plotting ln(*T*_total_) as a function of sample thickness (Supplementary Fig. 9). The data exhibited a linear dependence, indicating that optical attenuation scales consistently with thickness and that the scattering morphology across samples with different thicknesses is comparable. From the slope of the linear fit, the characteristic attenuation length (*l*_s_) was calculated to be 116 μm, corresponding to an attenuation coefficient (1/*l*_s_) of 86.2 cm^−1^ at 800 nm. This value is substantially larger than attenuation coefficients reported for transparent wood (*ca*. 1.5 cm^−1^) and polymer aerogels (*ca*. 4 cm^−1^)^50,51^, indicating strong attenuation of transmitted light in the present material. The kinetics of the transparent-to-white transition were monitored via transmittance measurements. The transition speed increased with both sample thickness and solvent temperature (Supplementary Figs. 10–13). The former is possibly due to more rapid formation of scattering domains within the bulk, and the latter can be rationalized through faster nanoscale rearrangements and the dynamics of HB and XB interactions, which govern the phase separation process. The durability of LCN_AA+DMA+I_ film was tested over repeated wetting and drying cycles, and the film showed reversible whiteness switching with no fatigue over 30 switching cycles (Fig. 2h, i).

**Fig. 3:**
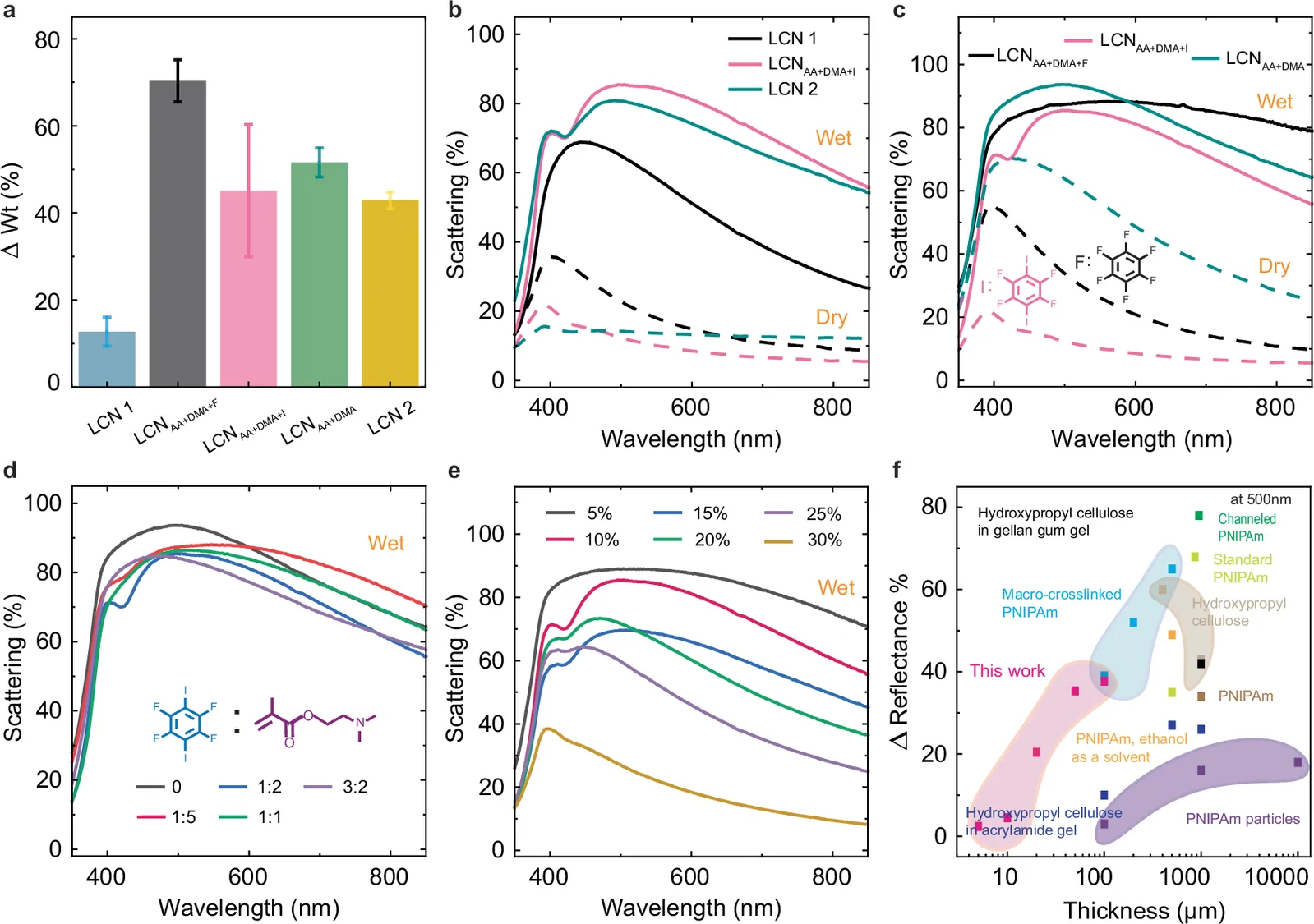
Water-induced optical scattering behavior of different LCN compositions. **a** Water uptake measurement (Δwt%) of the studied LCN films in the wet state. Error bars represent the SD obtained from *n* = 3 measurements. **b** Total scattering spectra of LCN 1, LCN_AA+DMA+I_, and LCN 2 in wet (solid) and dry (dashed line) states. **c** Total scattering spectra of LCN_AA+DMA+F_, LCN_AA+DMA+I_, and LCN_AA+DMA_ in dry and wet states. **d** Wet-state total scattering spectra upon changing the 1,4-I:DMA ratio. **e** Wet-state total scattering spectra upon changing the mesogen content in the LCN. **f** Change in reflectance comparison of LCN_AA+DMA+I_ films with reported system of same category at 500 nm in white and transparent state with known system relative to thickness. All sample thicknesses: 100 µm.

The dynamic whiteness depends on the relative fraction between AA and DMA, as illustrated in Figs. 3a and 3b. Decreasing the AA molar ratio from 59.5 mol% to 47.6 mol% (LCN 1) led to loss of switching contrast, manifested by both increased dry-state scattering and decreased wet-state scattering. In contrast, increasing the molar fraction to 71.4 mol% (LCN 2) maintained high whiteness-switching contrast, albeit slightly lower than for LCN_AA+DMA+I_ (Fig. 3b; for individual spectra and pictures, see Supplementary Fig. 14). This indicates that a clear excess of AA relative to DMA is needed for efficient switching. As the original stoichiometry (LCN_AA+DMA+I_) produced the best switching contrast, further experiments were conducted using that composition. Unlike LCN_AA_and LCN_DMA+I_, the films exhibiting dynamic whitening showed clear water-induced swelling (Fig. 3a).

On our path to identifying the material design parameters governing dynamic whitening in LCNs, two key questions remain: (i) What is the specific role of halogen bonding, and (ii) how does the mesogenic backbone influence the process? To address question (i), we replaced 1,4-I with hexafluorobenzene (F), a passive dopant unable to form halogen bonds (LCN_AA+DMA+F_). We also prepared a formulation without any dopant (LCN_AA+DMA_) and compared both to the halogen-bonded LCN_AA+DMA+I_. All three materials swelled upon hydration (Fig. 3a) and exhibited white appearance (Supplementary Fig. 15). However, their most notable difference to LCN_AA+DMA+I_ was observed in the dry state, in which neither LCN_AA+DMA+F_ nor LCN_AA+DMA_ were transparent owing to phase inhomogeneities (Fig. 3c). The SEM and SAXS data of LCN_AA+DMA_ provided additional evidence for the water-induced nanoscale phase separation in the wet state, showing an increase in scattering intensity in the low-*q* region as compared with its dry state (Supplementary Fig. 5a, b).

The content of the XB donor also affected the scattering properties (Supplementary Table 3). In the wet state, all samples exhibited comparable scattering intensities, suggesting that donor concentration has only a minor effect on maximum whiteness (Fig. 3d, full spectra at Supplementary Fig. 16). In contrast, in the dry state, scattering increased markedly in the absence of donors. These findings demonstrate that while donor concentration does not directly control the absolute whitening efficiency, it modulates the switching contrast by suppressing dry-state scattering. Interestingly, replacing the tertiary amine (DMA) with an equivalent amount of LC monomers (RM82 & RM105, LCN CT 5), a hydrophobic co-monomer (butyl methacrylate; LCN CT 6), or a secondary amine (N-isopropylacrylamide; LCN CT 7) eliminates the whitening response entirely. These results indicate that DMA-mediated supramolecular interactions are essential for the observed reversible whitening (Supplementary Table 4 and Supplementary Fig. 17b–d).

To study the role of mesogens, we prepared several control formulations (Supplementary Table 4). A minimal system containing only RM82 (LCN CT1, Supplementary Fig. 17a) retained hydration-induced whitening, whereas formulations lacking RM82 (CT2-CT4) either failed to form stable films or exhibited no switching, highlighting the importance of covalent crosslinking. Next, we investigated the influence of mesogen concentration (RM82 + RM105) on dynamic whitening. A series of LCNs with increasing mesogen content was prepared, while keeping AA, DMA, and 1,4-I content constant (Supplementary Table 5). Varying the mesogen fraction had a minor effect on dry-state scattering but strongly affected the whiteness in the hydrated state. Specifically, higher mesogen fractions (30 mol% and above) suppressed phase separation upon hydration, thus diminishing scattering efficiency and whiteness (Fig. 3e, Supplementary Fig. 18). This indicates that mesogen density plays an important role in governing phase-separation dynamics, potentially by modulating domain stiffness and/or the hydrophobicity of the film.

To further clarify the role of LC mesogens, we performed control experiments in which the LC units were replaced with non-LC monomers: RM82 was replaced with bisphenol A polyethylene glycol diether diacrylate (m + n ≈ 10) or 1,4-phenylene diacrylate, and RM105 with phenyl acrylate (Supplementary Table 6). These polymers, lacking LC units, exhibited no bright–dark contrast when stretched and imaged with a polarized optical microscope (Network 2, Supplementary Fig. 2c, d). They did, however, display a pronounced and reversible whitening response upon exposure to water (Supplementary Fig. 19). This indicates that the reversible optical scattering primarily originates from disruption of supramolecular HB and nanoscale phase segregation. LC mesogens, while influencing mechanical and structural properties, are therefore not essential for the whitening behavior.

A comparison of reflectance with previously reported systems exhibiting switchable whiteness is presented in Fig. 3f. For a 100 µm thick LCN_AA+DMA+I_ film, we observe a reflectance contrast of 40% at 500 nm (see Supplementary Table 7 and diffuse reflectance spectra in Supplementary Fig. 4d). Because reflectance values are most commonly reported in the literature, reflectance was used as the primary metric for comparison. Notably, when total scattering is considered together with transmittance, the contrast between the transparent and white states reaches 75% at 500 nm (Fig. 1c).

**Fig. 4:**
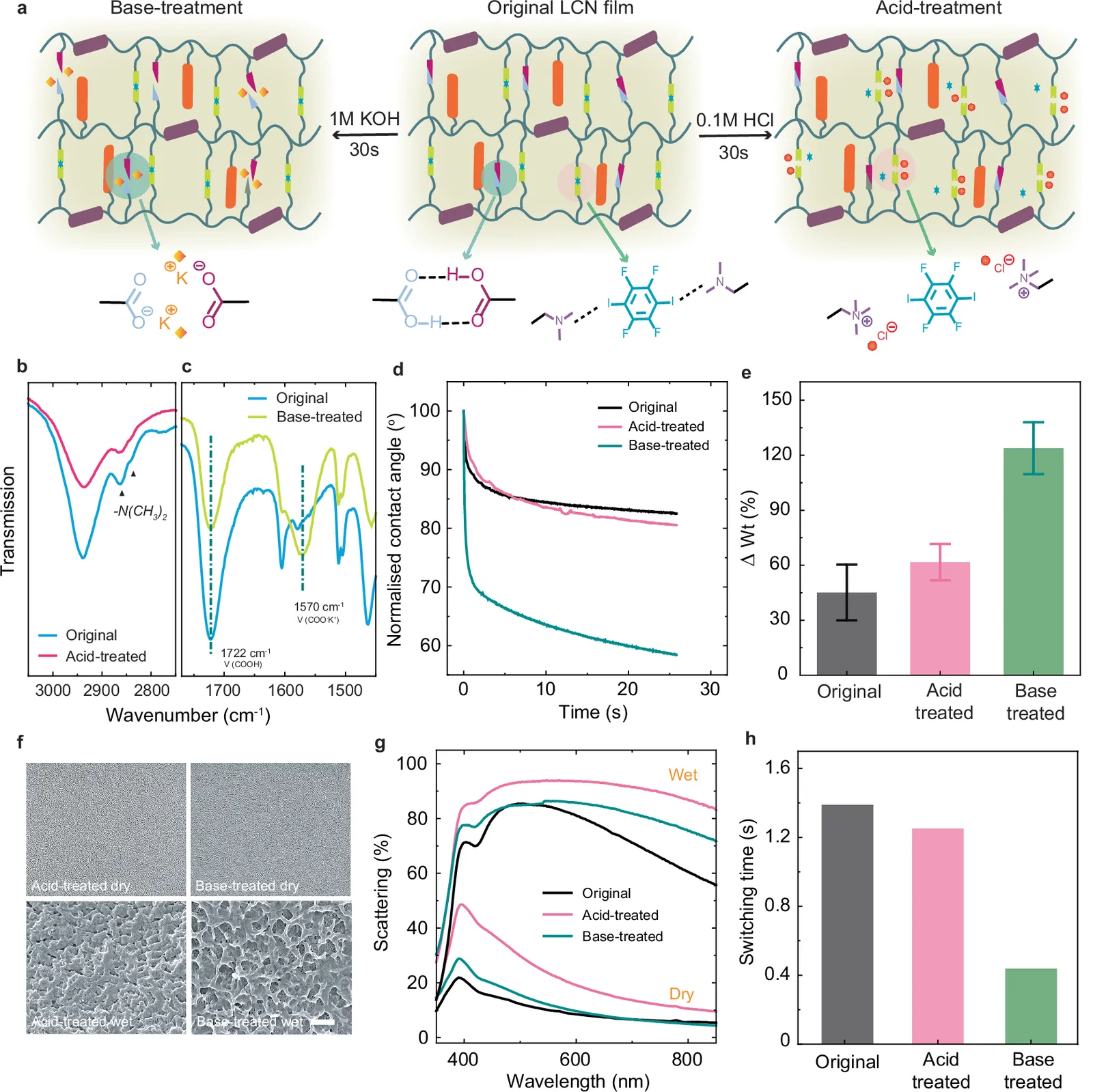
Influence of acid and base treatments on the whitening of LCN_AA+DMA+I_. **a** Schematic representation of the LCN_AA+DMA+I_ and the same film after acid and base treatment, leading to salt formation and making the film more hygroscopic. Comparison of FTIR spectra of original LCN film with **b** acid-treated (0.1 M aq. HCl, 30 s), and **c** base-treated (1 M aq. KOH, 30 s) LCN film. **d** Normalized contact angle of original, acid-treated, and base-treated film. **e** Water uptake measurements (Δwt%) of respective films between wet and dry state, showing significant increases after acid and base treatment. Error bars represent the SD obtained from *n* = 3 measurements. **f** SEM images of acid- and base-treated films under dry and wet conditions, showing phase separation in the wet state. Scale bar: 800 nm **g** Total scattering spectra across 350–850 nm of LCN_AA+DMA+I_ in original, acid-treated, and base-treated films in dry and wet conditions. **h** Corresponding transparent-to-white state kinetics analysis of respective films. Film thickness: 100 µm.

Having established the critical role of molecular composition and supramolecular interactions in water-induced dynamic scattering, we next explored post-synthetic chemical modifications to further enhance material performance. In particular, we investigated whether acid or base treatments could amplify the whitening response by modulating the hydrophilicity of the LCN films. LCN_AA+DMA+I_ films were immersed in dilute HCl (0.1 M) or KOH (1 M) solutions for 30 s to generate their respective salt forms, rinsed, and dried (Fig. 4a). The FTIR spectra (Fig. 4b, c and Supplementary Figs. 20a, 21a) confirmed successful chemical conversion: protonation of the dimethylamino groups eliminated the -N(CH_3_)_2_ stretching bands at 2777 and 2862 cm^−1^ upon acid treatment^39^, while carboxylic salt formation upon base treatment generated a new COO⁻ band at 1570 cm⁻^1^ and diminished the COOH stretching band at 1722 cm⁻^1^^40^. The base treatment significantly enhanced the hydrophilicity of LCN_AA+DMA+I_, as evidenced by contact angle measurements (Fig. 4d, Supplementary Fig. 20b, 21b) and water uptake measurements (Fig. 4e), where base-treated LCN absorb more than twice the amount of water compared to untreated samples.

SEM imaging (Fig. 4f) revealed pronounced water-induced microphase separation in both acid- and base-treated samples, with base-treated samples displaying larger, interconnected porous domains, corresponding to an increase in optical scattering upon swelling (Fig. 4g). Acid-treated films achieved > 90 % broadband scattering efficiency in the wet state. SAXS analysis (Supplementary Figs. 20c, 21c) confirmed phase separation in the hydrated state. Kinetic studies (Fig. 4h and Supplementary Figs. 20d, 21d) revealed accelerated whitening dynamics, especially upon base treatment, indicating enhanced solvent diffusion and network rearrangement. These findings collectively demonstrate that acid–base tuning of LCN chemistry enables enhancement in wet-state scattering and control over its optical response, providing a powerful post-treatment method for optimizing stimuli-responsive whitening.

**Fig. 5:**
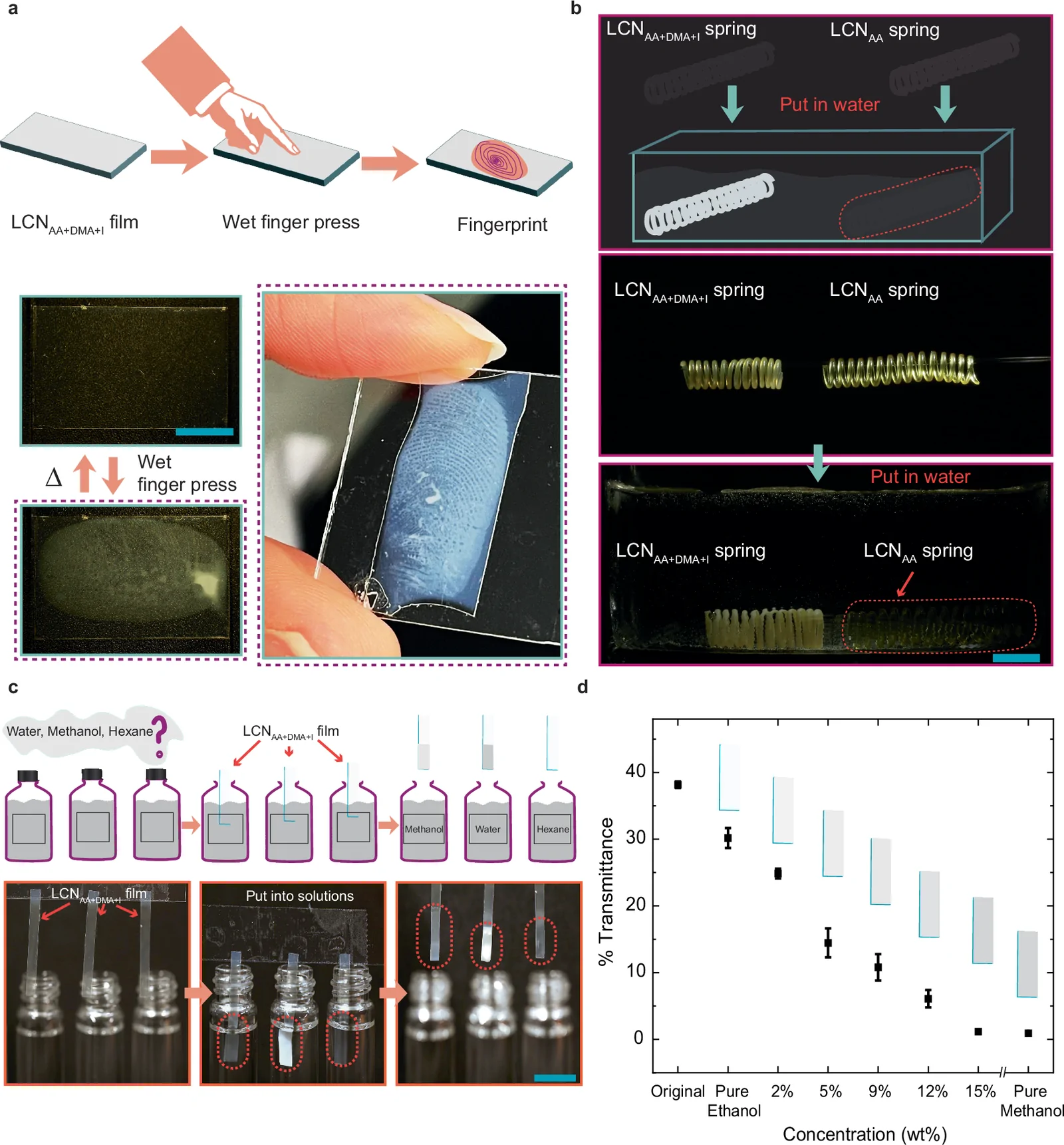
Application demonstrations for the solvent-induced phase segregation. **a** Schematic illustration (top) and photographs of fingerprinting (bottom) on an LCN_AA+DMA+I_ surface. **b** Transparent-to-opaque transition enables rapid underwater visualization. Schematics (top) and photographs (middle and bottom) compare LCN_AA+DMA+I_ and LCN_AA_ springs in water, showing that only LCN_AA+DMA+I_ becomes opaque and readily identifiable. **c** Schematic and photographs illustrating rapid detection of unlabeled solvents using LCN_AA+DMA+I_. Top: Schematic illustration of the immersion-based detection process. Bottom: Photographs before and after immersion demonstrating polarity-induced changes in whitening. **d** Detection of methanol in ethanol using a base-treated LCN_AA+DMA+I film_. Error bars represent the SD obtained from *n* = 3 measurements. All scale bars are 1 cm; film thickness is 100 µm.

To demonstrate the functional versatility of the phase-segregation-induced scattering, we explored the utility of the LCN_AA+DMA+I_ across several application scenarios, like biometric pattern recognition, underwater visibility enhancement, and solvent discrimination. When gently pressed with a wet finger, the film displayed a high-contrast fingerprint pattern, arising from localized hydration. This spatially confined water absorption induces phase separation within the LCN, producing enhanced light scattering at the ridge contact points (Fig. 5a). The response is rapid, reversible, and requires no external stimulus, enabling simple visualization of touch patterns and suggesting potential for transient biometric marking and touch-responsive interfaces. To assess performance in submerged environments, LCN_AA+DMA+I_ was fabricated into a helical spring geometry. Upon immersion, the spring rapidly turned white and became clearly visible, whereas a control LCN_AA_ spring lacking the whiteness switching remained nearly transparent (Fig. 5b). This demonstrates the material’s capacity for real-time visual identification under aquatic conditions.

Exploiting the polarity-dependent scattering response, we developed a simple visual solvent discrimination platform. Identical LCN_AA+DMA+I_ films immersed in solvents of different polarity exhibited distinct scattering intensities within seconds, enabling easy differentiation between polar and non-polar environments (Fig. 5c): strong whitening occurred in water, weaker in methanol, and no optical change was observed in hexane. This solvent-responsiveness, achieved without bulky additives or complex readout systems, highlights the potential of the material as a simple and environmentally benign sensing platform. In addition, we demonstrate relative humidity (RH) dependent optical modulation in a controlled humidity chamber, where film transparency gradually decreases upon increasing RH ( > 60% RH) due to progressive water-vapor uptake and the resulting nanoscale phase segregation. This enables simple, instrument-free humidity sensing based on optical contrast changes (Supplementary Fig. 22). Furthermore, laser- patterned structures were fabricated to further exploit the spatially controlled scattering transition. The patterned films remain optically transparent under dry conditions but reveal hidden information upon water exposure, allowing on-demand visual decoding without external illumination or processing (Supplementary Fig. 23). Finally, base-treated LCN_AA+DMA+I_ films were evaluated for methanol detection in methanol–ethanol mixtures, a challenging task due to the similar polarity of the two solvents. The treated film enabled reliable detection of methanol concentrations as low as 2 wt% through changes in transmittance spectra (Fig. 5d). In contrast, untreated films exhibited noticeable opacity only at methanol concentrations exceeding 15 wt%, demonstrating the effectiveness of post-modification in enhancing sensitivity (Supplementary Fig. 24). Overall, these results highlight that the same phase-segregation-driven scattering mechanism can be readily extended to environmental, chemical, and spatially encoded optical functions. In contrast to many existing whitening materials that rely on heating, bulk swelling, or electrical stimulus, our approach is triggered simply by exposure to water or polar solvents, enabling straightforward implementation and broadened applicability. The simplicity of stimulus input, combined with rapid optical readout and facile patterning capability, provides a versatile platform for low-energy, visually intuitive sensing and information encoding applications.

## Discussion

Based on our observations, we attribute the reversible whitening to solvent-induced disruption of supramolecular interactions, primarily HB, which triggers nanoscale phase segregation within the LCN. Under ambient conditions, hydrogen-bonded carboxylic dimers together with halogen-bonded hydrophobic domains maintain a homogeneous network. Exposure to polar, especially protic, solvents disrupts the hydrogen-bonded dimers, triggering phase segregation and the formation of light-scattering domains. In contrast, non-polar solvents largely preserve the supramolecular network, suppressing domain formation and preventing optical whitening.

The mechanical robustness of these LCN films markedly exceeds that of previously reported hydrogels exhibiting switchable whiteness^44^, with a breaking stress of *ca*. 27 MPa for 100 µm thick LCN films *vs. ca*. 4.2 MPa for 1000 µm thick hydrogels. Remarkably, even films as thin as 10 µm exhibit up to 43% visible-light scattering in the wet state while maintaining excellent reversibility and showing no signs of fatigue over repeated wetting–drying cycles (Supplementary Fig. 25). The reflectance contrast reaches 40% at 500 nm (Fig. 3f) and can be further increased to nearly 50% through simple post-treatment with acid or base. Unlike hydrogel-based dynamic scattering materials that often rely on macroscopic deformation or thermal activation, our LCN provides rapid, robust, and fatigue-resistant optical switching via solvent-triggered nanoscale phase segregation. Furthermore, the scattering efficiency and response kinetics can be systematically tuned through molecular design, incorporation of XB dopants, and post-synthetic modification.

The observed whitening behavior is not limited to the specific mesogenic components employed here. RM82 and RM105 were selected as representative examples providing network stability and flexibility. When replacing RM82 with common mesogenic crosslinkers RM257 or C6BAPE, the dynamic whitening response is preserved (Supplementary Fig. 26). In fact, even non-liquid-crystalline building blocks (Networks 1–4, Supplementary Table 6) can be used, although in some cases at the expense of the mechanical properties of the network. These results indicate that the whitening transition is governed primarily by solvent-induced disruption of the hydrogen-bonded network, leading to nanoscale phase segregation and light scattering, rather than by the specific chemical identity of the mesogens, highlighting the generality of the underlying mechanism and establishing a broadly applicable design principle for dynamic scattering materials.

In summary, we demonstrate that dynamic whitening in liquid crystal networks can be achieved through solvent-induced supramolecular reconfiguration rather than macroscopic deformation. Comprehensive structural, mechanical, and optical analyses establish polar protic solvents as molecular triggers generating nanoscale domains responsible for broadband scattering. Systematic variation of molecular composition enabled control over scattering efficiency and switching kinetics, providing insight into the underlying structure–function relations. The materials combine high durability, excellent reversibility, and thin form factors with whitening performance readily enhanced via post-synthetic modification. This molecular-level design strategy opens new opportunities for adaptive coatings, smart sensing platforms, and reversible optical patterning.

## Methods

All reagents and chemicals were used as received without further purification. 1,4-Bis-[4-(6-acryloyloxyhexyloxy)benzoyloxy]−2-methyl-benzene (RM82), 4-methoxybenzoic acid 4-(6-acryloyloxy-hexyloxy) phenyl ester (RM105), 1,4-Bis[4-(3-acryloyloxypropyloxy)benzoyloxy]−2-methylbenzene (RM257), and 4-(6-(Acryloyloxy)hexyloxy)phenyl 4-(6-(acryloyloxy)hexyloxy)benzoate (C6BAPE) were purchased from Synthon Chemicals GmbH & Co. Acrylic acid (AA), 1,4-phenylene diacrylate, Phenyl acrylate, N-isopropylacrylamide, butyl methacrylate and photo initiator (I-651) were purchased from Sigma-Aldrich. 2-(Dimethylamino)ethyl Methacrylate (DMA), Hexafluorobenzene (F), and Bisphenol A Polyethylene Glycol Diether Diacrylate were purchased from TCI, and 1,4-Diiodotetrafluorobenzene (1,4-I) from Flurochem in its white crystalline form.

### Fabrication of LCN films

Two clean glass slides (2.5 × 3.0 × 0.2 cm) were spin-coated with polyvinyl alcohol (PVA, 5 wt% in water, 4000 rpm, 1 min) and heated at 100 °C for 10 min. The coated glass substrates were glued together using UV glue (Norland, NOA65) mixed with microspheres (Thermo Scientific, 5–100 µm) to obtain the desired sample thickness. The LC mixtures were prepared by mixing the components in the required fractions and adding 2 wt% of photoinitiator (IRG-651). The mixture was heated and mixed at 100 °C until all components melted to a clear liquid, before infiltration into a cell via capillary action. The cells were slowly cooled to 60 °C and polymerized under UV light (385 nm, 35 mW cm^−2^, 1 min). Finally, the cells were opened with a razor blade.

### Scattering measurements

The diffuse reflectance and diffuse transmittance spectra were recorded using a Shimadzu UV-3600 spectrophotometer with an integrating sphere configuration ISR-3100 (Supplementary Fig. 4a, b). Total scattering is calculated by summing the diffuse reflectance and diffuse transmittance. The spectra of samples with thicknesses of 50 and 100 μm were measured in a free-standing manner, while the spectra of thinner films were measured with a PVA-coated glass substrate. The spectra of the substrate are shown in Supplementary Fig. 4c, confirming that the scattering due to the substrate can be ignored as it is very small compared to the scattering of the films.

### SEM and SAXS measurements

Scanning electron microscope (SEM) measurements were conducted on a Sigma VP instrument at an accelerating voltage of 3 kV. The samples were frozen in liquid propane, cut in liquid nitrogen, and then freeze-dried to keep the microstructure of the samples, which were coated with 4 nm Au-Pd before the measurements. Small-angle X-ray scattering (SAXS) measurements were performed on the Xeuss 3.0 C scattering device (Xenocs, France) equipped with an EIGER2 R 1 M hybrid pixel detector. A GeniX 3D microfocus X-ray source with a wavelength 1.542 Å was used throughout the experiments. The distance between the sample and detector was set 1100 mm and the LaB_6_ was used to perform the calibration for the sample-detector distance. The samples were sealed in the capillaries during the measurements, and the background was subtracted from the data using XSACT software.

### Kinetics

Transmittance spectra at 400 nm were recorded for the respective LCNs in a self-made glass chamber before and immediately after adding water. The spectra were normalized and fitted with a first-order exponential for estimation of the half-life time for whiteness change.

### CIE plot

CIEXYZ coordinates according to the 1931 standard were calculated from the diffuse reflectance spectra of the 100 µm thick sample in the wet state by utilizing the D55 standard illuminant.

### Instrumentation and characterization details

CoolLED pE-4000 was used as the UV light source (385 nm) for polymerization. FTIR spectra were recorded using an FT-IR Bruker spectrometer, and the contact angle was measured using Attension Theta Lite by Biolin Scientific instrument. Transparent-to-opaque kinetics were measured using an Agilent Cary 60 spectrophotometer by monitoring the 400 nm wavelength. Stress−strain curves were determined by using a homemade tensile tester using 100-μm-thick films (stretching speed of 0.05 mm/s). All the pictures and movies were captured using a Canon 5D Mark III camera.

### Fracture energy calculations

The fracture energy was calculated according to *G*_*c*_ = 6*wc*/ √ λ_*c*_, where *c* is the crack length (0.5 mm), and *λ*_*c*_ is the elongation at break of the notched sample. The strain energy *w* was obtained by integrating the stress–strain curve of the original unnotched sample up to the fracture strain *ε*_*c*_, where *ε*_*c*_ = *λ*_*c*_.

### Characteristic attenuation length

The characteristic attenuation length, *l*_s_, was determined by fitting the thickness dependence of the total transmission to an exponential attenuation relation, *T*_total_ = *e* ^*−L/ls*^, where *T*_total_ is the measured total transmittance and *L* is the sample thickness. Accordingly, ln(*T*_total_) was plotted as a function of sample thickness for wet LCN_AA+DMA+I_ films. The linear fit yielded a slope of 0.00857 μm^−1^, corresponding to a characteristic attenuation length of 116 μm.

### Preparation of coil for detecting underwater objects demonstration

The molten mixture of configuration LCN_AA+DMA+I_ and LCN_AA_ with 2 wt% photoinitiator was poured into a transparent tube (length: 15 cm, outer diameter: 1.4 mm) and coiled around an NMR tube. Further, it polymerized under UV light (385 nm, 35 mW cm^−2^) at 60 °C. The solidified coil was removed after cooling by cutting the tube.

## Supplementary information

Supplementary Information

Description of Additional Supplementary Files

Supplementary Movie 1

Transparent Peer Review file

## Data Availability

All data are available from the corresponding author upon request. Source data file has been deposited in Figshare under accession code https://doi.org/10.6084/m9.figshare.32211408.
