# Supplementary material for: Switchable whiteness in liquid crystal networks via solvent-induced microphase segregation
Source: Nat Commun. 2026 Sep 8;17:10625. doi: 10.1038/s41467-026-77442-y (PMC13649656; doi:10.1038/s41467-026-77442-y)
Supplement: Supplementary file 1 [file 41467_2026_77442_MOESM1_ESM.pdf]

Supplementary Information for

Switchable Whiteness in Liquid Crystal Networks via Solvent-Induced Microphase Segregation

Roshan Nasare[1], Hilma Panula[1], Chen Liang[2], Tero-Petri Ruoko[1], Hang Zhang[2,3], Hongshuang Guo[1]*, and Arri Priimagi[1]*

Affiliation:

[1] Faculty of Engineering and Natural Sciences, Tampere University, Tampere, P.O. Box 541, FI-33101, Finland.

[2] Department of Applied Physics, Aalto University, P.O. Box 15100, FI 02150 Espoo, Finland.

[3]Present address: Department of Biomedical Engineering, College of Engineering, Eastern Institute of Technology, Ningbo, China.

*Correspondence to: hongshuang.guo@tuni.fi; arri.priimagi@tuni.fi.

This PDF file includes:

1. **Supplementary Figures 1-26**
2. **Supplementary Table 1-7**
3. **References**

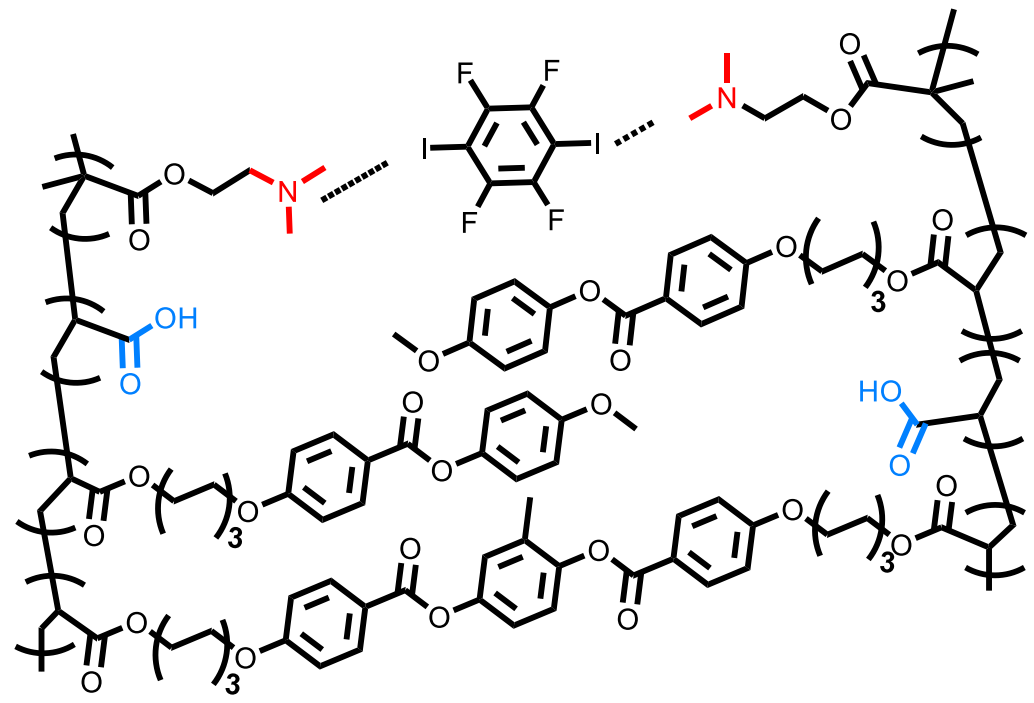

Supplementary Figure 1. Schematic of chemical structure of $LCN_{AA+DMA+I}$ polymer film.

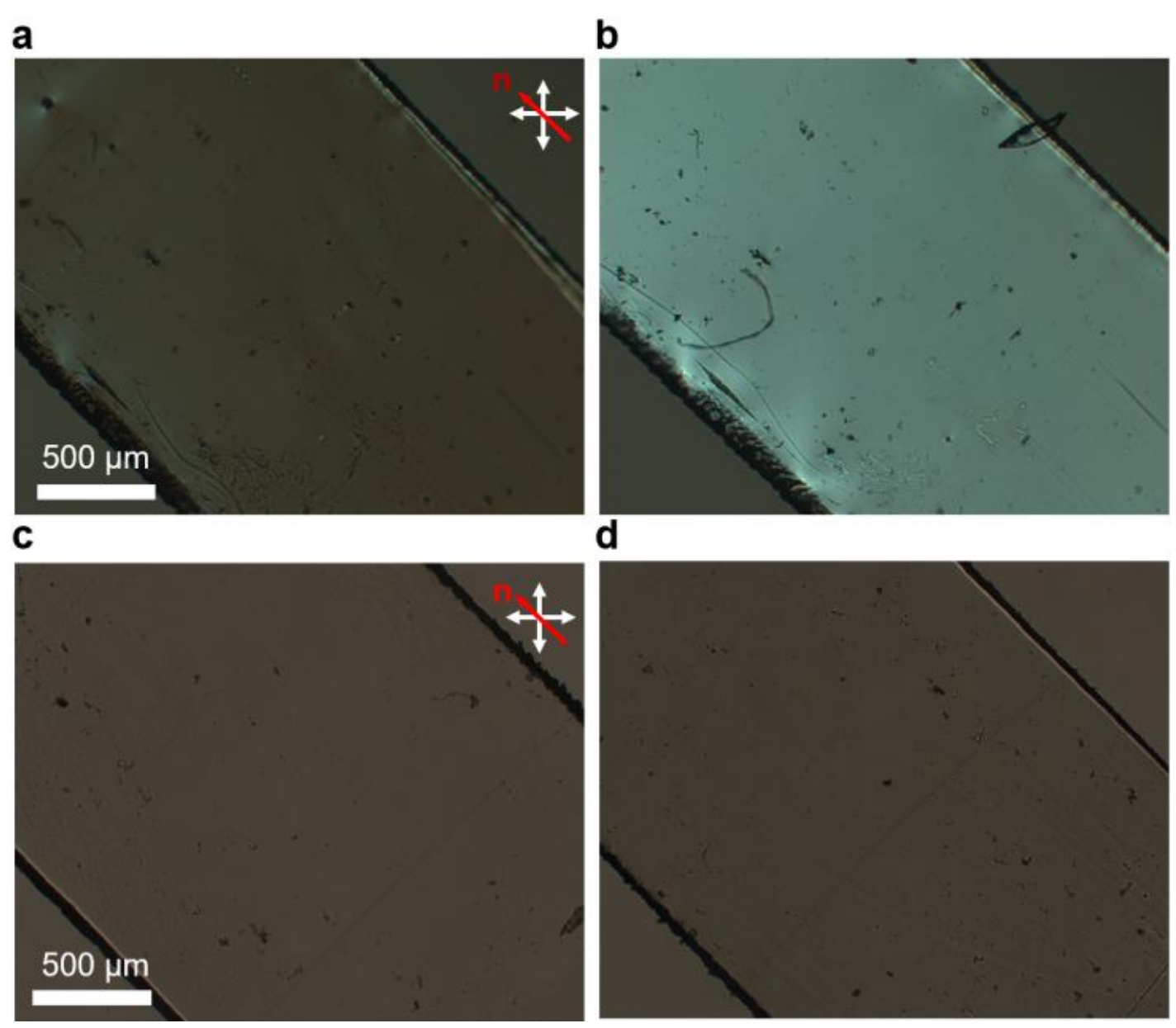

Supplementary Figure 2. Polarized optical micrographs of $LCN_{AA+DMA+I}$ film (a) before, and (b) after 5-10 % stretching, and for Network 2 (c) before, and (d) after 5-10% stretching. Film thicknesses: 100 µm.

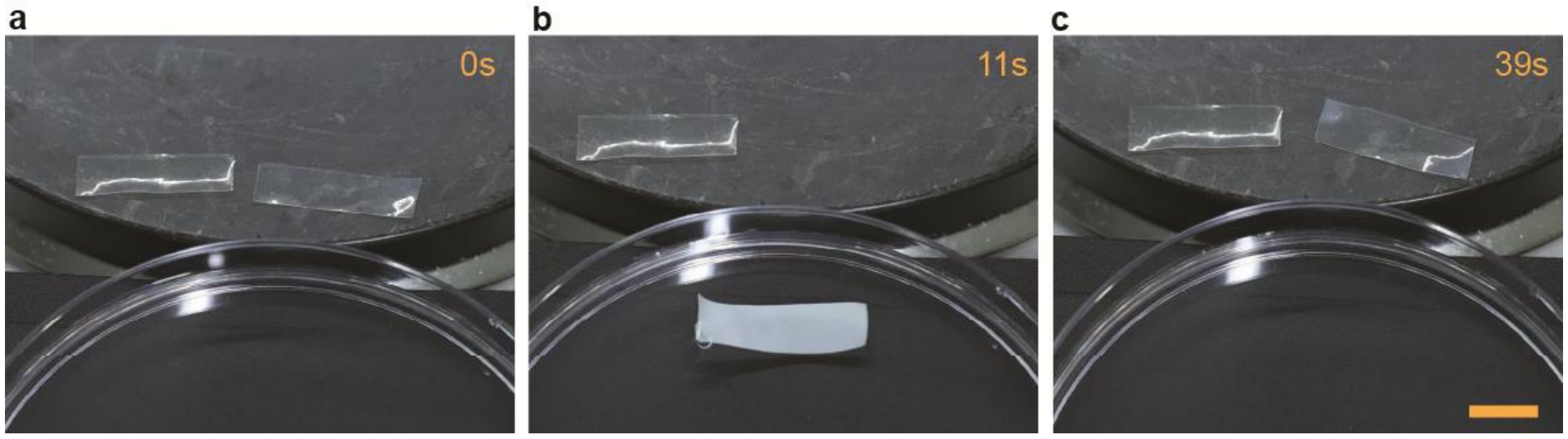

Supplementary Figure 3. Photographs of $LCN_{AA+DMA+I}$ upon wetting and drying. (a) Two identical $LCN_{AA+DMA+I}$ films in a dry and transparent state, (b) One of the films is dipped in water, showing whiteness, (c) After drying the wet film on a hot plate, the film again becomes transparent. Film thickness: 100 µm. Scale bar: 1 cm.

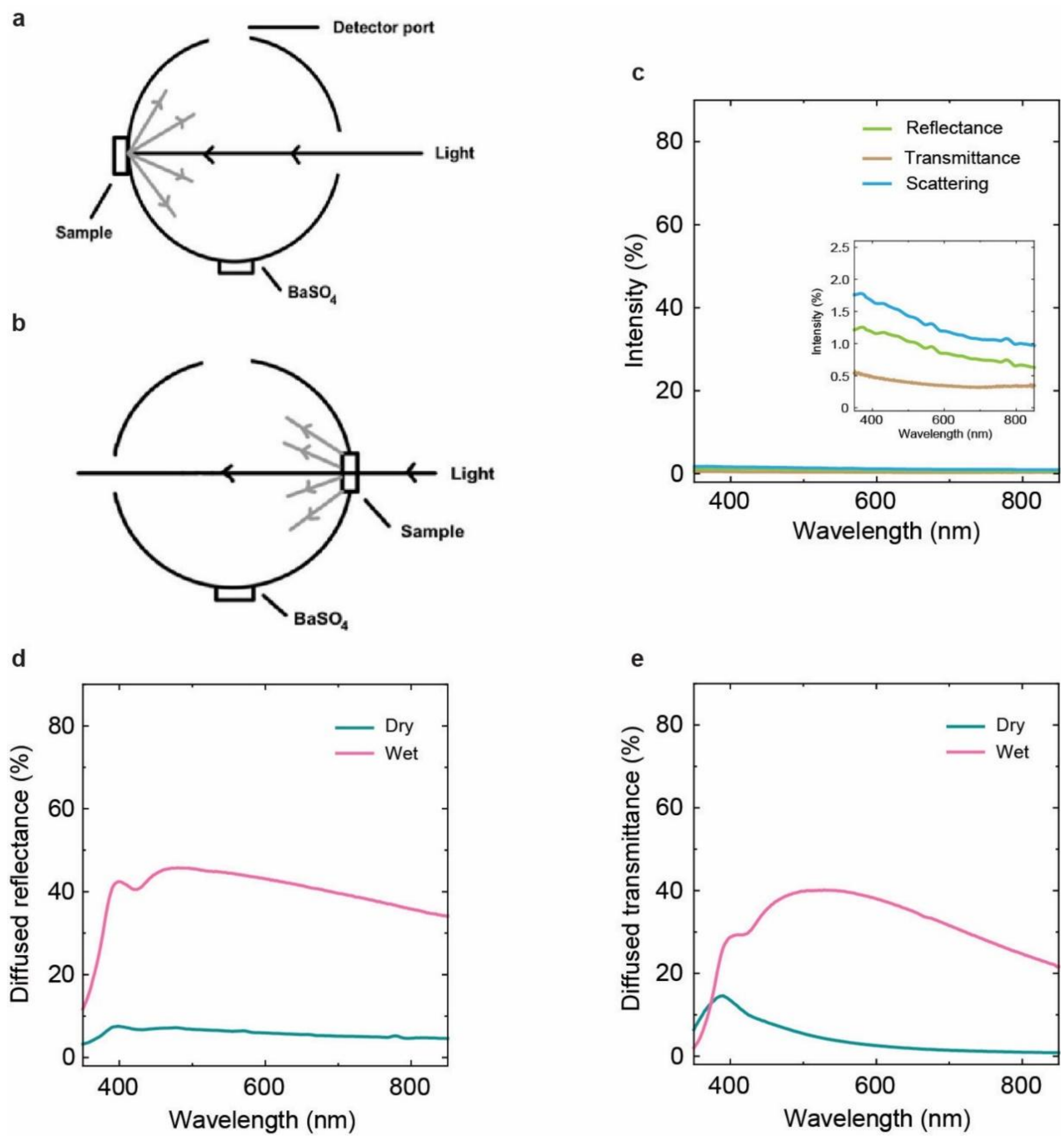

Supplementary Figure 4. Schematics of (a) diffused reflectance and (b) diffused transmittance measurements using an integrating sphere. (c) Spectra of PVA-coated glass showing diffuse reflectance, diffuse transmittance, and total scattering. (d) diffused reflectance, and (e) diffused transmittance spectra of $LCN_{AA+DMA+I}$. Film thickness: 100 μm.

Supplementary Table 1. Different composition of reagents in mmol, varying the ratio of the AA and DMA, while maintaining a fixed content of LC monomers and XB between DMA and 1,4-I in 2:1.

	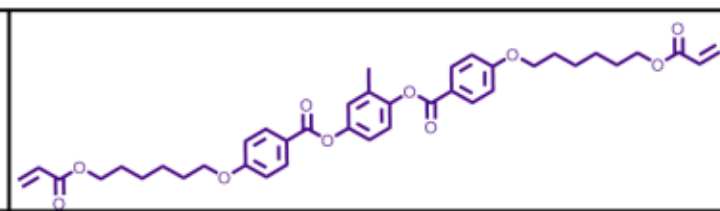	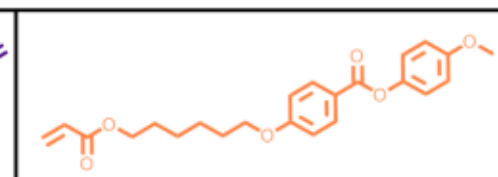	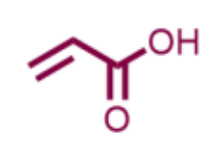	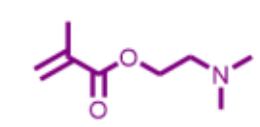	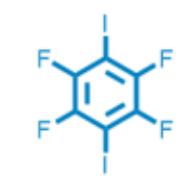
LCN$_{AA}$	3	15	150	0	0
LCN$_{AA+DMA+I}$	3	15	100	50	25
LCN$_{DMA+I}$	3	15	0	150	75

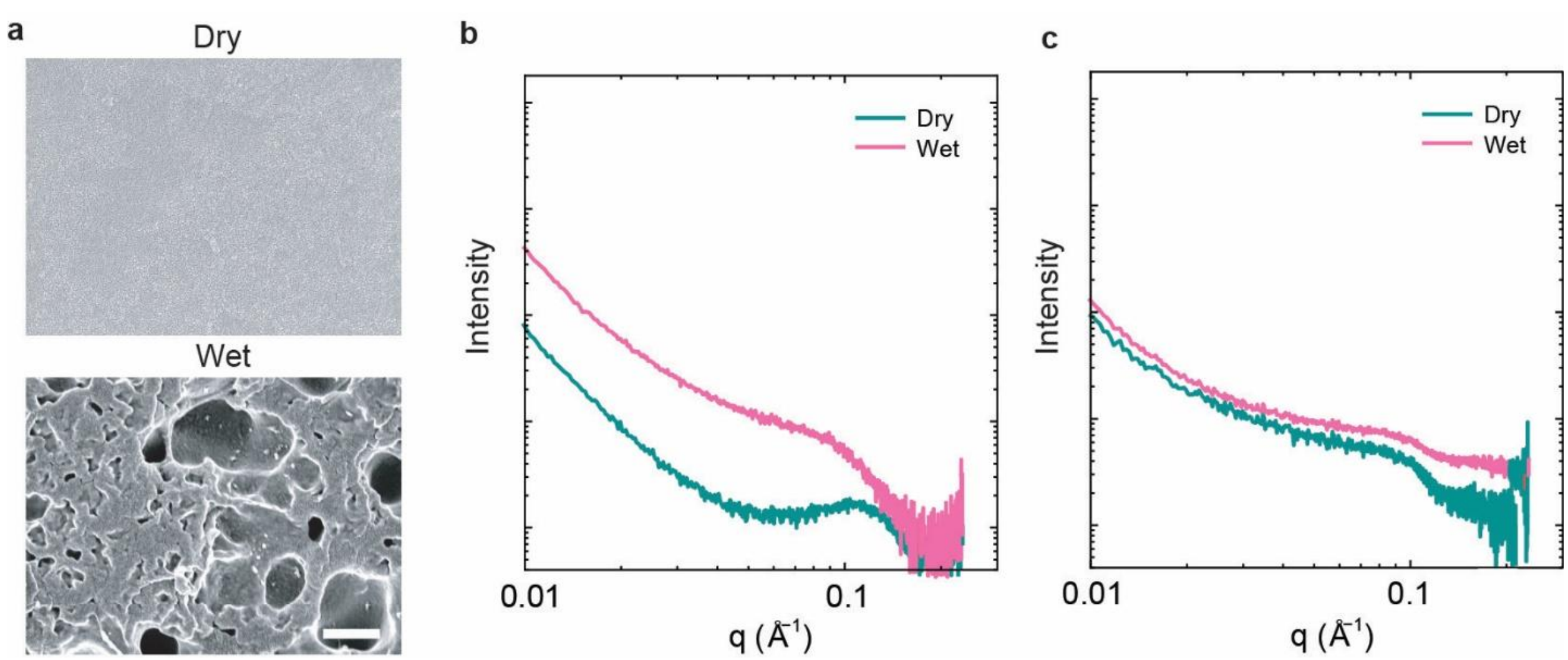

Supplementary Figure 5. SEM of (a) LCN$_{AA+DMA}$, Scale bar: 800nm; SAXS of (b) LCN$_{AA+DMA}$ confirming phase separation in the wet state, while no phase separation can be seen in SAXS of (c) LCN$_{AA}$. Film thickness: 100 μm

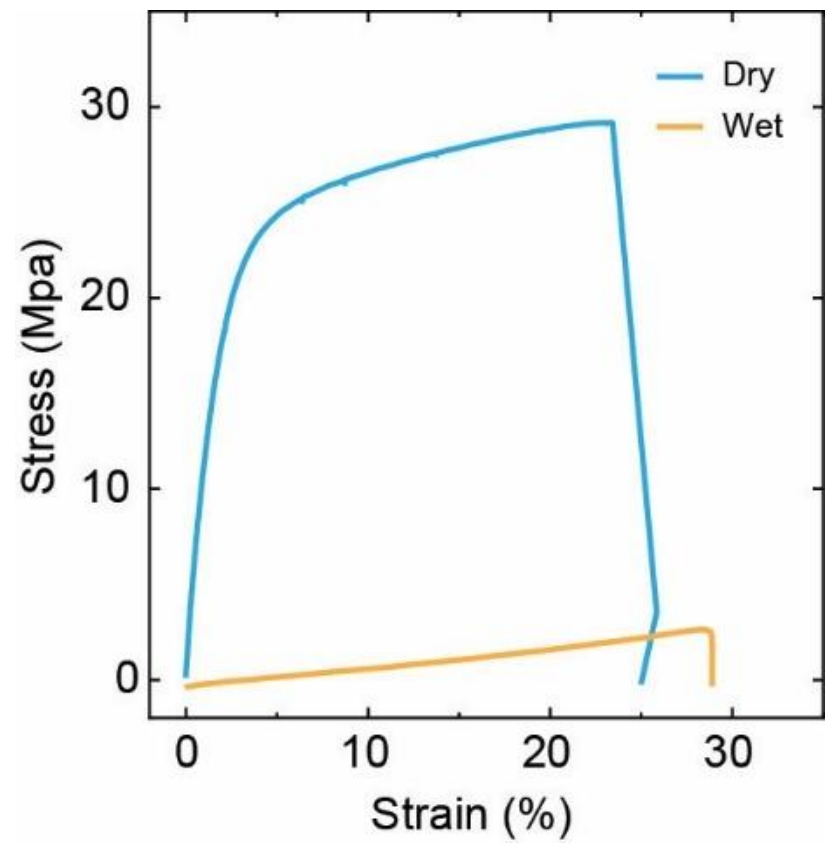

Supplementary Figure 6. Tensile stress–strain curves in the dry (20% RH, 20 ºC) and wet state of $LCN_{AA+DMA+I}$ film. Film thickness: 100 µm.

Supplementary Table 2. Describing Young's modulus, fracture strain, and tensile strength. The values are averaged over n=3 measurements with SD. All sample thickness 100 µm

Sample	$\mathbf{E_Y}$ (MPa)	$\boldsymbol{\sigma}_{\mathbf{max}}$ (MPa)	$\boldsymbol{\varepsilon}_{\mathbf{max}}$ (%)
$LCN_{AA+DMA+I}$	594.2±16.6	26.9±4.0	27.8±5.4
$LCN_{AA+DMA+I}$ wet	12.4±3.1	3.7±1.7	30.7±5.3

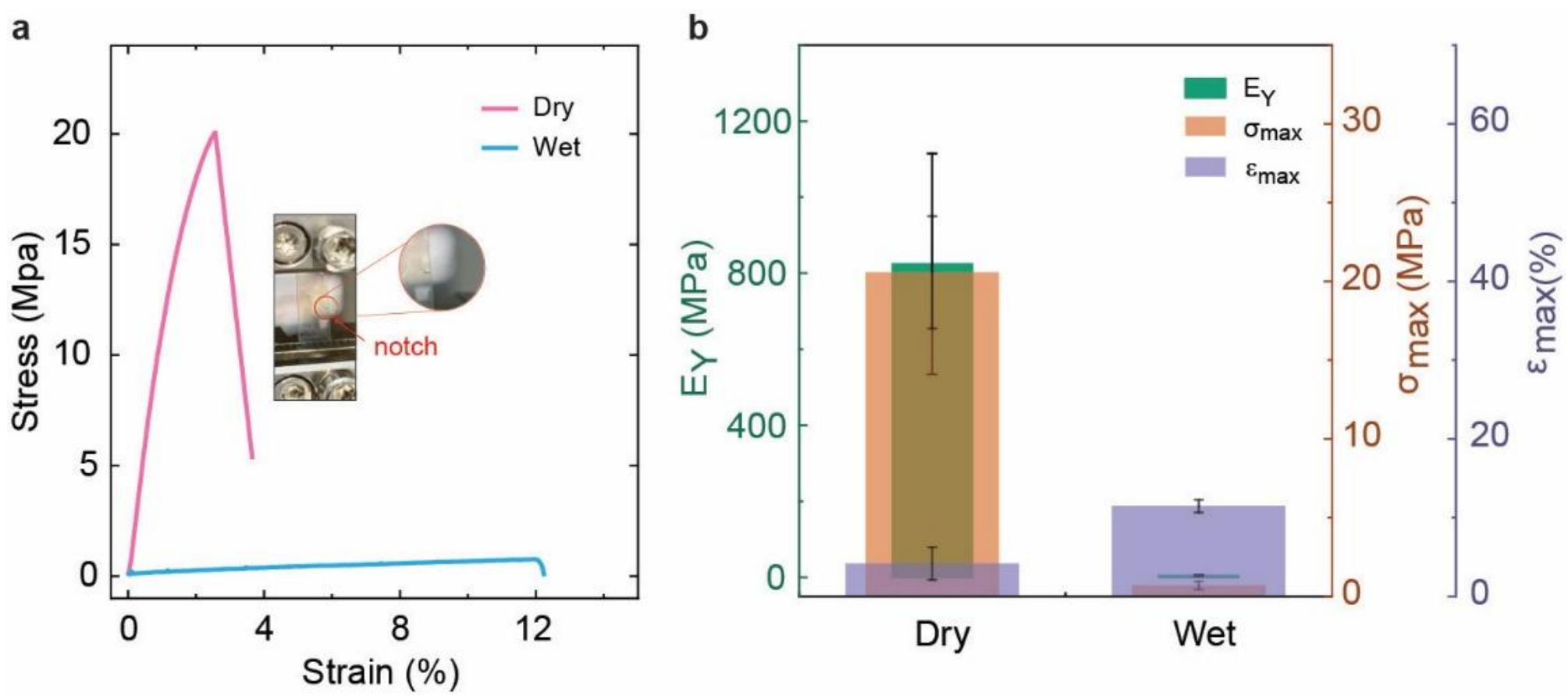

Supplementary Figure 7. (a) Tensile stress–strain curves of notched $LCN_{AA+DMA+I}$ film in the dry and wet states. Sample width: 5 mm; Notch width 0.5 mm. (b) Corresponding values for Young's modulus, tensile strength, and fracture strain. Error bars represent the SD obtained from n=3 measurements. Film thickness: 100 μm.

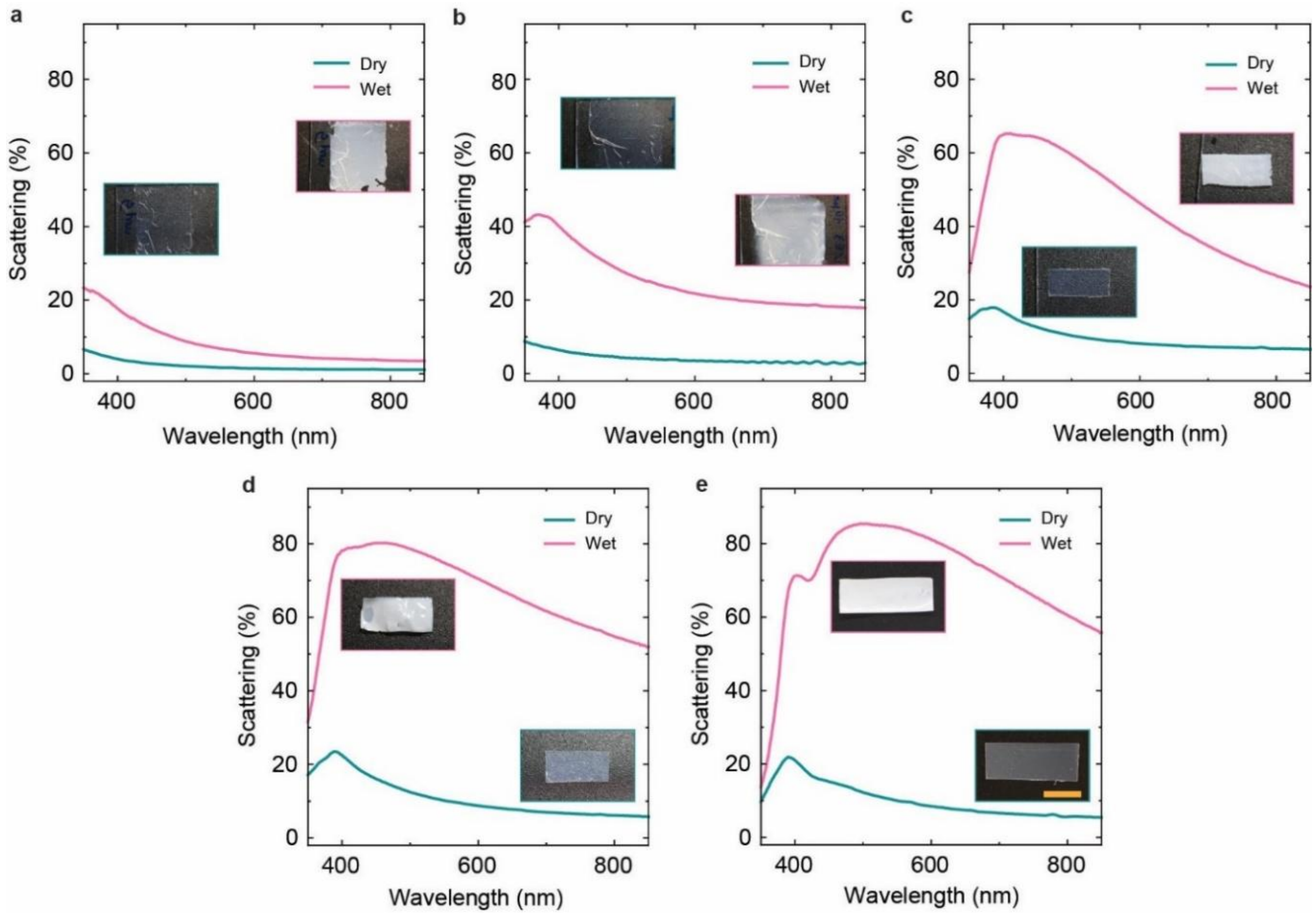

Supplementary Figure 8. Dry and wet state total scattering spectra of $LCN_{AA+DMA+I}$ with different thicknesses. (a) 5 µm, (b) 10 µm, (c) 20 µm, (d) 50 µm, and (e) 100 µm. Insets are the transparent and opaque photographs of respective thicknesses. Scale bar: 1 cm

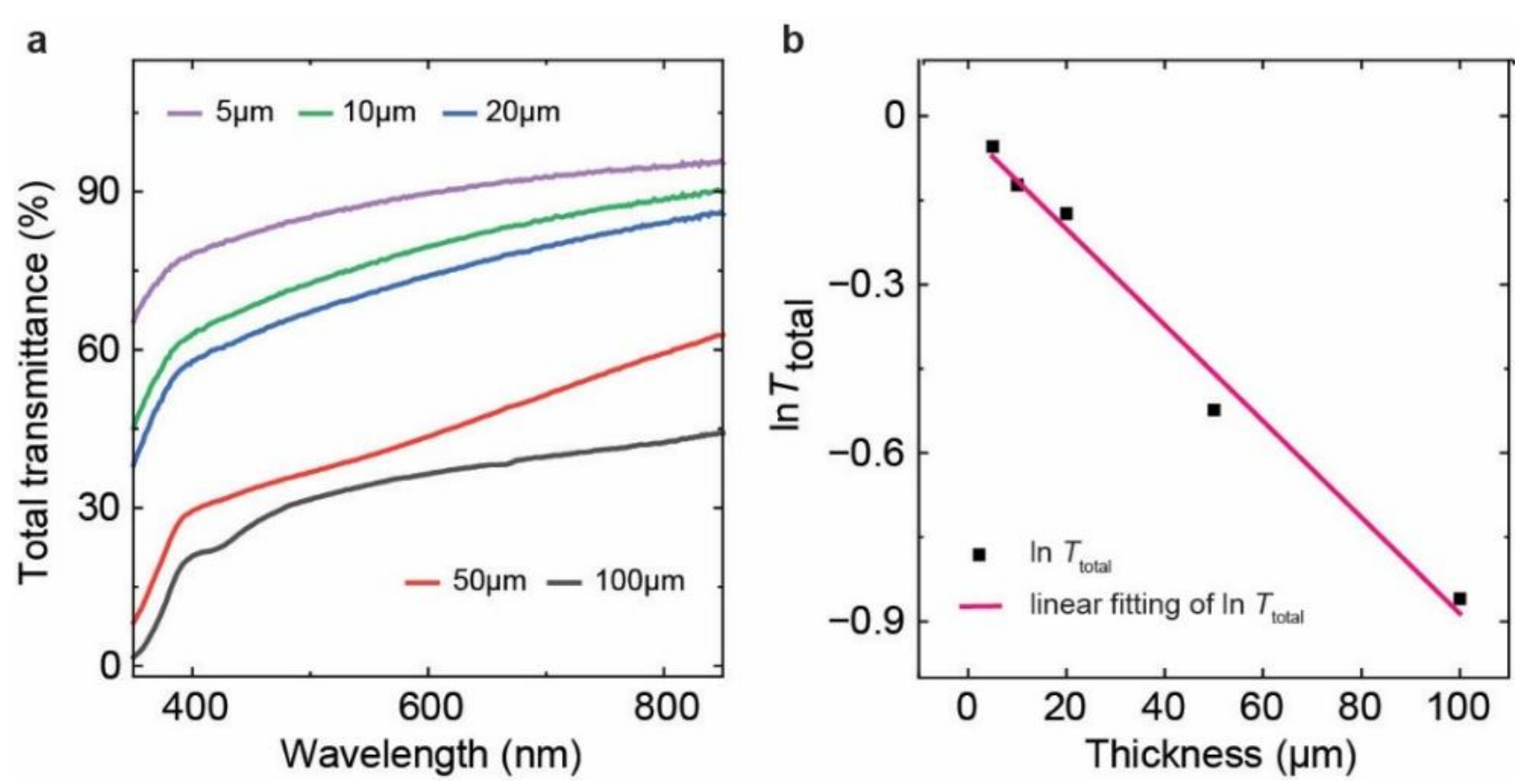

Supplementary Figure 9. (a) Total transmittance spectra of $LCN_{AA+DMA+I}$ film in wet state of different thicknesses. (b) Fitting between the ln (T_{total}) and the film thickness at 800 nm wavelength.

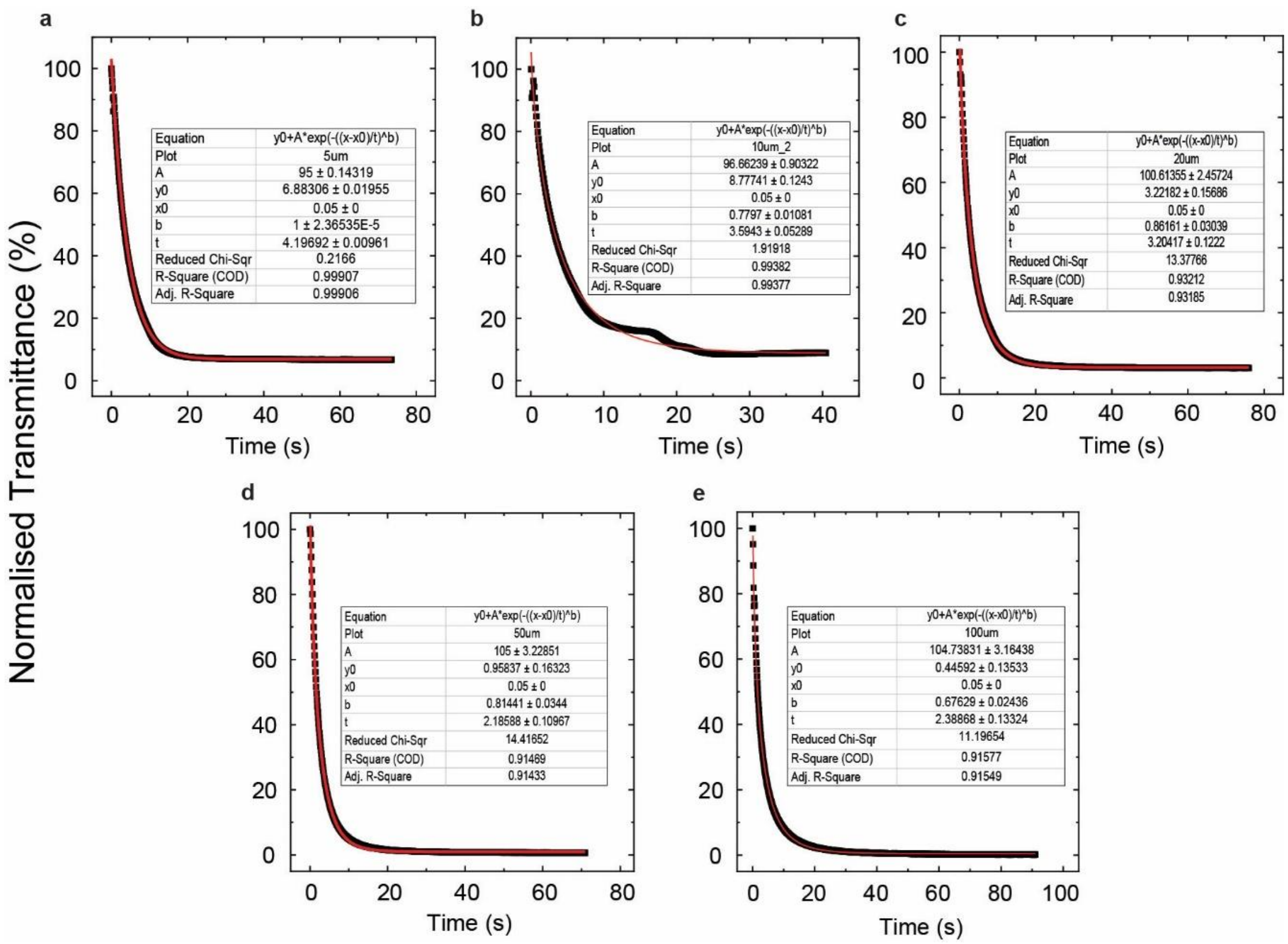

Supplementary Figure 10. Kinetics of transparent-to-white transition for $LCN_{AA+DMA+I}$ films of different thicknesses: (a) 5 µm, (b) 10 µm, (c) 20 µm, (d) 50 µm, and (e) 100 µm, via monitoring transmittance changes at 400 nm at 25 °C.

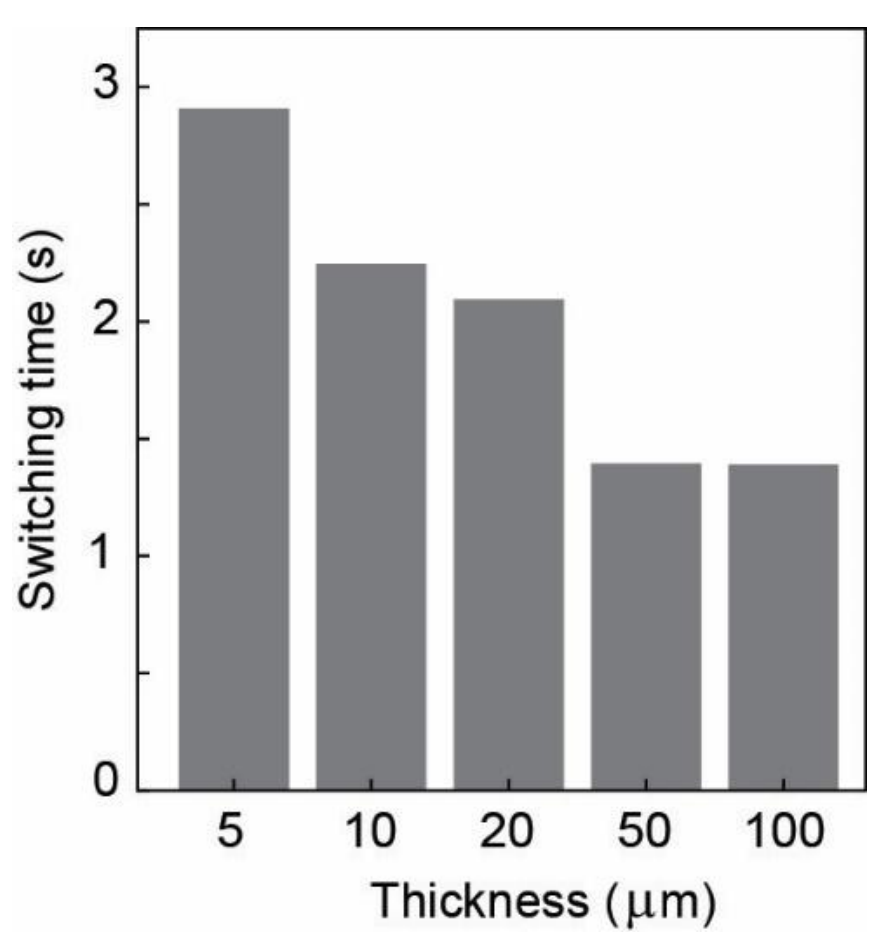

Supplementary Figure 11. Switching time in terms of half-lives for $LCN_{AA+DMA+I}$ films of different thickness, extracted from the data above.

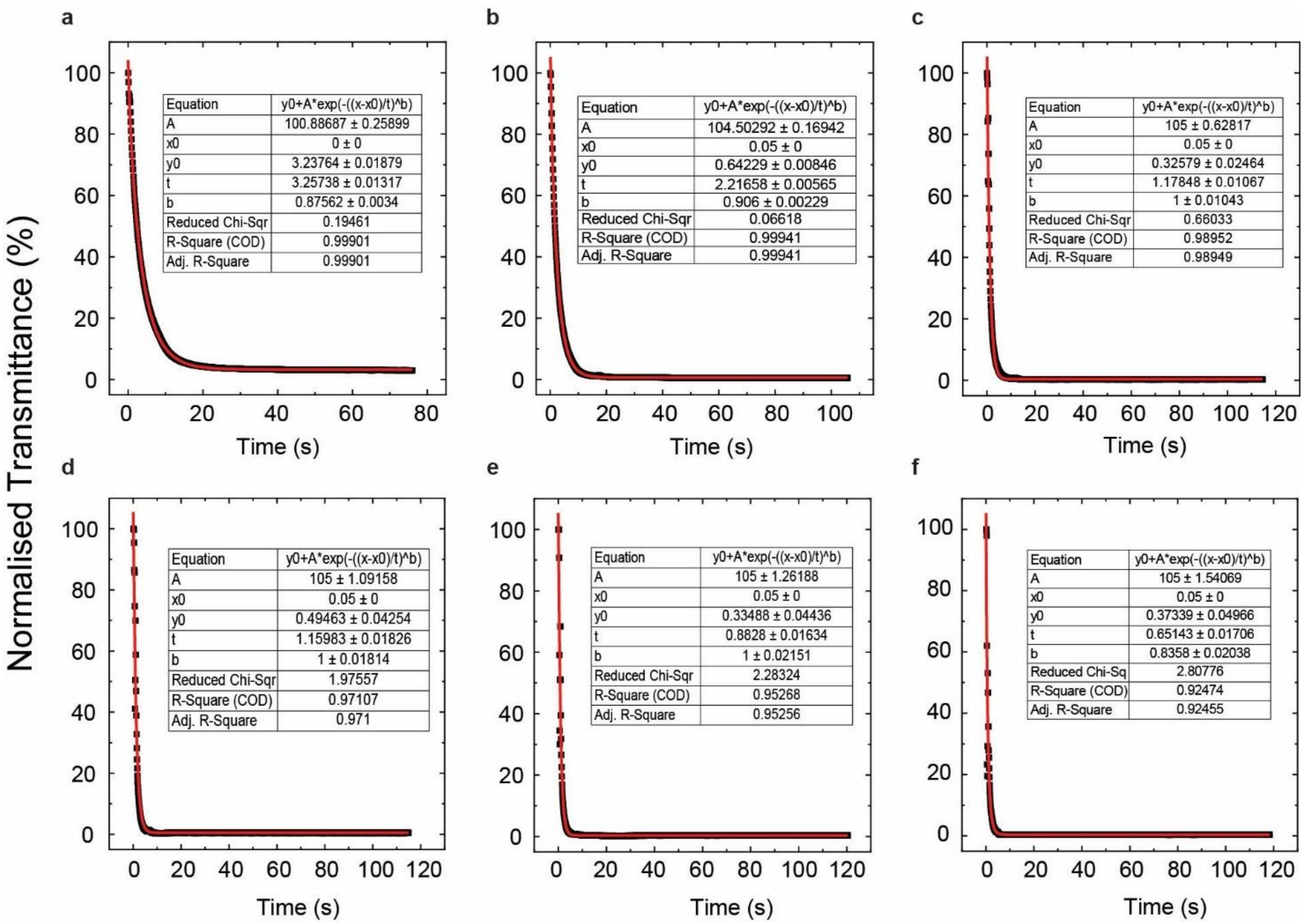

Supplementary Figure 12. Kinetics of transparent-to-white transition for 20 µm $LCN_{AA+DMA+I}$ film at different temperatures: (a) 25 ºC, (b) 30 ºC, (c) 40 ºC, (d) 60 ºC, (e) 80 ºC, and (f) 85 ºC, via monitoring transmittance changes at 400 nm.

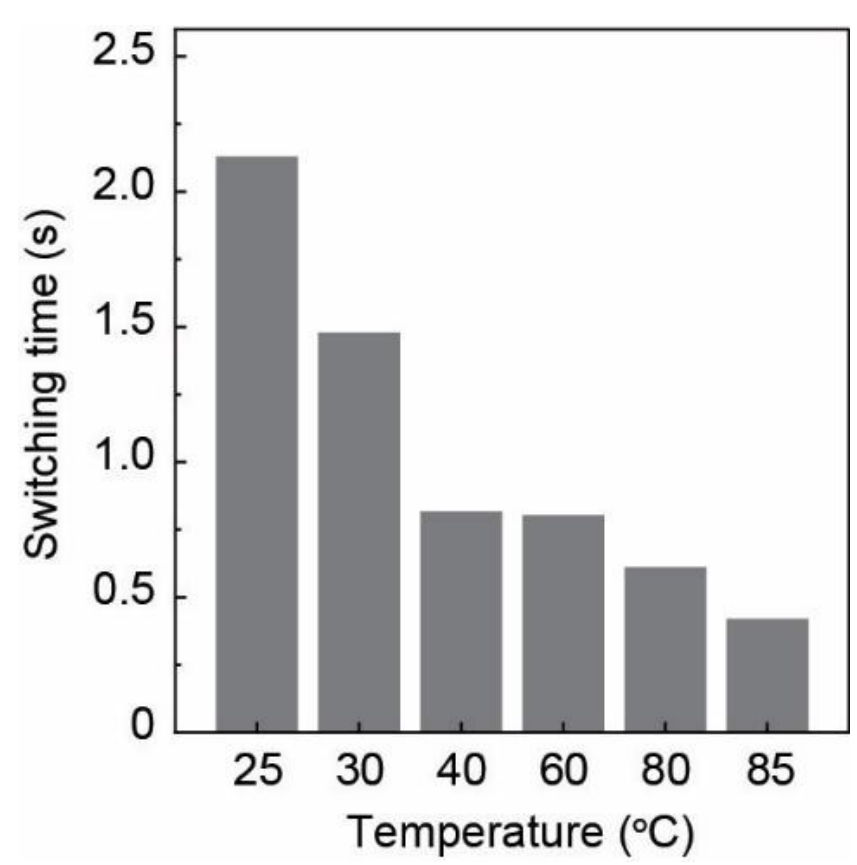

Supplementary Figure 13. Switching time in terms of half-lives for the 20 µm $LCN_{AA+DMA+I}$ film at different temperatures, extracted from the data above.

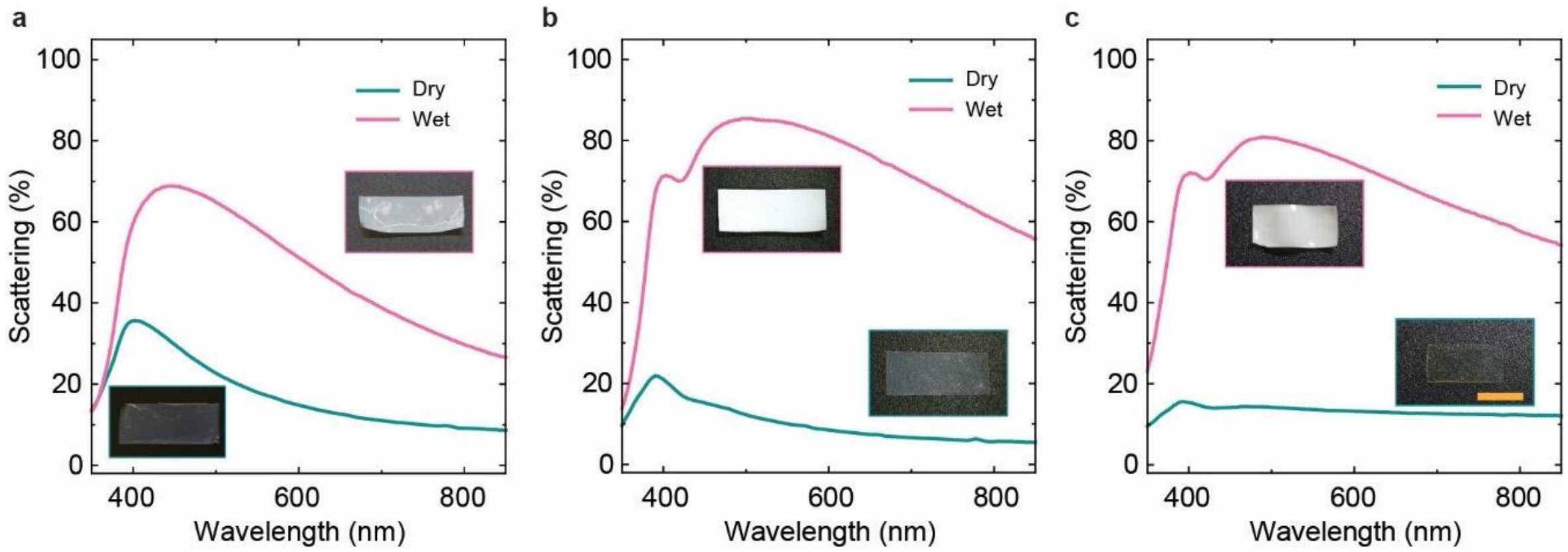

Supplementary Figure 14. Total scattering spectra of (a) LCN 1, (b) $LCN_{AA+DMA+I}$, (c) LCN 2. Insets are the transparent and opaque photographs of respective LCNs. Film thickness: 100 µm. Scale bar: 1 cm.

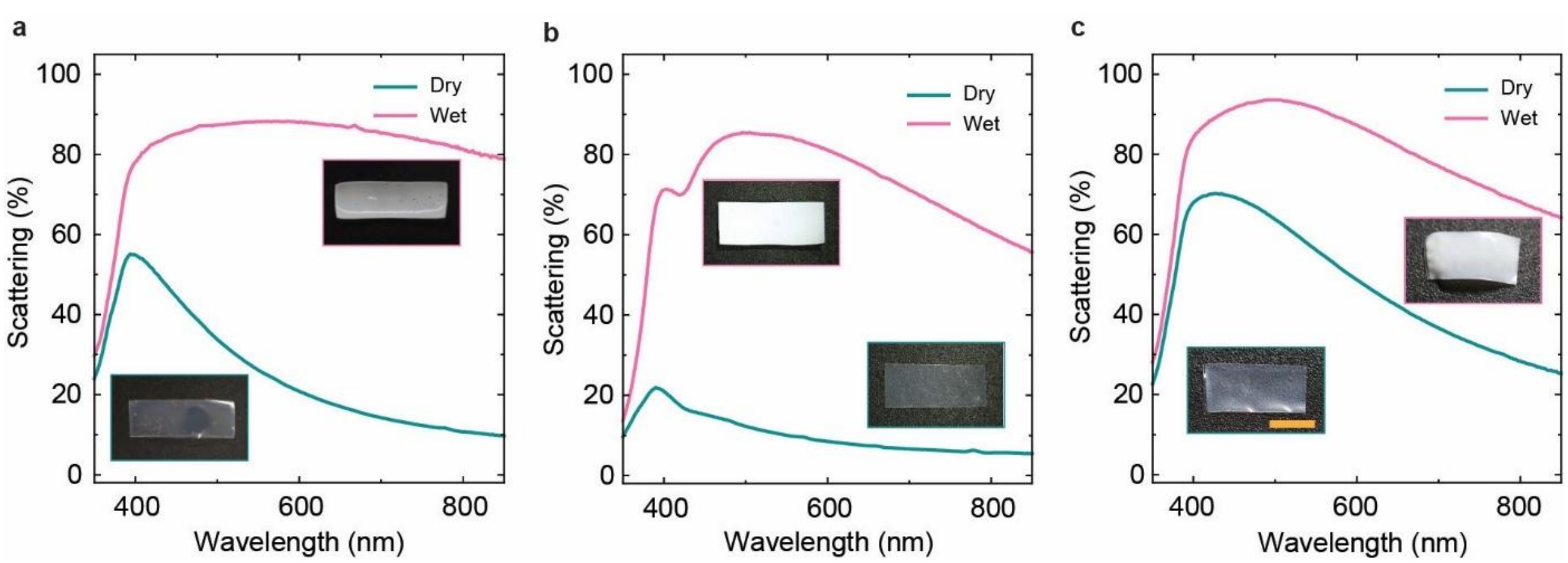

Supplementary Figure 15. Total scattering spectra of (a) $LCN_{AA+DMA+F}$, (b) $LCN_{AA+DMA+I}$, (c) LCN_{AA+DMA}. Insets are the transparent and opaque photographs of respective LCNs. Film thickness: 100 µm. Scale bar: 1 cm.

Supplementary Table 3. Different donor concentrations used in mmol with 1,4-I as a donor

	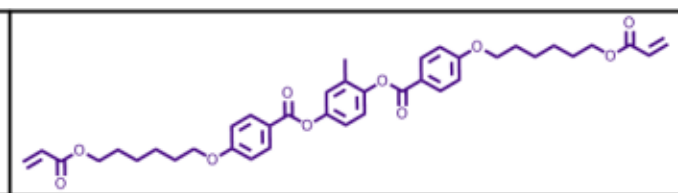	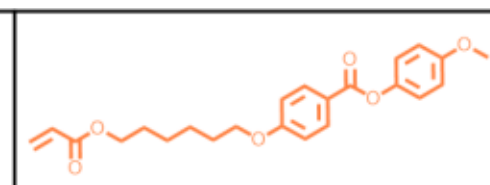	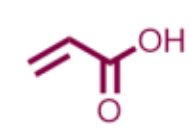	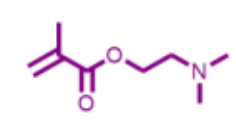	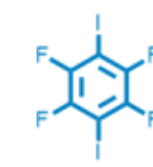
LCN_{AA+DMA}	3	15	100	50	0
$LCN_{AA+DMA+I}$ 1:5	3	15	100	50	10
$LCN_{AA+DMA+I}$	3	15	100	50	25
$LCN_{AA+DMA+I}$ 1:1	3	15	100	50	50
$LCN_{AA+DMA+I}$ 3:2	3	15	100	50	75

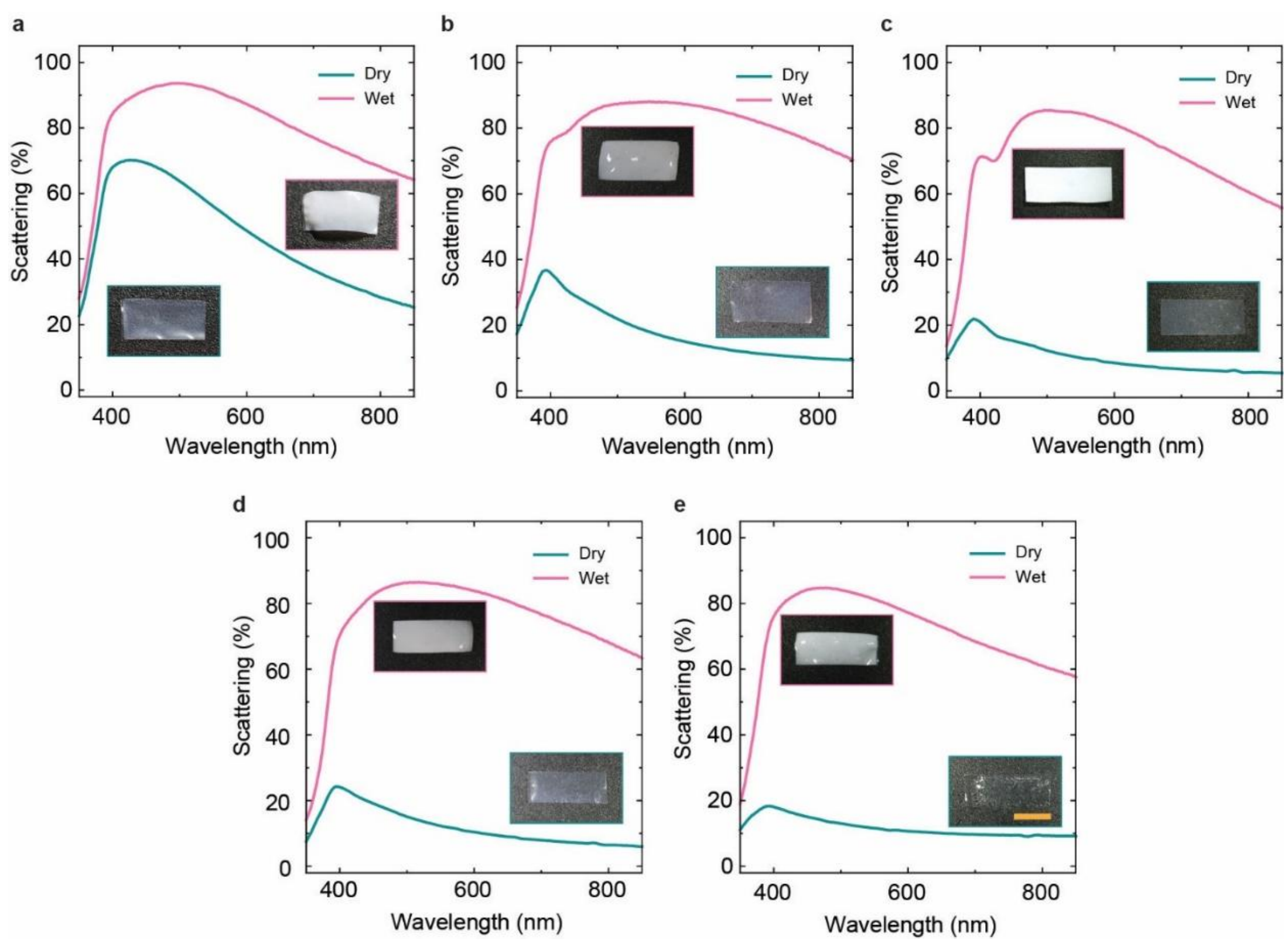

Supplementary Figure 16. Total scattering spectra of (a) LCN_{AA+DMA}, (b) $LCN_{AA+DMA+I}$ 1:5, (c) $LCN_{AA+DMA+I}$, (d) $LCN_{AA+DMA+I}$ 1:1, (e) $LCN_{AA+DMA+I}$ 3:2. Insets are the transparent and opaque photographs of respective LCNs. Film thickness: 100 µm. Scale bar: 1 cm.

Supplementary Table 4. Different compositions used in mmol.

	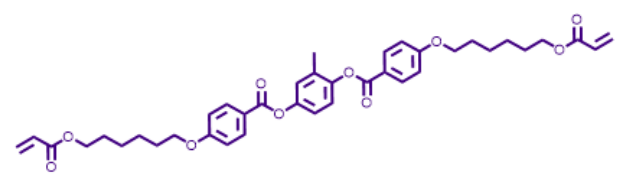	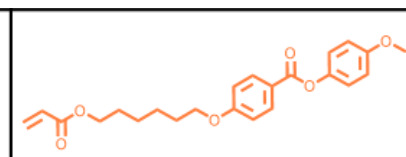	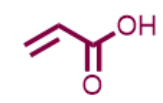	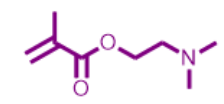	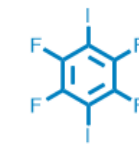
LCN CT 1	3	-	100	50	25
CT 2	-	15	100	50	25
CT 3	-	-	100	50	25
CT 4	-	-	100	50	-
LCN CT 5	13	55	100	-	25
	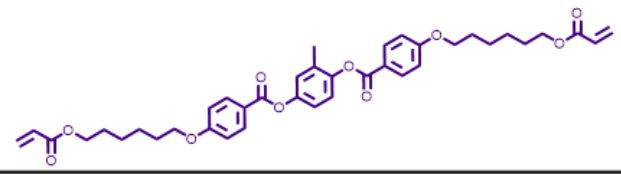	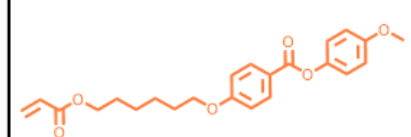	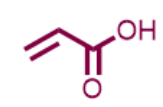	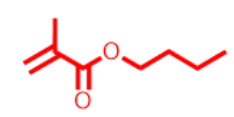	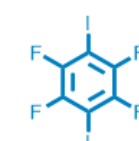
LCN CT 6	3	15	100	50	25
	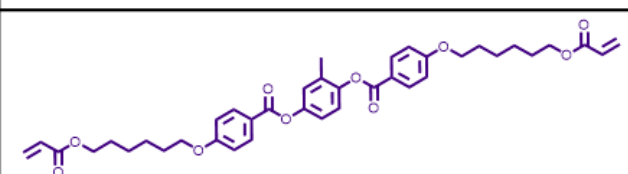	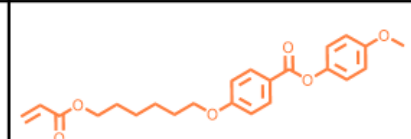	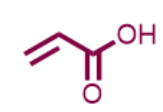	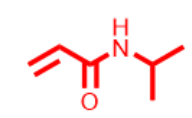	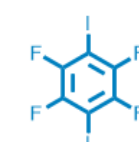
LCN CT 7	3	15	100	50	25

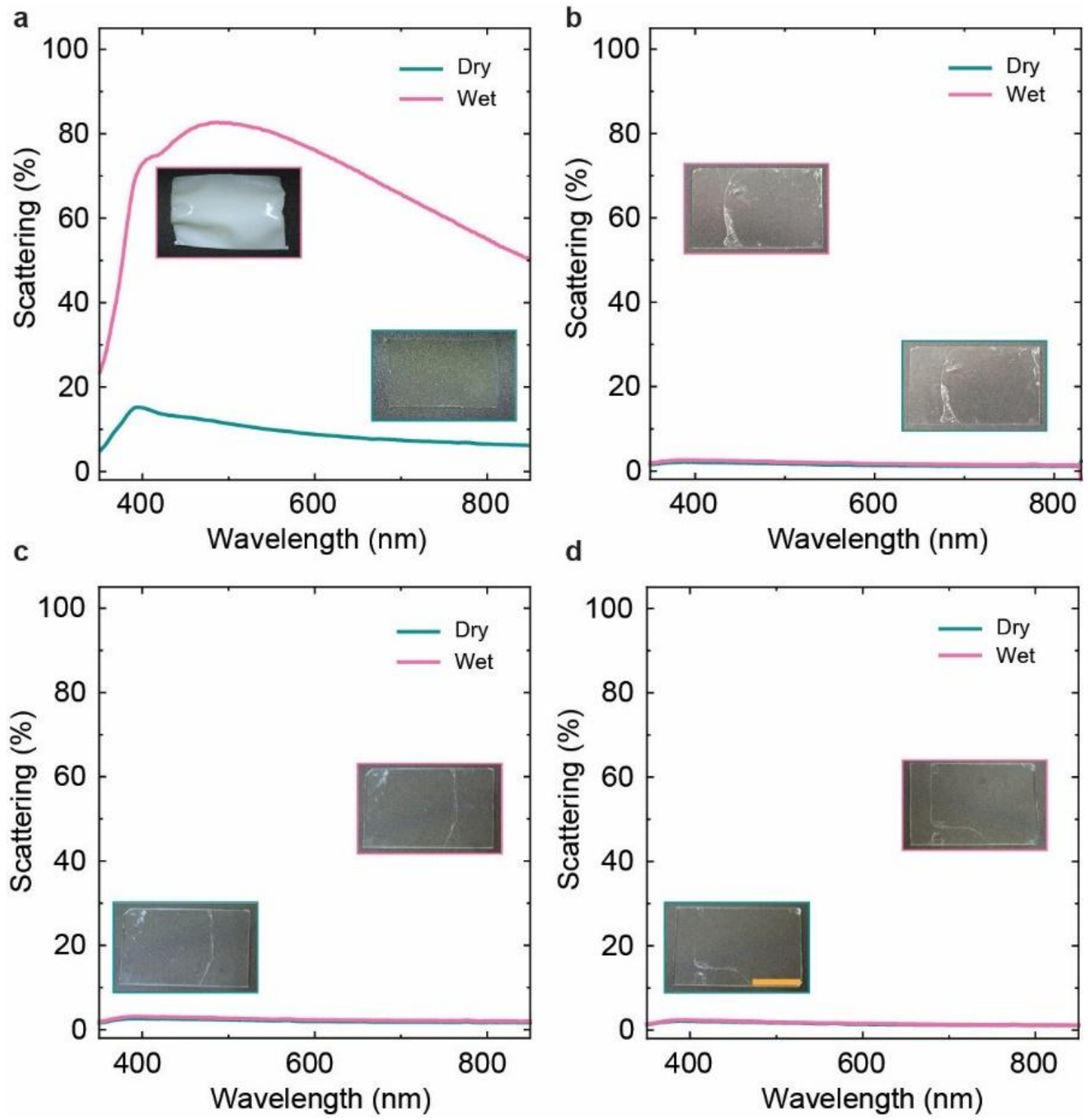

Supplementary Figure 17. Total scattering spectra of (a) LCN CT 1, (b) LCN CT 5, (c) LCN CT 6, (d) LCN CT 7. Insets are the transparent and opaque photographs of respective LCNs. Thickness: 100 μm. Scale bar: 1 cm.

Supplementary Table 5. Different composition of LC concentration used in mmol

	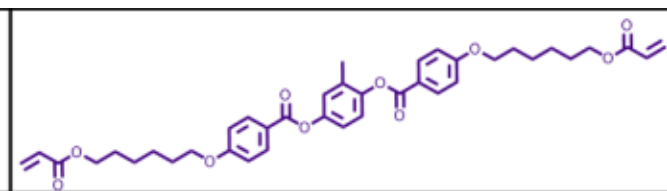	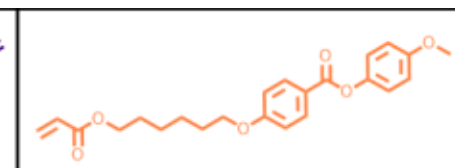	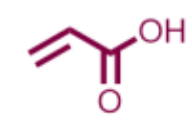	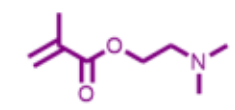	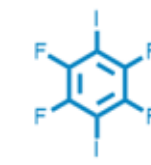
$LCN_{AA+DMA+I}$ 5%	1	7	100	50	25
$LCN_{AA+DMA+I}$	3	15	100	50	25
$LCN_{AA+DMA+I}$ 15%	5	22	100	50	25
$LCN_{AA+DMA+I}$ 20%	7.5	30	100	50	25
$LCN_{AA+DMA+I}$ 25%	10	40	100	50	25
$LCN_{AA+DMA+I}$ 30%	12.5	52	100	50	25

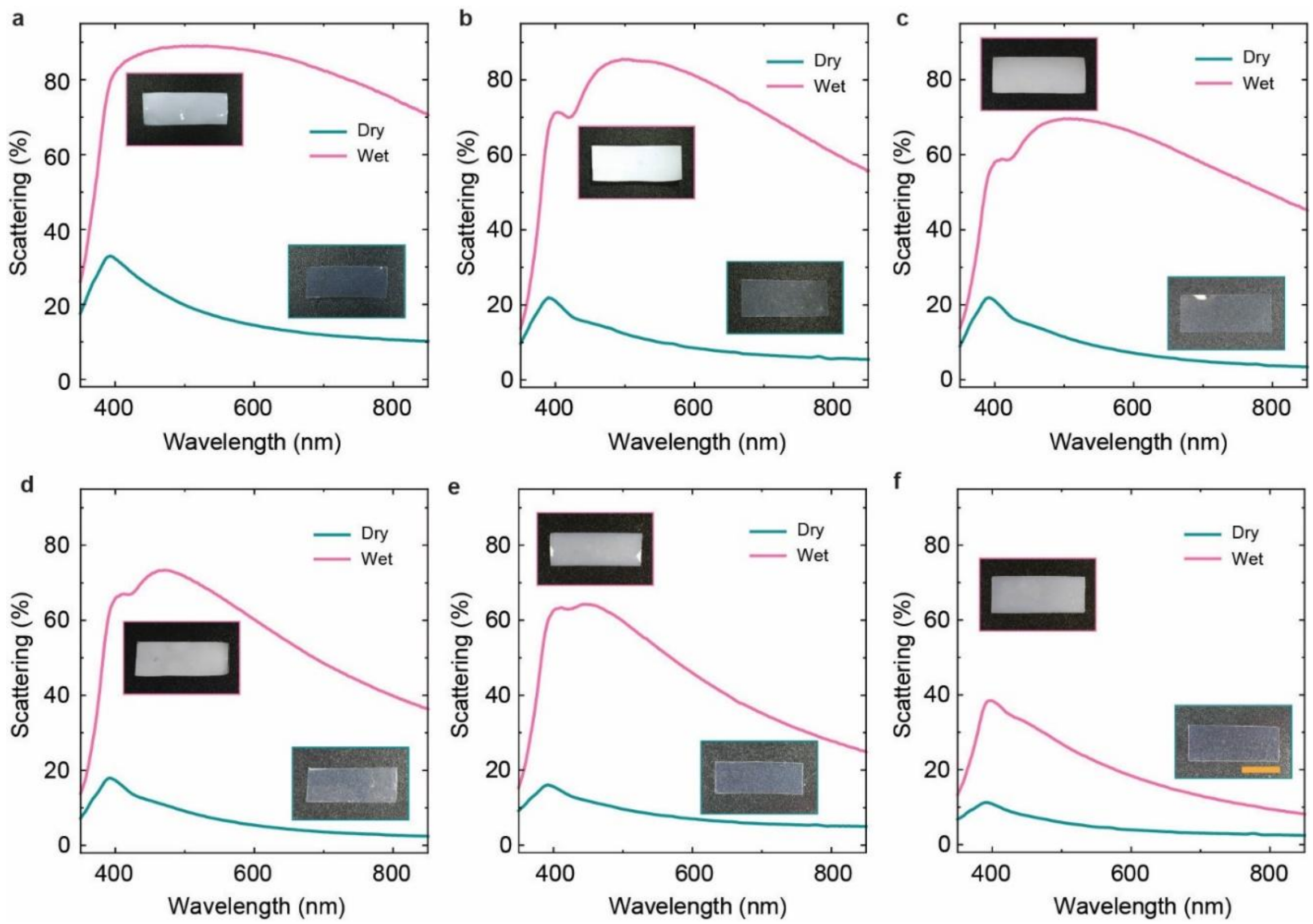

Supplementary Figure 18. Total scattering spectra of (a) $LCN_{AA+DMA+I}$ 5%, (b) $LCN_{AA+DMA+I}$, (c) $LCN_{AA+DMA+I}$ 15%, (d) $LCN_{AA+DMA+I}$ 20%, (e) $LCN_{AA+DMA+I}$ 25%, (f) $LCN_{AA+DMA+I}$ 30%. Insets are the transparent and opaque photographs of respective LCNs. Thickness: 100 µm. Scale bar: 1 cm.

Supplementary Table 6. Compositions (in mmol) of the non-liquid-crystalline reference polymer networks

	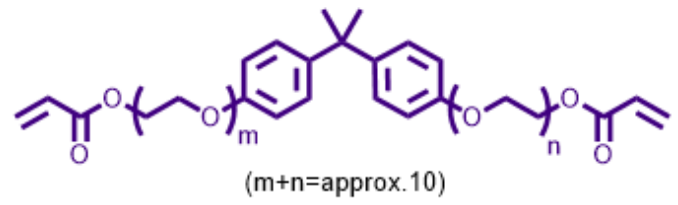	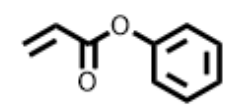	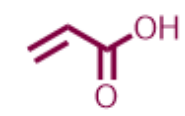	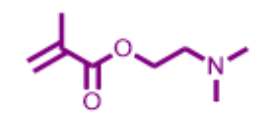	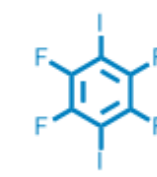
Network 1	3	-	100	50	25
Network 2	3	15	100	50	25
	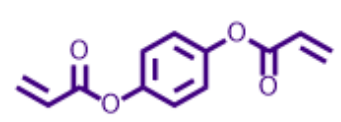	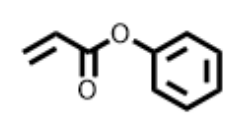	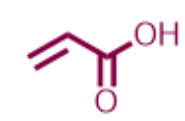	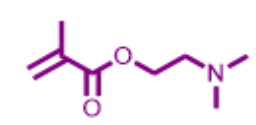	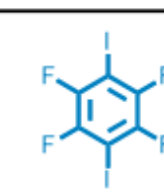
Network 3	3	-	100	50	25
Network 4	3	15	100	50	25

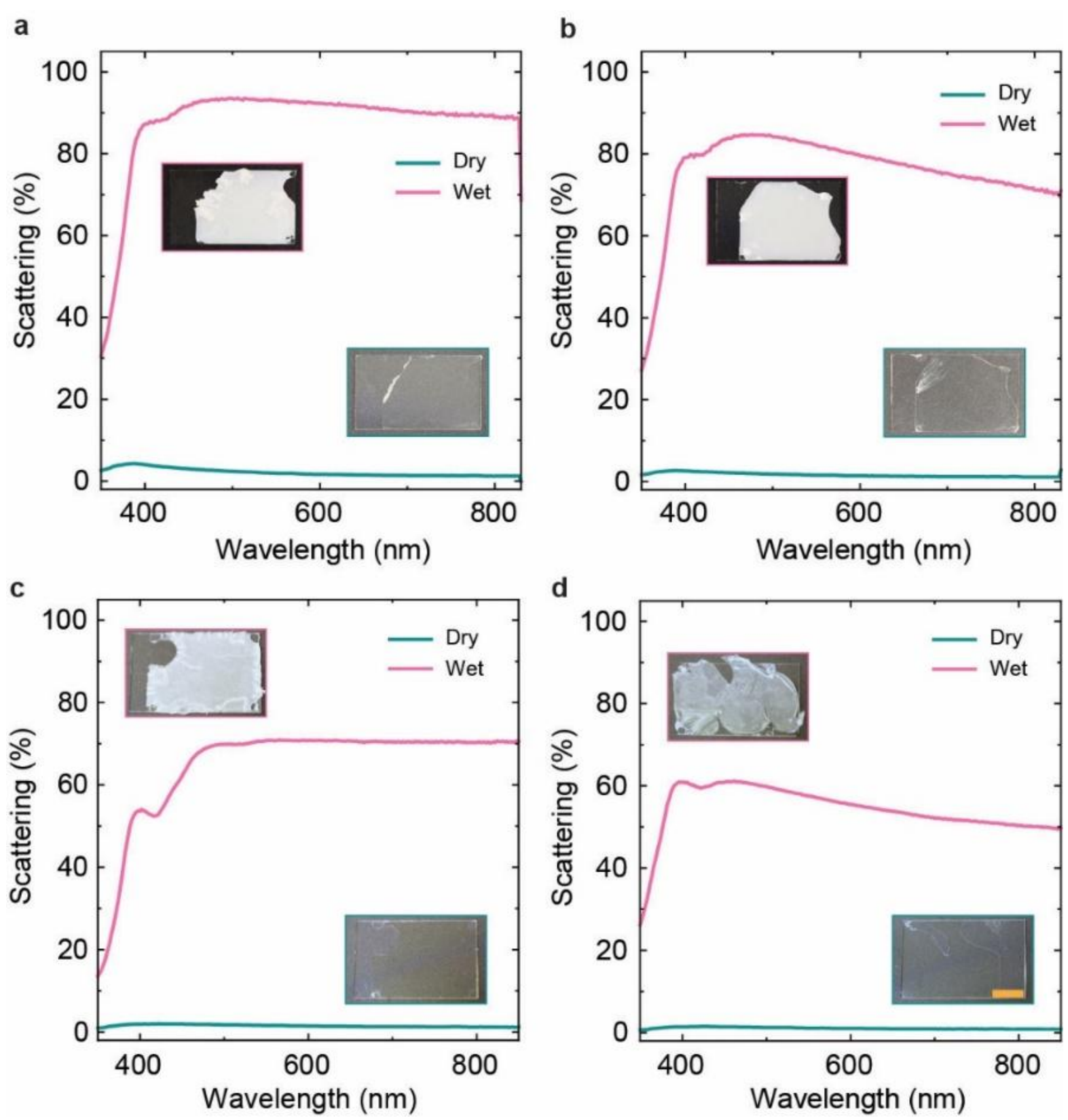

Supplementary Figure 19. Dry and wet state total scattering spectra of (a) Network 1, (b) Network 2, (c) Network 3, and (d) Network 4. Insets are the transparent and opaque photographs of respective LCNs. Film thickness: 100 µm. Scale bar: 1cm.

Supplementary Table 7: References for Fig. 3f

	Description	Reference
1	PNIPAm	1
2	Standard PNIPAm	2,3
3	PNIPAm particles	4
4	Channeled PNIPAm	2
5	Macro-crosslinked PNIPAm	5
6	PNIPAm, ethanol as a cosolvent	3
7	Hydroxypropyl cellulose	1,6
8	Hydroxypropyl cellulose in gellan gum gel	7
9	Hydroxypropyl cellulose in acrylamide gel	8

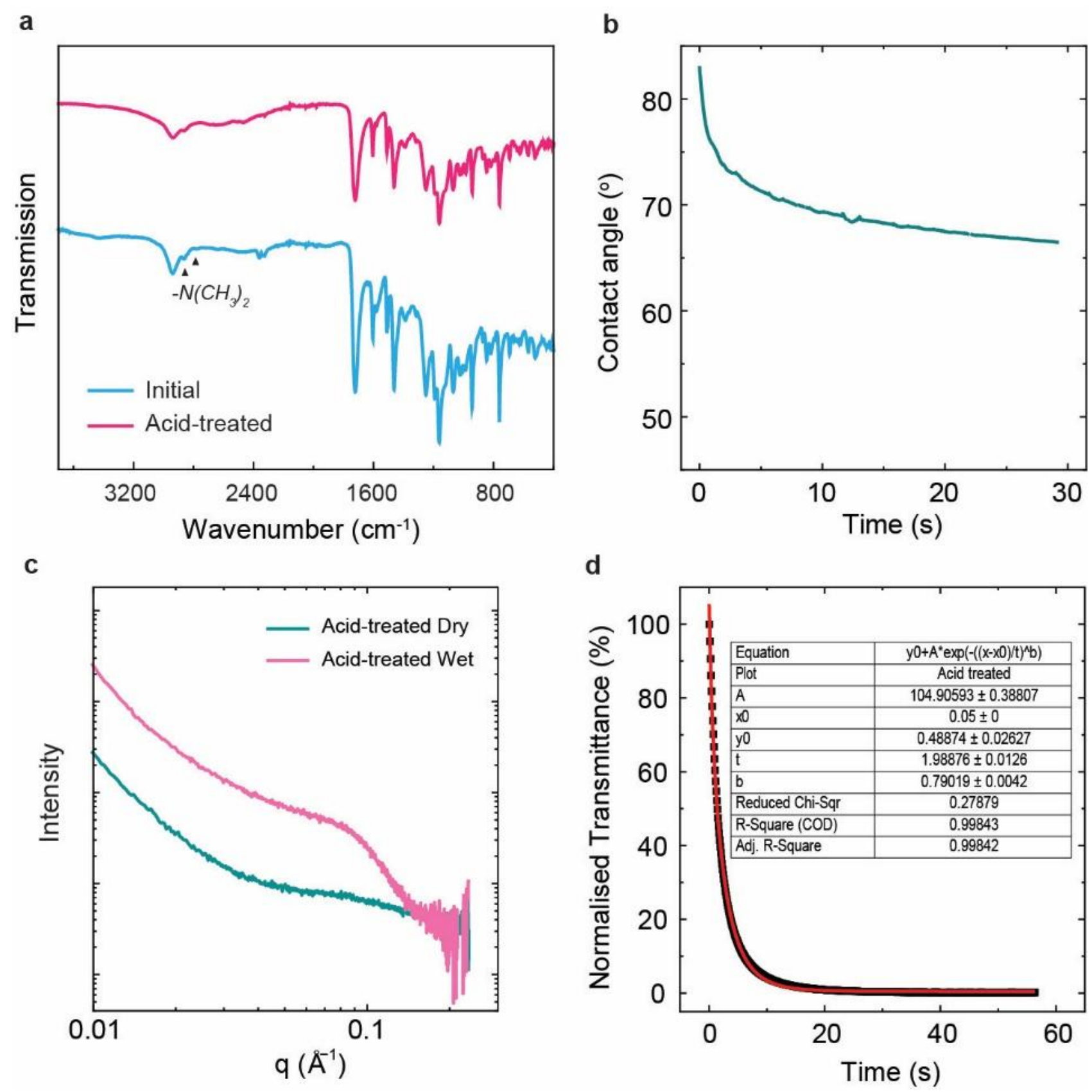

Supplementary Figure 20. Characterization of acid-treated $LCN_{AA+DMA+I}$ film (100 µm): (a) FTIR spectra of initial and HCl-treated film (0.1M, 30s), (b) Contact angle, (c) SAXS, (d) Transparent to opaque state change curve at 400 nm.

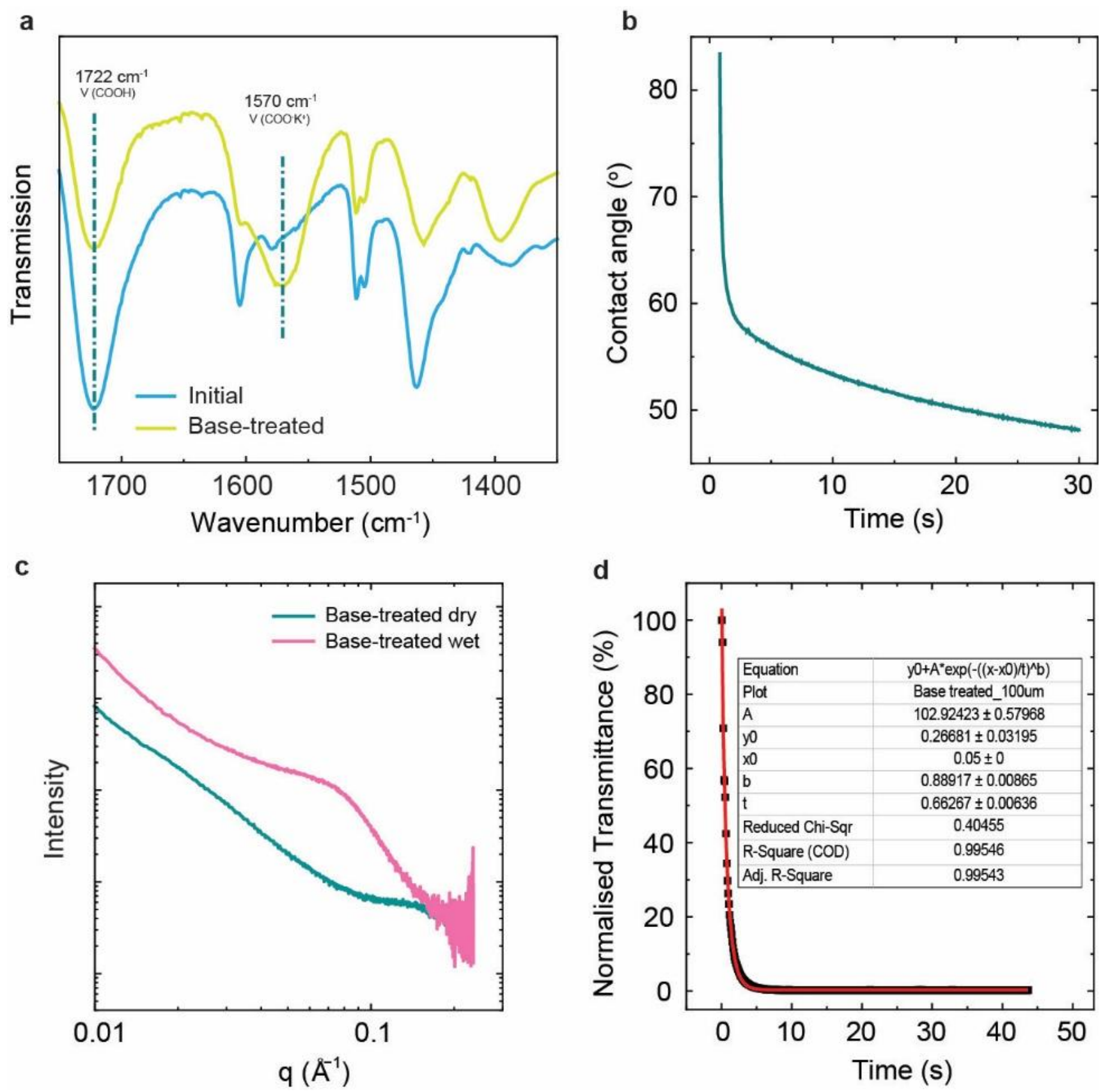

Supplementary Figure 21. Characterization of base-treated $LCN_{AA+DMA+I}$ film (100 μm): (a) FTIR spectra of initial and KOH-treated film (1M, 30s), (b) Contact angle, (c) SAXS, (d) Transparent to opaque state change curve at 400 nm.

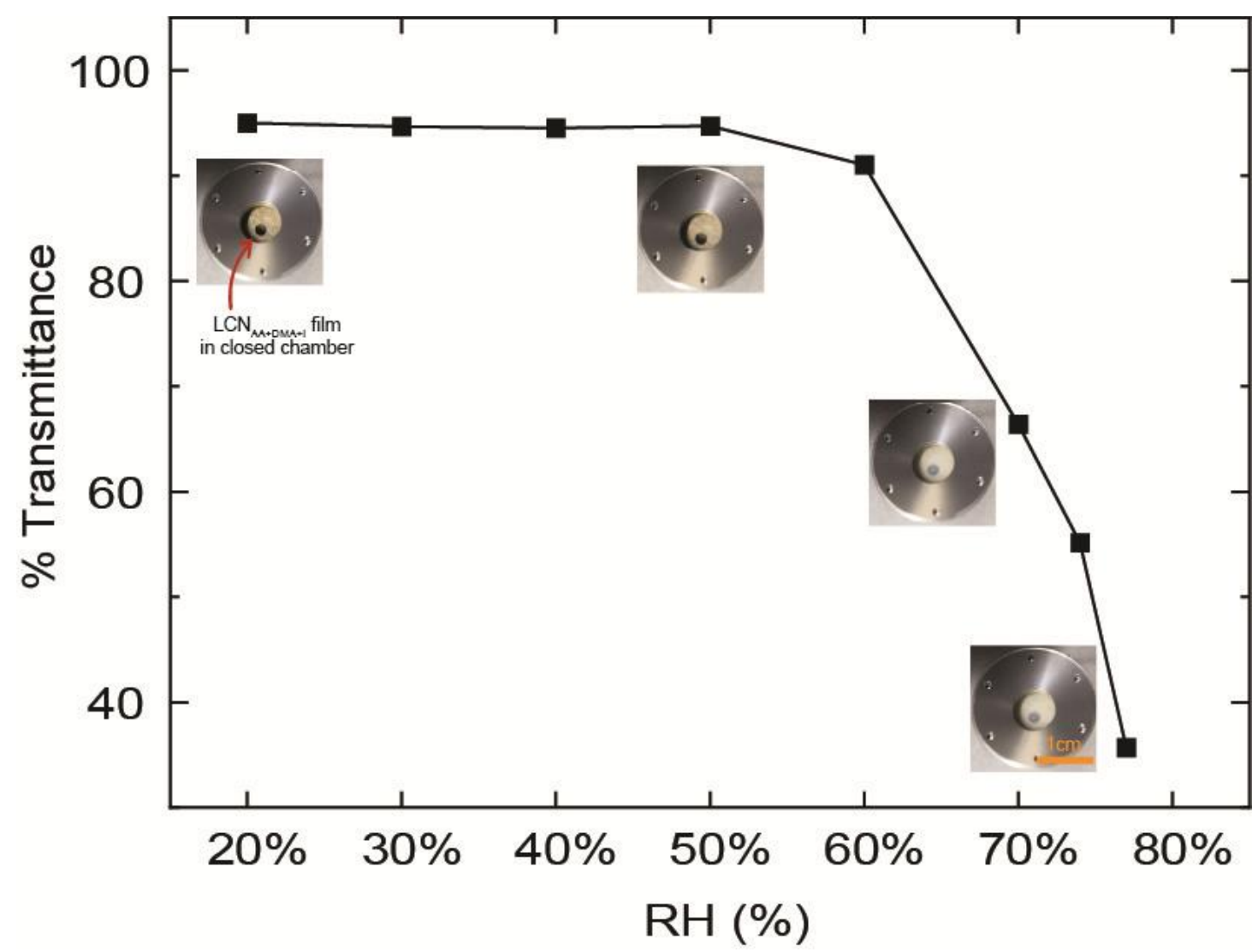

Supplementary Figure 22. Gradual decrease in transmittance of $LCN_{AA+DMA+I}$ at 450 nm with increase in RH in closed chamber at 22 ºC. Insets are corresponding pictures at respective RH. Film thickness: 100 µm.

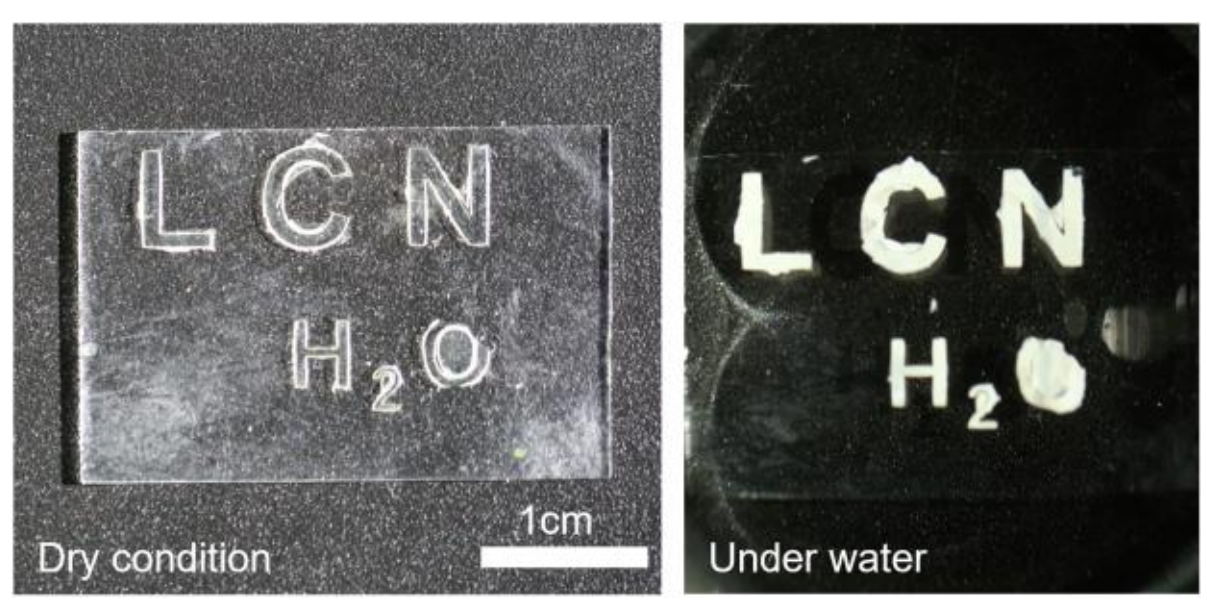

Supplementary Figure 23. Water-triggered revelation of encoded information in an $LCN_{AA+DMA+I}$ film. The laser-patterned film remains nearly transparent under dry conditions (left) but reveals the encoded "LCN H_2O" message upon immersion in water (right) due to the solvent-induced scattering transition. Film thickness: 100 µm. Scale bar: 1 cm.

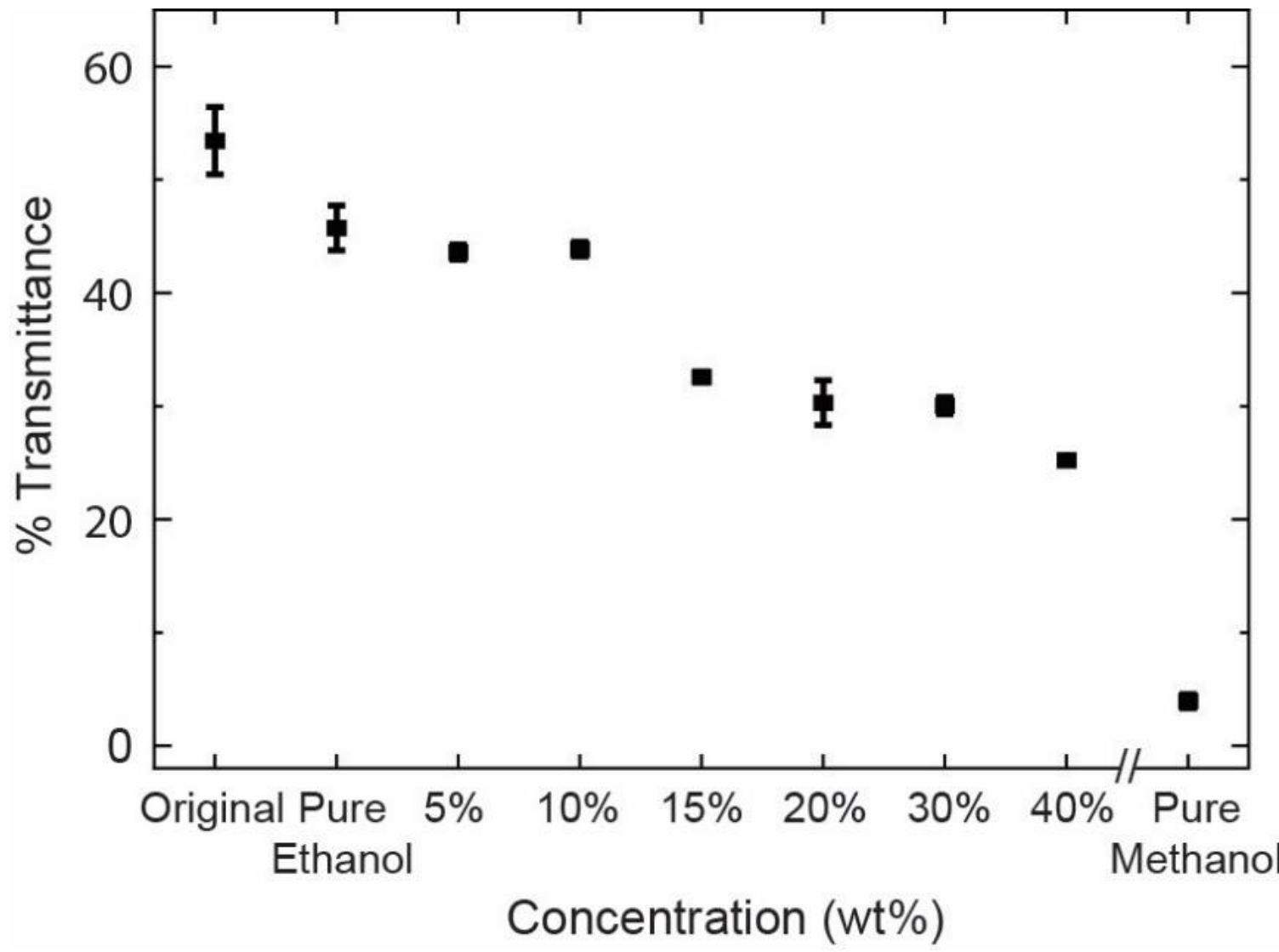

Supplementary Figure 24. Transmittance at 400 nm for methanol detection in methanol-ethanol solution mixture with $LCN_{AA+DMA+I}$ film. Error bars represent the SD obtained from n=3 measurements. Film thickness: 100 µm.

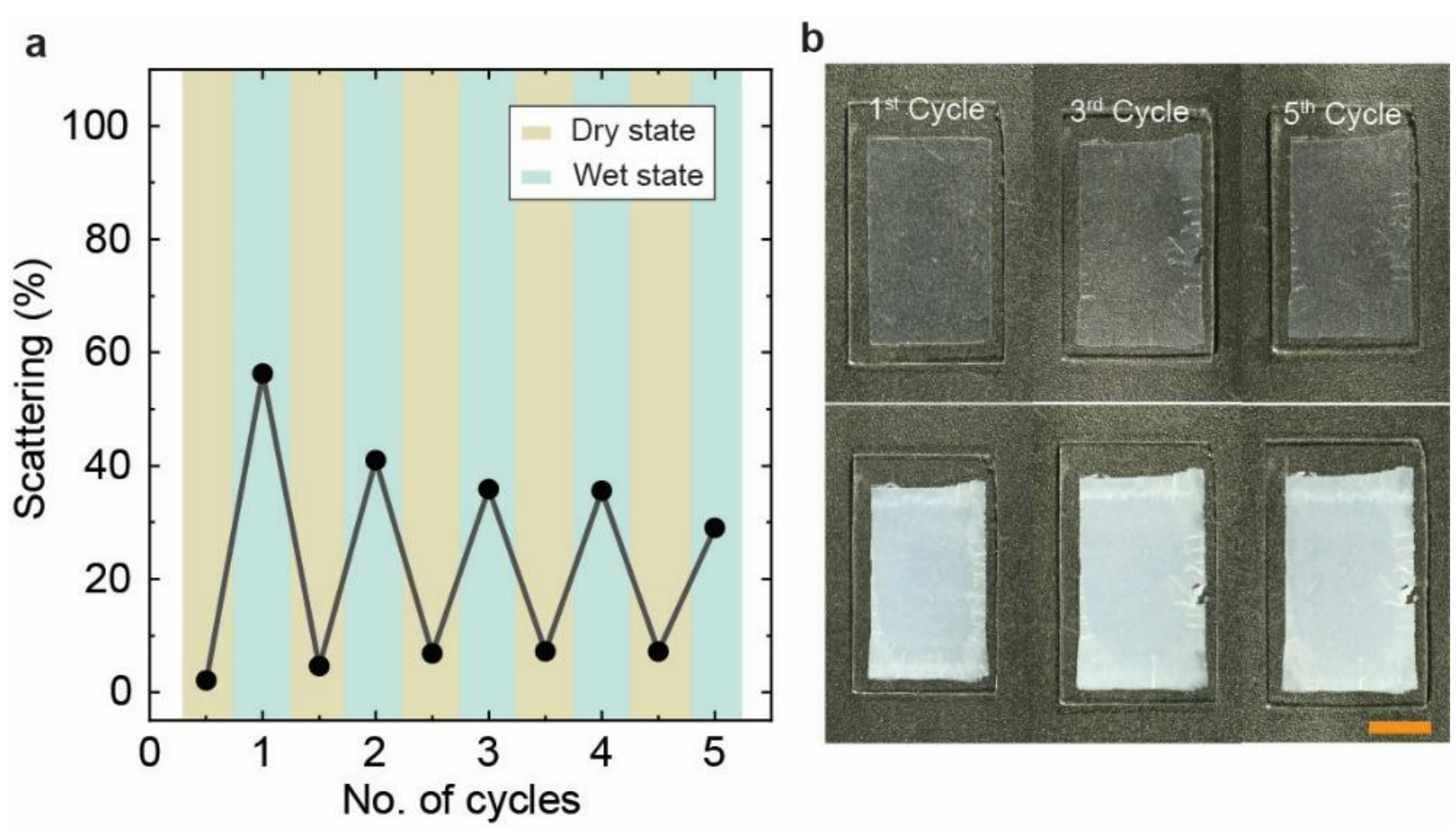

Supplementary Figure 25. (a) The reversible switching of optical scattering of $LCN_{AA+DMA+I}$ film at 500nm over 5 dry-wet cycles of 10 µm film, (b) Photographs of the same film in dry and wet state over 5 cycles. Scale bar: 1 cm

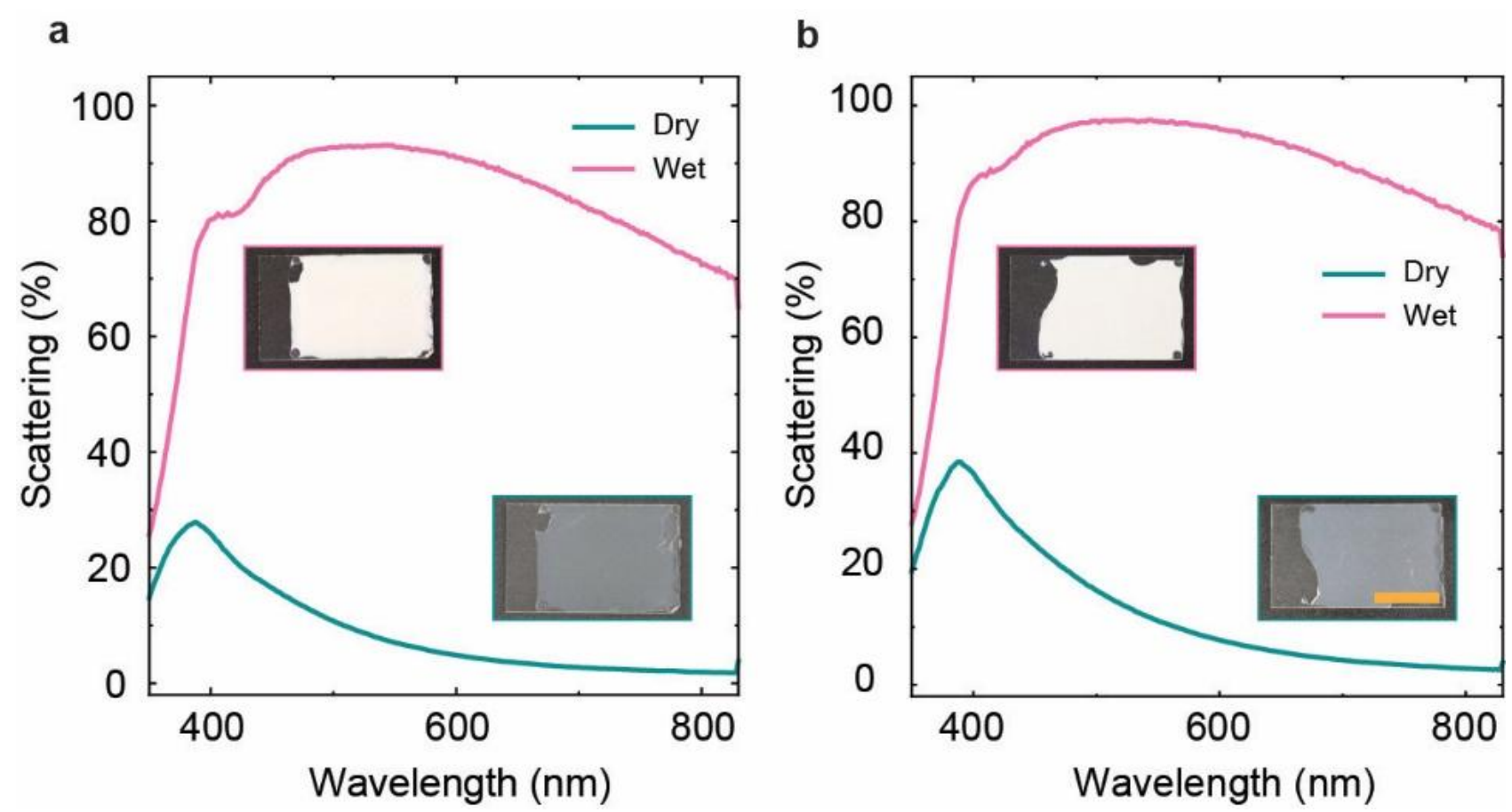

Supplementary Figure 26. Dry and wet state total scattering spectra of films where RM82 has been replaced with (a) RM257 and (b) C6BAPE, keeping other monomers and concentrations unchanged. Insets are the transparent and opaque photographs of respective LCNs in their dry and wet states. Scale bar: 1 cm. Film thickness: 100 µm.

References

1. Ming, Y., Sun, Y., Liu, X., Liu, X. & Wu, Y. Optical evaluation of a smart transparent insulation material for window application. *Energy Convers and Man- X* **16**, 100315 (2022).

2. Eklund, A., Zhang H., Zeng H., Priimagi A., Ikkala O. Fast Switching of Bright Whiteness in Channeled Hydrogel Networks. *Adv. Funct. Mater.* **30**, 2000754 (2020).

3. Ding, Y., Duan, Y., Yang, F., Xiong, Y. & Guo, S. High-transmittance pNIPAm gel smart windows with lower response temperature and stronger solar regulation. Chem. Eng. J. 460, 141572 (2023).

4. Zhou, Y. *et al.* Liquid Thermo-Responsive Smart Window Derived from Hydrogel. *Joule* **4**, 2458–2474 (2020).

5. Eklund, A. *et al.* Bright and Switchable Whiteness in Macro-Crosslinked Hydrogels. *Adv. Opt. Mater.* **12**, 2302487 (2024).

6. Connelly, K., Wu, Y., Ma, X. & Lei, Y. Transmittance and Reflectance Studies of Thermotropic Material for a Novel Building Integrated Concentrating Photovoltaic (BICPV) 'Smart Window' System. *Energies* **10**, 1889 (2017).

7. Liu, X. & Wu, Y. Experimental characterisation of a smart glazing with tuneable transparency, light scattering ability and electricity generation function. *Appl. Ene.* **303,** 117521 (2021).

8. Nakamura, A., Ogai, R. & Murakami, K. Development of smart window using an hydroxypropyl cellulose-acrylamide hydrogel and evaluation of weathering resistance and heat shielding effect. *Sol. Energy Mater. Sol. Cells* **232**, 111348 (2021).
