# Supplementary material for: Switchable whiteness in liquid crystal networks via solvent-induced microphase segregation
Source: Nat Commun. 2026 Sep 8;17:10625. doi: 10.1038/s41467-026-77442-y (PMC13649656; doi:10.1038/s41467-026-77442-y)
Supplement: Supplementary file 2 [file 41467_2026_77442_MOESM2_ESM.pdf]

Description of Additional Supplementary File

Supplementary video caption

Supplementary Movie 1. Two identical $LCN_{AA+DMA+I}$ films of thickness 100 μm are on a hot plate. One is immersed in water turn white/opaque. Further drying wet film on a hot plate at 100 ^{o}C, changes to transparent.
