# Supplementary material for: Switchable whiteness in liquid crystal networks via solvent-induced microphase segregation
Source: Nat Commun. 2026 Sep 8;17:10625. doi: 10.1038/s41467-026-77442-y (PMC13649656; doi:10.1038/s41467-026-77442-y)
Supplement: Supplementary file 3 [file 41467_2026_77442_MOESM4_ESM.pdf]

nature portfolio

Peer Review File

Switchable Whiteness in Liquid Crystal Networks via Solvent-Induced Microphase Segregation

Corresponding Author: Professor Arri Priimagi

This file contains all reviewer reports in order by version, followed by all author rebuttals in order by version.

Attachments originally included by the reviewers as part of their assessment can be found at the end of this file.

Version 0:

Reviewer comments:

Reviewer #1

(Remarks to the Author)
The authors report a liquid crystal network (LCN) that exhibits a solvent-induced scattering response. They carefully design the network to have i) liquid crystalline mesogens; ii) hydrogen bonding acrylic acid groups; iii) hydrophobic, halogen-bonded crosslinkers through dimethylaminoethyl acrylate. They carefully study the impact of network functional groups and composition on the optical scattering response and are able to describe and explain the basis for the change in scattering. They also demonstrate a number of interesting applications, such as recording a fingerprint, underwater detection, and solvent detection. This is certainly a novel contribution to the field of liquid crystal networks and elastomers that I think will be of interest to researcher in the field. The authors provide extensive characterization of their materials, and their description of methods is deetailed and adequate. I am happy to recommend acceptance with a few recommendations and questions for the authors:

1) The authors show that the sample becomes birefringent under stretching and claim this reflects liquid crystal nature. However, many polymeric materials exhibit birefringence under stretching. It is not clear that the liquid crystal mesogens used are responsible for this birefringence. More generally, are the liquid crystal groups required? I don't think it is necessary to add more experiments to the work, but I am curious as to whether any hydrophobic monomer used in place of the liquid crystal monomers would exhibit a similar scattering response.

2) The role of the DMA groups are unclear. I understand that these are additional supramolecular crosslinks that remain intact even when water is added and hydrogen bonding is disrupted. But are htese supramolecular crosslinks needed, or would replacing these with covalent crosslinks achieve the same response? The various samples shown in Supp Table 1 show that when you replace the DMA with AA then the sample remains transsparent. But what happens when DMA are removed and the equivalent number of covalent LC crosslinks are used? This might help elucidate the role of supramolecular interactions/crosslinking.

Reviewer #2

(Remarks to the Author)
[Editor's Note: See attached for Review]

Reviewer #3

(Remarks to the Author)
Nat Com

The authors introduce a liquid crystal network that reversibly switches to a highly scattering white state via solvent-induced disruption of supramolecular interactions. Polar protic solvents break hydrogen bonds, causing nanoscale phase segregation that efficiently scatters visible light without significant swelling or deformation. The response is rapid, robust, and fatigue-resistant, reaching 85% scattering efficiency in relatively thin films.
While the work is technically very good, I am a bit concerned about its novelty in context. It would be great if the author could better contextualize it, as several works of reversible witness have now been reported, for example:
https://www.nature.com/articles/s43246-025-00956-3,

https://advanced.onlinelibrary.wiley.com/doi/full/10.1002/adom.202302487
Saying that, I like the idea very much.

What is the volumetric change that is observed, as the author criticizes the hydrogel-based method in the intro? I think they should comment on this point.

While the authors claim that “The response is rapid, reversible, and requires no external stimulus, enabling simple visualization of touch patterns and suggesting potential for transient biometric marking and touch-responsive interfaces.” It would be good to have a more critical discussion on its use. Similarly, I like the spring; it is a very nice concept, but I am not sure which concept can be used. I think more than guessing would be useful for quantifying the value of polarity vs. whiteness.

Also, it would be nice to have the system's free path value. The author has already performed measurements on samples of different thicknesses.

Version 1:

Reviewer comments:

Reviewer #1

(Remarks to the Author)
The authors have sufficiently addressed comments from my prior review, and I am happy to recommend acceptance for publication.

Reviewer #2

(Remarks to the Author)
The authors have addressed the comments with additional experiments except the following ones:

Comment 2: Since mechanical durability is critical for practical applications, the authors are suggested to measure and compare the fracture toughness of the material in both states. No fracture toughness was measured.

Comment 5: additional demonstrations where this reversible optical transition offers clear advantages over existing approaches.

Reviewer #3

(Remarks to the Author)
I appreciate the response of the author and almost all my comments have been addressed, I woudl however, like to insist on reporting the values of scattering mean free path measured from the sample at different thicknesses. I think it is not out of the scope, as it is pertinent to a more rigorous description of the scattering properties of the material produced. Plotting the Transmission vs thickness and showing that the scales are properly scaled also demonstrates that the samples with different thicknesses have the same morphology.

Version 2:

Reviewer comments:

Reviewer #2

(Remarks to the Author)
The reviewer has addressed all the comments.

Reviewer #3

(Remarks to the Author)
I am pleased that the authors have addressed my previous comment and reported measurements for samples of different thicknesses. It would further strengthen the manuscript if the authors could also report the value of the scattering mean free path obtained in the main text, as I was unable to locate it. Additionally, a comparison with scattering mean free path values reported for other scattering materials in the literature would be very valuable, as it would provide readers with a clear sense of the actual scattering performance of the material under study.

Open Access This Peer Review File is licensed under a Creative Commons Attribution 4.0 International License, which permits use, sharing, adaptation, distribution and reproduction in any medium or format, as long as you give appropriate credit to the original author(s) and the source, provide a link to the Creative Commons license, and indicate if changes were made.
In cases where reviewers are anonymous, credit should be given to 'Anonymous Referee' and the source.
The images or other third party material in this Peer Review File are included in the article's Creative Commons license, unless indicated otherwise in a credit line to the material. If material is not included in the article's Creative Commons license and your intended use is not permitted by statutory regulation or exceeds the permitted use, you will need to obtain permission directly from the copyright holder.
To view a copy of this license, visit https://creativecommons.org/licenses/by/4.0/

Re: Switchable Whiteness in Liquid Crystal Networks via Solvent-Induced Microphase Segregation (manuscript NCOMMS-25-105727), by R. Nasare, et al.

Point by Point Response to Reviewer Comments

Reviewer #1: *The authors report a liquid crystal network (LCN) that exhibits a solvent-induced scattering response. They carefully design the network to have i) liquid crystalline mesogens; ii) hydrogen bonding acrylic acid groups; iii) hydrophobic, halogen-bonded crosslinkers through dimethylaminoethyl acrylate. They carefully study the impact of network functional groups and composition on the optical scattering response and are able to describe and explain the basis for the change in scattering. They also demonstrate a number of interesting applications, such as recording a fingerprint, underwater detection, and solvent detection. This is certainly a novel contribution to the field of liquid crystal networks and elastomers that I think will be of interest to researcher in the field. The authors provide extensive characterization of their materials, and their description of methods is deetailed and adequate. I am happy to recommend acceptance with a few recommendations and questions for the authors:*

Our answer: We are grateful for the reviewer's positive comments. Please see below our point-by-point response to the reviewer's concerns.

1) The authors show that the sample becomes birefringent under stretching and claim this reflects liquid crystal nature. However, many polymeric materials exhibit birefringence under stretching. It is not clear that the liquid crystal mesogens used are responsible for this birefringence. More generally, are the liquid crystal groups required? I don't think it is necessary to add more experiments to the work, but I am curious as to whether any hydrophobic monomer used in place of the liquid crystal monomers would exhibit a similar scattering response.

Our answer: We thank the reviewer for this insightful comment regarding the origin of birefringence and the role of liquid crystal (LC) mesogens in the present system.

First, we agree that birefringence under mechanical stretching is not unique to liquid crystalline materials, as many polymer networks can exhibit birefringence due to polymer chain orientation. However, in our system, the observed birefringence clearly arises from the alignment of liquid crystal mesogens, as confirmed by control experiments. We prepared new polymer networks in which the LC mesogens were replaced with non-LC monomers (Supplementary Table 6): RM82 was replaced with bisphenol A polyethylene glycol diether diacrylate (m+n ≈ 10), or 1,4-phenylene diacrylate, and RM105 was replaced with phenyl acrylate. These polymers, lacking liquid crystal units, showed no bright–dark contrast when imaged with polarized optical microscope (Network 2, Supplementary Figure 2).

Supplementary Table 6. Compositions (in mmol) of the non-liquid-crystalline reference polymer networks

	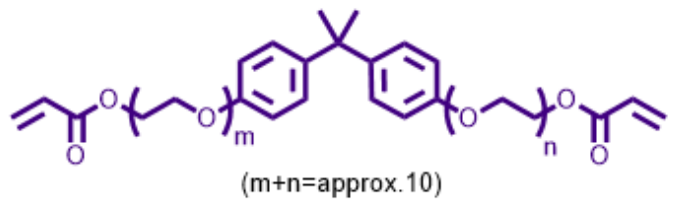	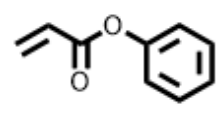	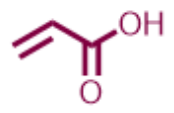	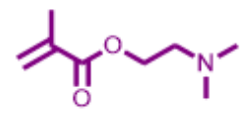	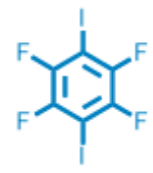
Network 1	3	-	100	50	25
Network 2	3	15	100	50	25
	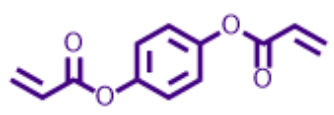	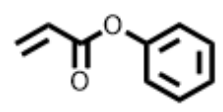	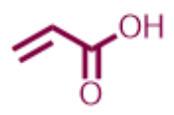	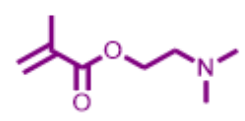	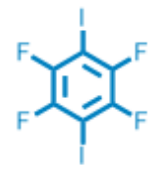
Network 3	3	-	100	50	25
Network 4	3	15	100	50	25

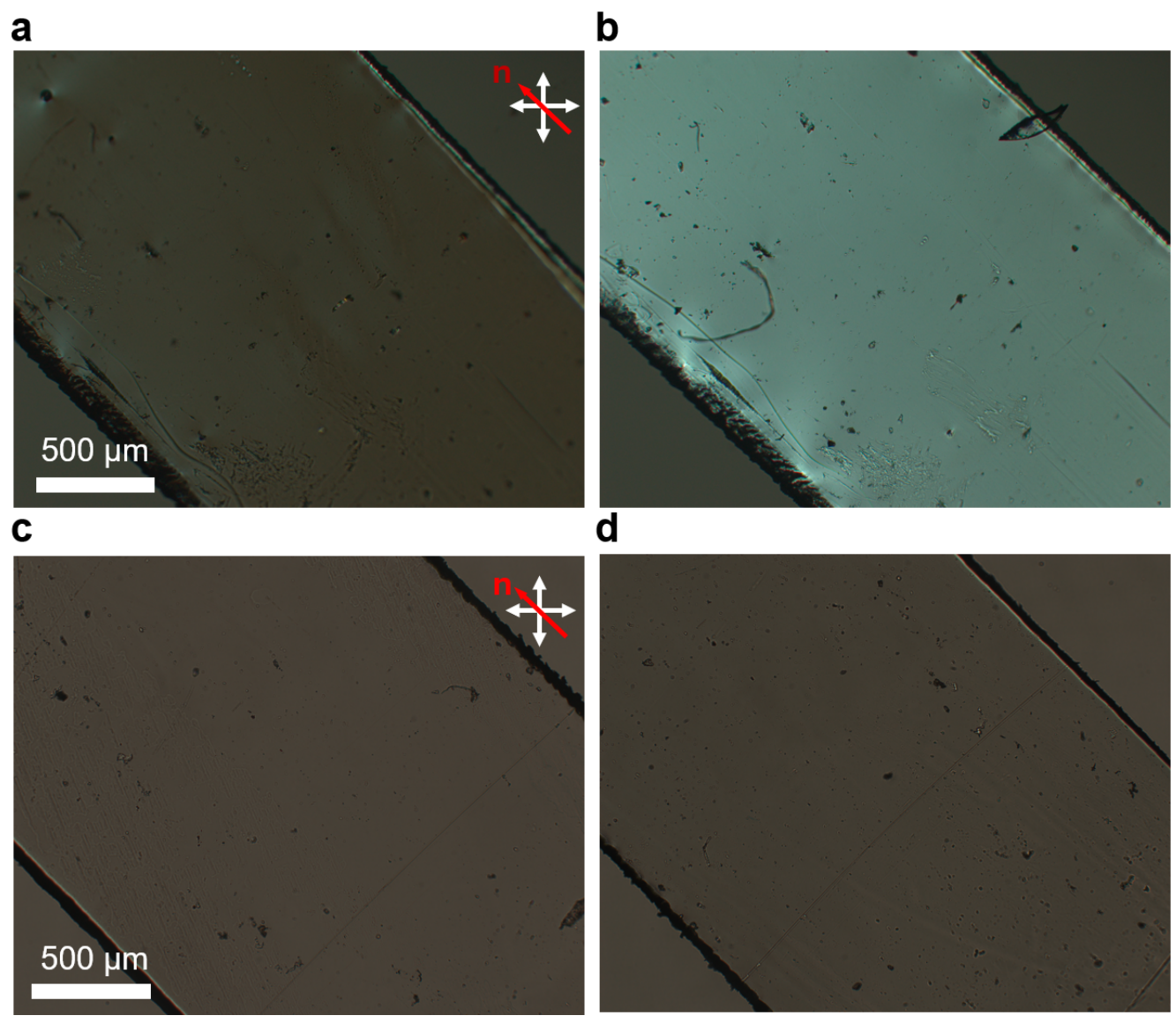

Supplementary Figure 2. Polarized optical micrographs of $LCN_{AA+DMA+I}$ film (a) before, and (b) after 5-10 % stretching, and for Network 2 (c) before, and (d) after 5-10% stretching. Film thicknesses: 100 µm.

To clarify whether LC mesogens are required for the observed whitening response, we measured the scattering of Networks 1-4 in dry and wet states (Supplementary Fig. 17). These control samples remain highly transparent in the dry state, and upon immersion in water, turn white, like the LC networks. This indicates that the reversible optical scattering does not rely on liquid crystalline ordering, although liquid crystallinity influences the mechanical and structural properties of the network. Hence we conclude that the key mechanism responsible for the reversible whitening originates from the disruption of supramolecular HB by polar protic solvents, which induces nanoscale phase segregation and efficient light scattering.

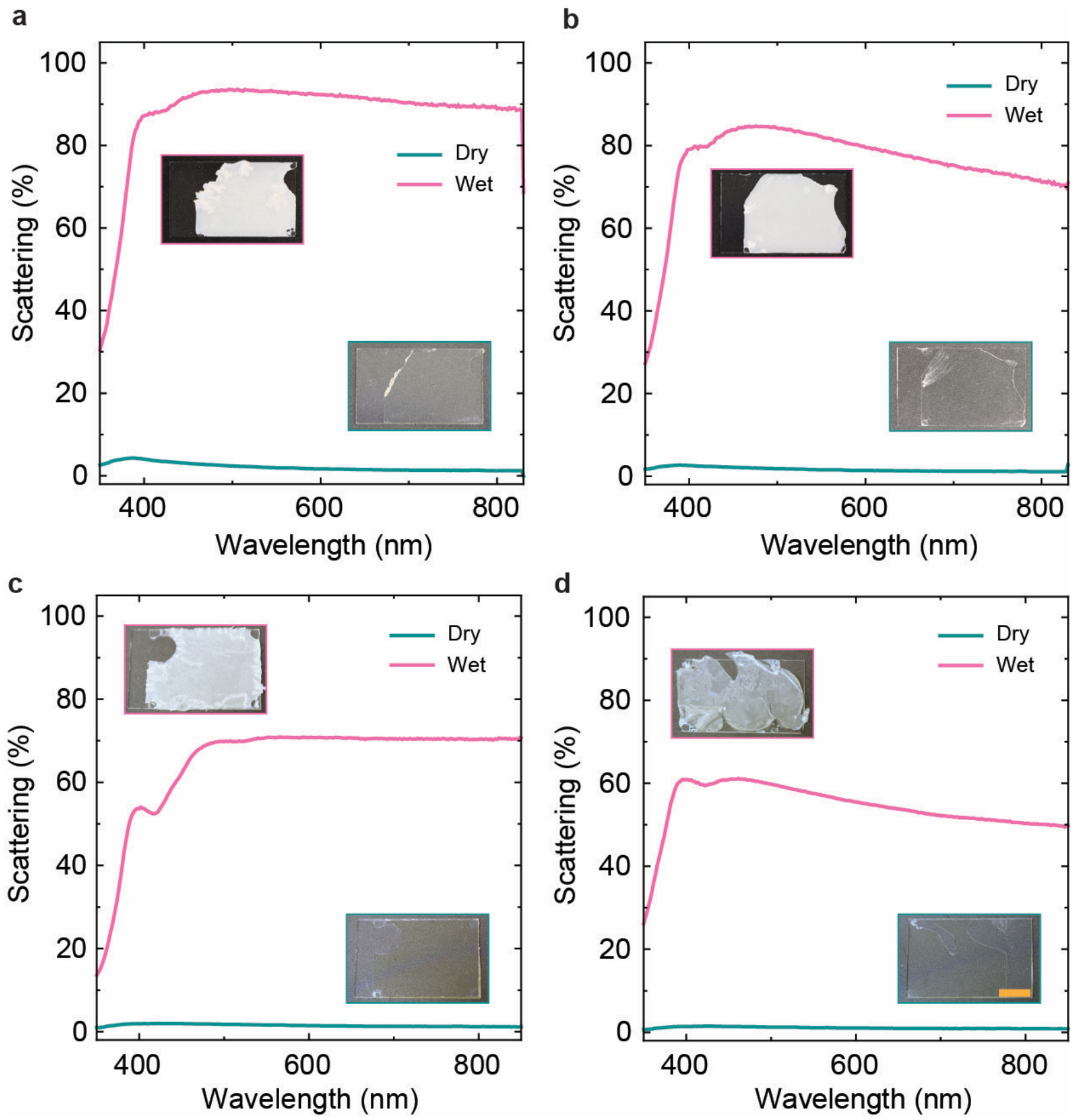

Supplementary Figure 17. Dry and wet state total scattering spectra of (a) Network 1, (b) Network 2, (c) Network 3, and (d) Network 4. Insets are the transparent and opaque photographs of respective LCNs. Film thickness: 100 µm. Scale bar: 1cm.

To address this comment, the above-described control experiments and the corresponding discussion have now been added to the revised manuscript (Supplementary Table 6, Supplementary Figs. 2&17). The modified text reads as follows (p. 13):

To clarify the role of liquid crystal (LC) mesogens, we performed control experiments in which the LC units were replaced with non-LC monomers: RM82 was replaced with bisphenol A polyethylene glycol diether diacrylate (m+n ≈ 10) or 1,4-phenylene diacrylate, and RM105 with phenyl acrylate (Supplementary Table 6). These polymers, lacking LC units, exhibited no bright–dark contrast when stretched and imaged with polarized optical microscope (Network 2, Supplementary Figure 2c&d). They did, however, display pronounced and reversible whitening response upon exposure to water (Supplementary Fig. 17). These results indicate that the reversible optical scattering primarily originates from disruption of supramolecular HB and nanoscale phase segregation. LC mesogens, while influencing mechanical and structural properties, are therefore not essential for the whitening behavior.

2) The role of the DMA groups are unclear. I understand that these are additional supramolecular crosslinks that remain intact even when water is added and hydrogen bonding is disrupted. But are these supramolecular crosslinks needed, or would replacing these with covalent crosslinks achieve the same response? The various samples shown in Supp Table 1 show that when you replace the DMA with AA then the sample remains transsparent. But what happens when DMA are removed and the equivalent number of covalent LC crosslinks are used? This might help elucidate the role of supramolecular interactions/crosslinking.

Our answer: We thank the reviewer for this insightful comment. To clarify the role of the DMA groups, we performed control experiments in which the DMA units were replaced with an equivalent number of LC monomers RM82, and RM105 (Supporting Table 4, LCN CT 5). In these samples, there was no whitening upon immersion in water, indicating that simply replacing DMA with LC monomers cannot reproduce the effect. In our system, DMA primarily serves as a halogen-bond-accepting unit. Even in the absence of a halogen-bond donor, light scattering occurs in the wet state due to residual phase separation, but the contrast is weaker. Therefore, the presence of DMA is essential for achieving a pronounced, reversible whitening response, as its reversible supramolecular interactions, including HB and XB, enable nanoscale phase separation and efficient light scattering that cannot be realized in purely covalent networks. Additionally, as shown in LCN CT 6 and LCN CT 7 (Supplementary Table 4 and Supplementary Fig. 15), replacing the DMA (tertiary amine) with an equivalent hydrophobic co-monomer (butyl methacrylate) or with secondary amine (N-isopropylacrylamide) eliminates the whitening behavior confirming essential role of tertiary amine in switchable whiteness control.

Supplementary Table 4. Different compositions used in mmol.

	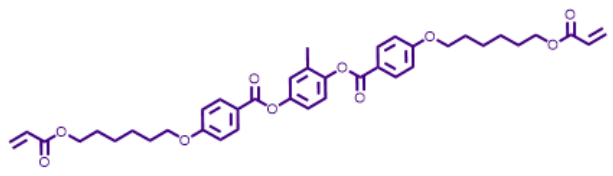	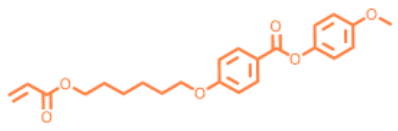	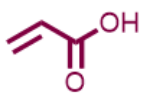	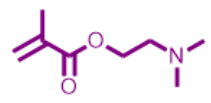	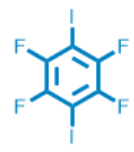
LCN CT 1	3	-	100	50	25
CT 2	-	15	100	50	25
CT 3	-	-	100	50	25
CT 4	-	-	100	50	-
LCN CT 5	13	55	100	-	25
	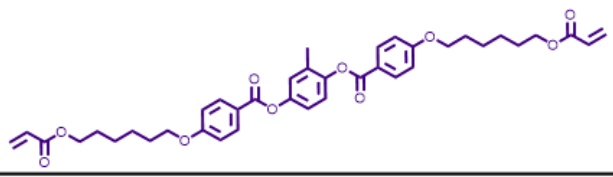	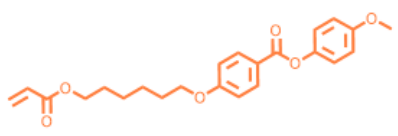	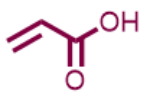	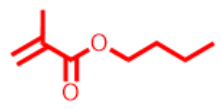	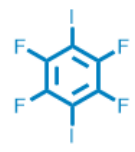
LCN CT 6	3	15	100	50	25
	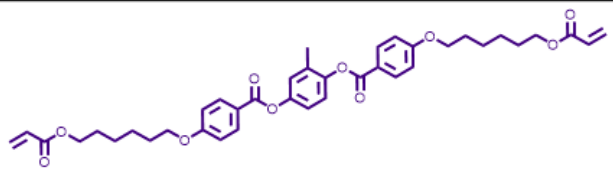	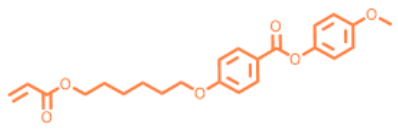	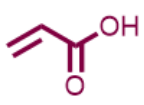	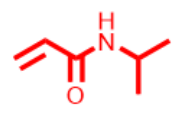	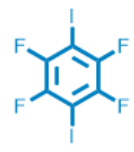
LCN CT 7	3	15	100	50	25

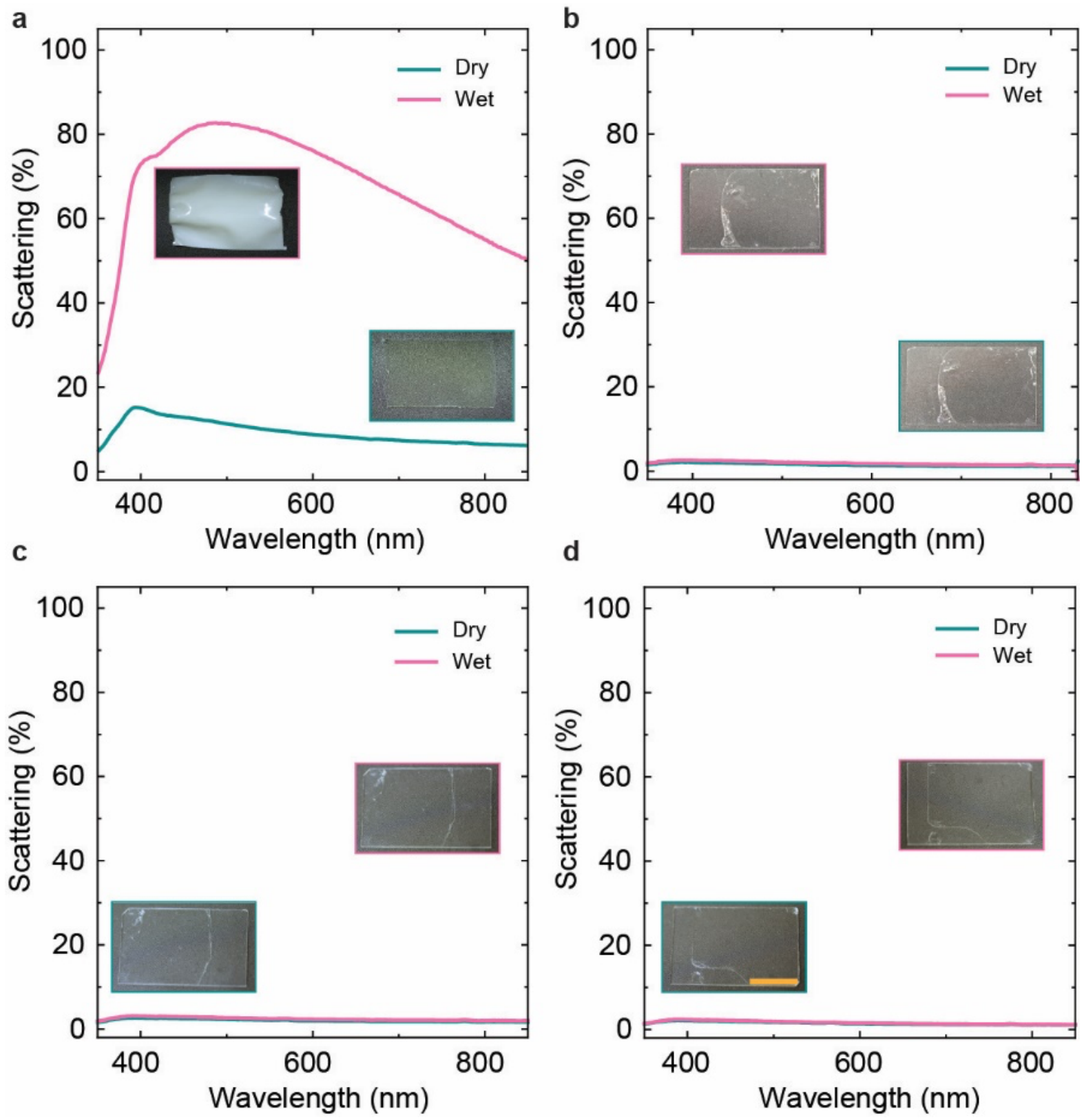

Supplementary Figure 15. Total scattering spectra of (a) LCN CT 1, (b) LCN CT 5, (c) LCN CT 6, (d) LCN CT 7. Insets are the transparent and opaque photographs of respective LCNs. Thickness: 100 µm. Scale bar: 1 cm.

To address this comment, the above-described control experiments and the corresponding discussion have now been added to the revised manuscript (Supplementary Table 4, Supplementary Fig. 15). The modified text reads as follows (p. 12):

The content of the XB donor also affected the scattering properties (Supplementary Table 3). In the wet state, all samples exhibited comparable scattering intensities, suggesting that donor concentration has only a minor effect on maximum whiteness (Fig. 3d, full spectra at Supplementary Fig. 14). In contrast, in the dry state, scattering increased markedly in the absence of donors. These findings demonstrate that while donor concentration does not directly control the absolute whitening efficiency, it modulates the switching contrast by suppressing dry-state scattering. Interestingly, replacing the tertiary amine (DMA) with an equivalent amount of LC monomers (RM82 & RM105, LCN CT 5), a hydrophobic co-monomer (butyl methacrylate; LCN CT 6), or a secondary amine (N-isopropylacrylamide; LCN CT 7) eliminates the whitening response entirely. These results indicate that DMA-mediated supramolecular interactions are essential for the observed reversible whitening (Supplementary Table 4, and Supplementary Fig. 15b-d).

Reviewer 2*: The authors report a liquid crystal network (LCN) that exhibits reversible whitening iduced by solvent-triggered disruption of supramolecular interactions. Exposure to polar protic solvents disrupts hydrogen bonding and leads to nanoscale phase segregation, forming domains that scatter visible light without inducing significant macroscopic swelling or mechanical deformation. While the underlying mechanism is described and several brief demonstrations are provided, the broader significance and practical impact of this reversible whitening behavior remain insufficiently justified. In particular, the reviewer has the following questions.*

Our answer: We thank the reviewer for the careful reading of our manuscript and for the constructive comments. We appreciate the reviewer's summary of our work and the insightful questions raised. All the points have been carefully considered, and the manuscript has been revised accordingly. Detailed responses are provided below.

1. The rationale for selecting RM82 and RM105 as the mesogenic building blocks is not sufficiently explained. It would be helpful if the authors could clarify why these specific mesogens were chosen and whether the proposed reversible whitening mechanism is generalizable to other commonly used mesogens (e.g., RM257).

Our answer: The phenomenon reported is not specific to RM82 and RM105, which were chosen merely due to their wide utility in LCN research. We selected RM82 because of its relatively flexible spacers that help provide a stable yet flexible polymer network when used as a crosslinker. RM105 was incorporated mainly to tune the hydrophilic–hydrophobic balance of the network and provide flexibility in the polymer by decreasing crosslinking density. It can be replaced by other monomers, although such substitutions influence the mechanical properties and network structure. Therefore, RM82 and RM105 were chosen here as representative mesogenic components to construct a stable liquid crystal network.

Importantly, the reversible whitening behavior does not depend on the specific chemical identity of these mesogens but other commonly used mesogens can also be used. The dynamic whitening arises from solvent-induced dissociation of HB interactions, which leads to nanoscale phase segregation and light scattering within the network. Because this mechanism relies on supramolecular interactions rather than a particular mesogen structure, it is expected to be general for other mesogenic systems capable of forming similar dynamic networks.

To support this point, we performed control experiments using other liquid crystal monomers, where RM82 was replaced with RM257 (LCN_{RM257}), and C6BAPE (LCN_{C6BAPE}). The new LCNs still exhibit reversible whitening behavior (Supplementary Fig. 22) while changing the mesogenic diacrylate does affect the mechanical properties of the network (Supporting Fig. 1): RM82 makes the network more elastic, providing films that are easier to handle.

To address this comment, the following modifications have been made to the revised manuscript (p. 18):

The mechanical robustness of these LCN films markedly exceeds that of previously reported hydrogels exhibiting switchable whiteness, [44] with a breaking stress of *ca.* 27 MPa for 100 μm thick

LCN films *vs. ca.* 4.2 MPa for 1000 µm thick hydrogels. Remarkably, even films as thin as 10 µm exhibit up to 43% visible-light scattering in the wet state while maintaining excellent reversibility and showing no signs of mechanical fatigue over repeated wetting–drying cycles (Supplementary Fig. 16). The reflectance contrast reaches 40 % at 500 nm (Fig. 3f) and can be further increased to nearly 50% through simple post-treatment with acid or base. Unlike hydrogel-based dynamic scattering materials that often rely on macroscopic deformation or thermal activation, our LCN provides rapid, robust, and fatigue-resistant optical switching via solvent-triggered nanoscale phase segregation. Furthermore, the scattering efficiency and response kinetics can be systematically tuned through molecular design, incorporation of XB dopants, and post-synthetic modification.

The observed whitening behavior is not limited to the specific mesogenic components employed here. RM82 and RM105 were selected as representative examples providing network stability and flexibility. When replacing RM82 with common mesogenic crosslinkers RM257 or C6BAPE, the dynamic whitening response is preserved (Supplementary Fig. 22). In fact, even non-liquid-crystalline building blocks (Networks 1-4, Supplementary Table 6) can be used, although in some cases at the expense of the mechanical properties of the network. These results indicate that the whitening transition is governed primarily by solvent-induced disruption of the hydrogen-bonded network, leading to nanoscale phase segregation and light scattering, rather than by the specific chemical identity of the mesogens, highlighting the generality of the underlying mechanism and establishing a broadly applicable design principle for dynamic scattering materials.

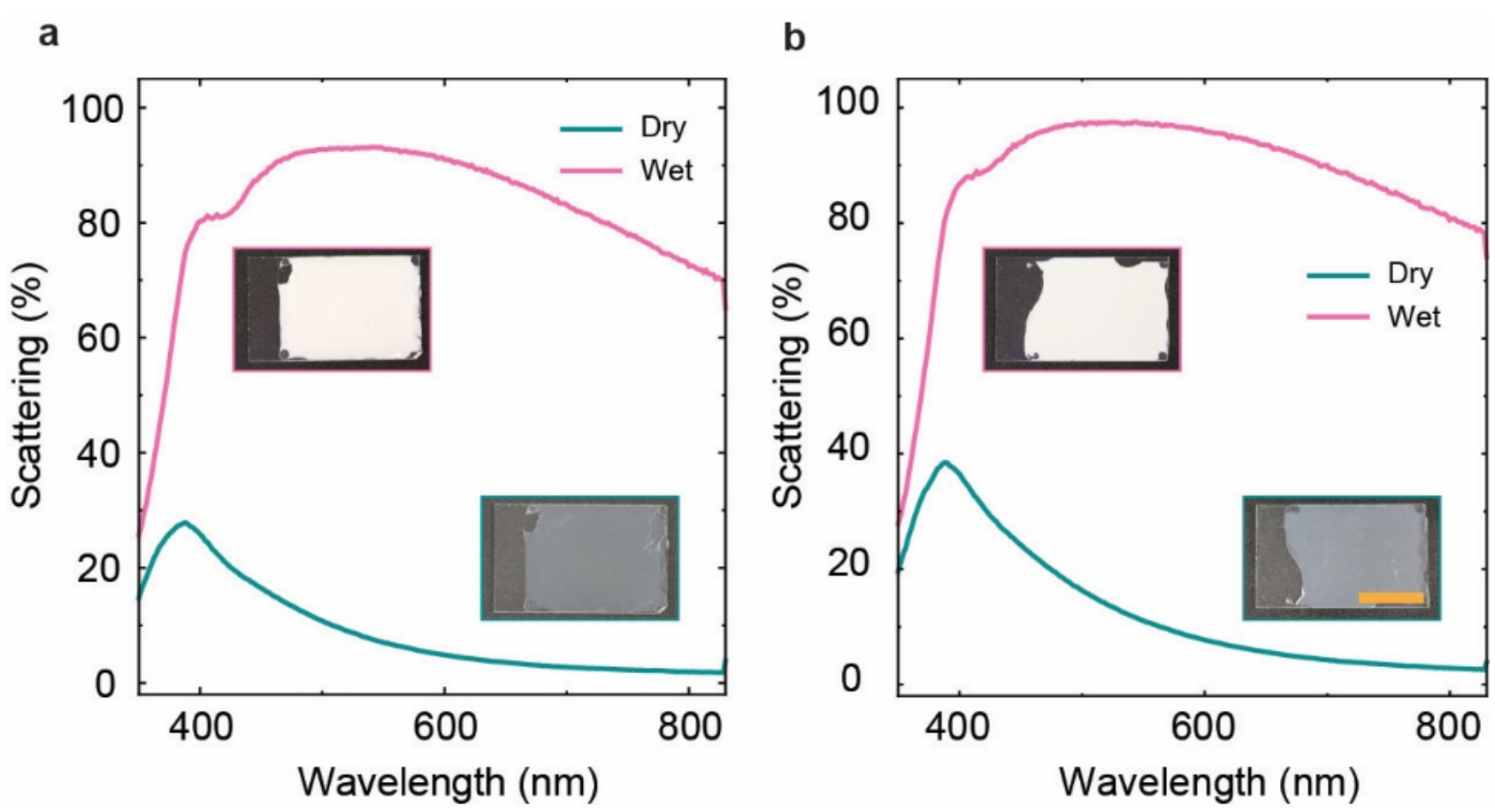

Supplementary Figure 22. Dry and wet state total scattering spectra of films where RM82 has been replaced with (a) RM257 and (b) C6BAPE, keeping other monomers and their concentrations unchanged. Insets are the transparent and opaque photographs of respective LCNs in their dry and wet states. Scale bar: 1 cm. Film thickness: 100 µm.

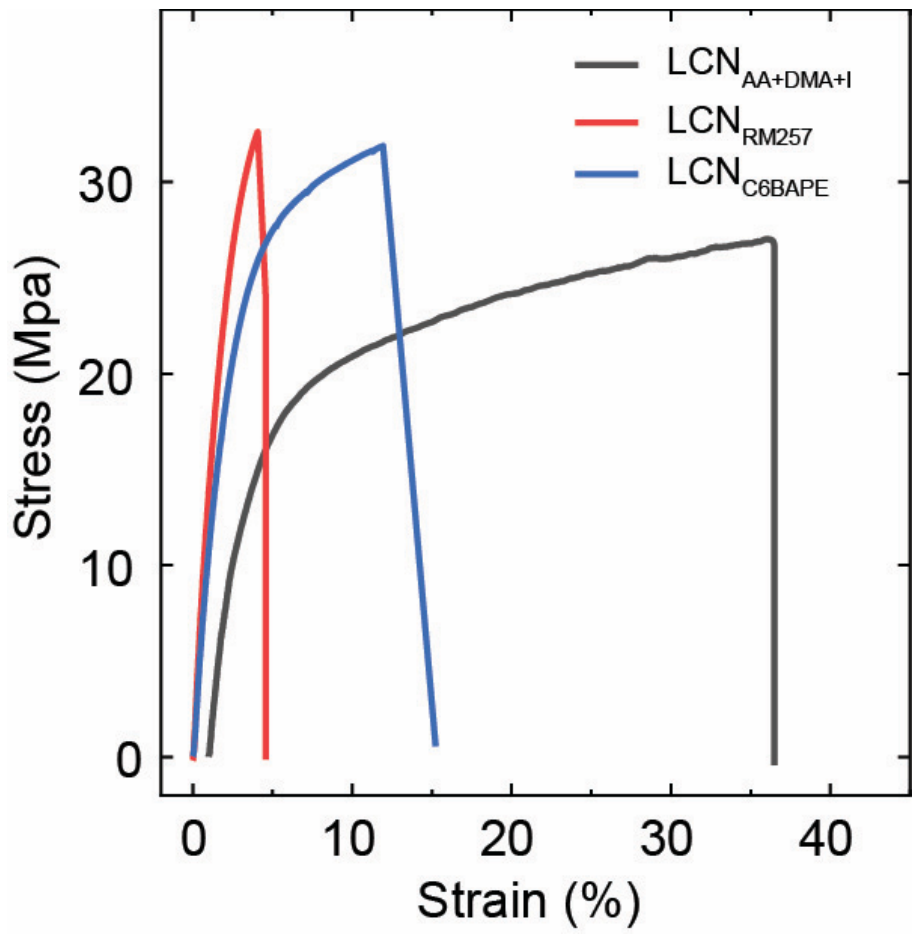

Supporting Figure 1 (for review purposes only). Tensile stress–strain curves of $LCN_{AA+DMA+I}$, LCN_{RM257}, and LCN_{C6BAPE} in the dry state. Film thickness: 100 µm.

2. In Figure 2h, repeatability is demonstrated over five dry–wet cycles. A larger number of cycles (e.g., 30–50 cycles) would be necessary to convincingly establish the robustness and long-term stability of the reversible whitening behavior.

Our answer: We thank the reviewer for the suggestion. Following your comment, we have performed additional dry–wet cycling tests up to 30 cycles. The results show that the reversible whitening behavior remains stable and fully repeatable over all cycles, confirming the robustness and long-term stability of the system. These new data have been updated to figure 2h. The following text have been added to revised manuscript (p. 10):

The durability of $LCN_{AA+DMA+I}$ film was tested over repeated wetting and drying cycles, and the film shows reversible whiteness switching with no fatigue over 30 switching cycles (Fig. 2h&i).

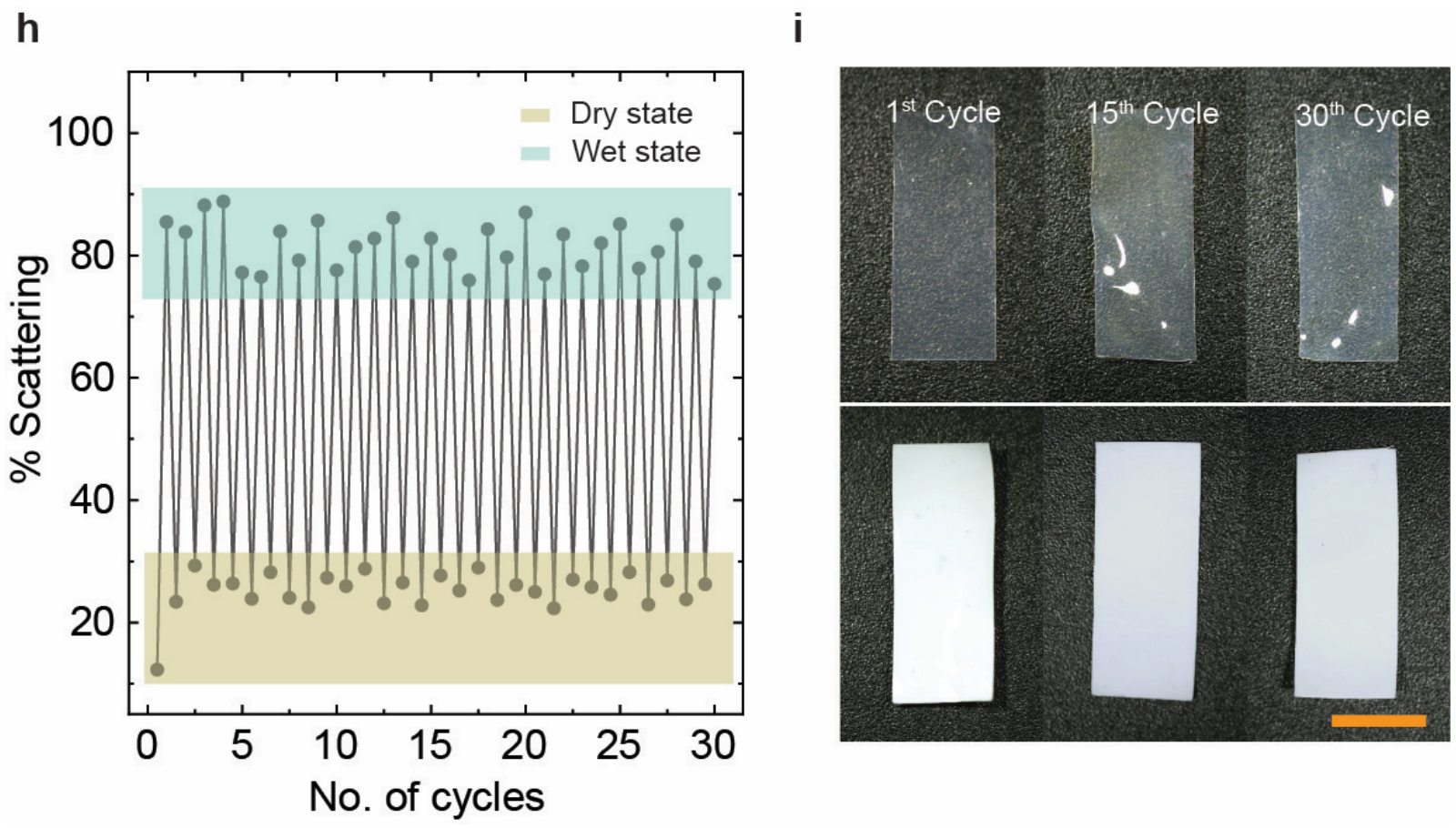

Fig. 2h Reversible switching of optical scattering of $LCN_{AA+DMA+I}$ film at 500 nm over 30 dry–wet cycles, demonstrating reversibility. **i** Photographs of the same film over the dry–wet cycles. Scale bar: 1 cm. Film thickness: 100 µm.

3. Mechanical testing shows a reduction in modulus and breaking stress in the white state compared to the transparent state. Since mechanical durability is critical for practical applications, the authors are suggested to measure and compare the fracture toughness of the material in both states.

Our answer: We performed additional fracture toughness measurements by making artificial fracture for the $LCN_{AA+DMA+I}$ film in both the transparent (dry) and the white (wet) states, and studied their mechanical properties (Supporting Fig. 2)

The results show the decrease in breaking stress and strain from 27 MPa to 21 MPa and 28% to 4% in transparent state, and from 4MPa to 0.7 MPa and 31% to 11% in white state. This behavior can be attributed to the fact that the fracture creates a defect in the material, reducing its stretchability and mechanical resistance. But even with a fracture, the film maintains its reversible whitening behavior and can still be reused within the breaking limit for various applications.

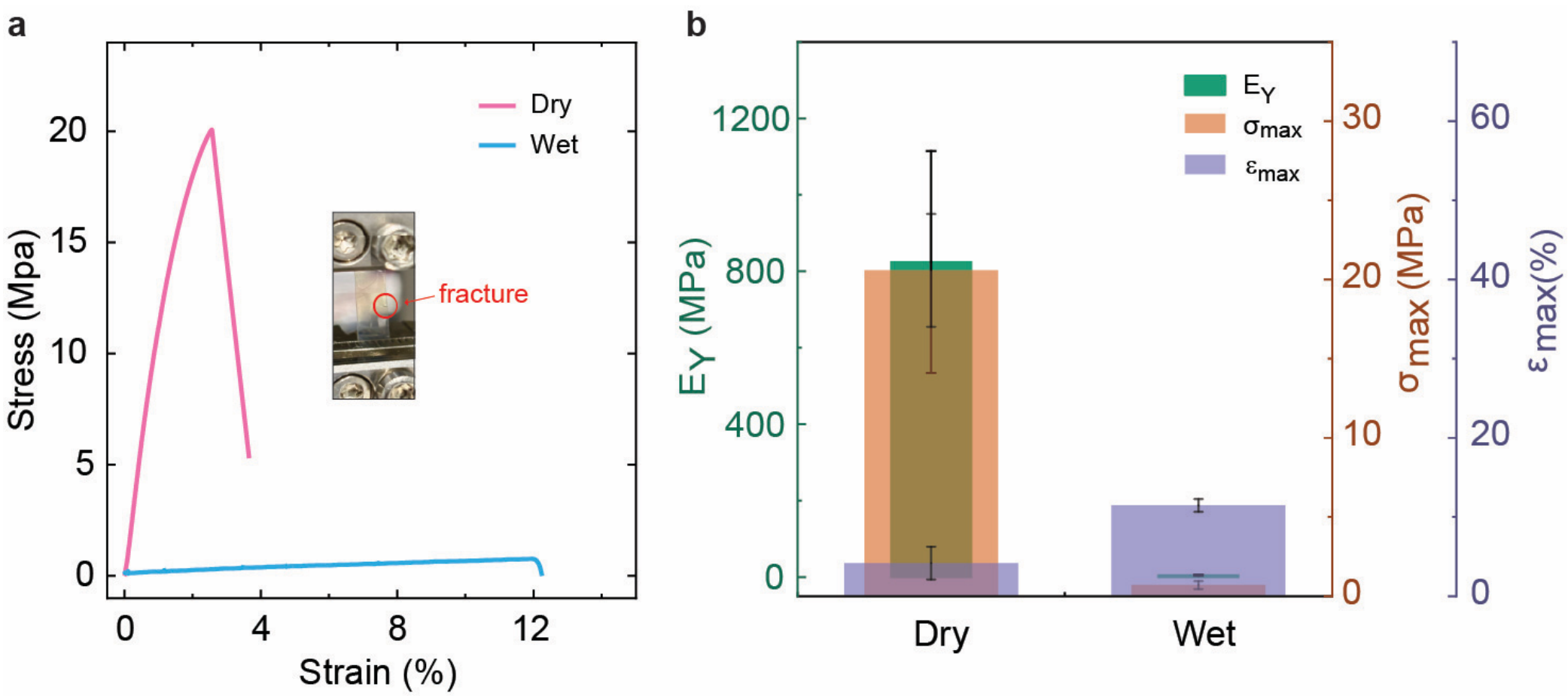

Supporting Figure 2 (for review purposes only). (a) Tensile stress–strain curves in the dry and wet state of $LCN_{AA+DMA+I}$ film after fracture. (b) The mechanical properties (Young's modulus, tensile strength, and fracture strain) of the corresponding films. Error bars represent the standard deviation obtained from three measurements. Film thickness: 100 µm.

4. The kinetics of the transparent-to-white transition are not quantitatively discussed. Based on the supplementary video, the transition appears relatively slow. The authors should provide quantitative measurements of the switching speed and discuss the factors governing the transition dynamics.

Our answer: We thank the reviewer for this insightful comment. To quantitatively investigate the kinetics of the transparent-to-white transition, we performed two sets of experiments:

1. **Thickness-dependent kinetics at room temperature**: $LCN_{AA+DMA+I}$ samples with thicknesses ranging from 5 to 100 µm were prepared, and the transition kinetics were monitored via transmittance changes (Supplementary Figs. 8 and 9). Thicker samples exhibited faster whitening than thinner samples, potentially due to more rapid formation of scattering domains within the bulk, which accelerates the formation of phase-segregated domains responsible for light scattering.

2. **Temperature-dependent measurements at fixed thickness**: For samples with constant thickness (20 µm), transition kinetics were monitored at different water temperature. The switching speed increased with increasing temperature (Supplementary Fig. 10&11), which is consistent with thermally activated nanoscale rearrangements and the dynamics of HB and XB interactions, which govern the phase separation process.

These results show that the transition kinetics are strongly dependent on both geometric (thickness) and environmental (solvent temperature) factors. The corresponding data have been added to the revised manuscript as follows (p. 10):

The kinetics of the transparent-to-white transition was monitored via transmittance measurements. The transition speed increased with both sample thickness and solvent temperature (Supplementary Figs. 8–11). The former is possibly due to more rapid formation of scattering domains within the bulk, and the latter can be rationalized through faster nanoscale rearrangements and the dynamics of HB and XB interactions, which govern the phase separation process.

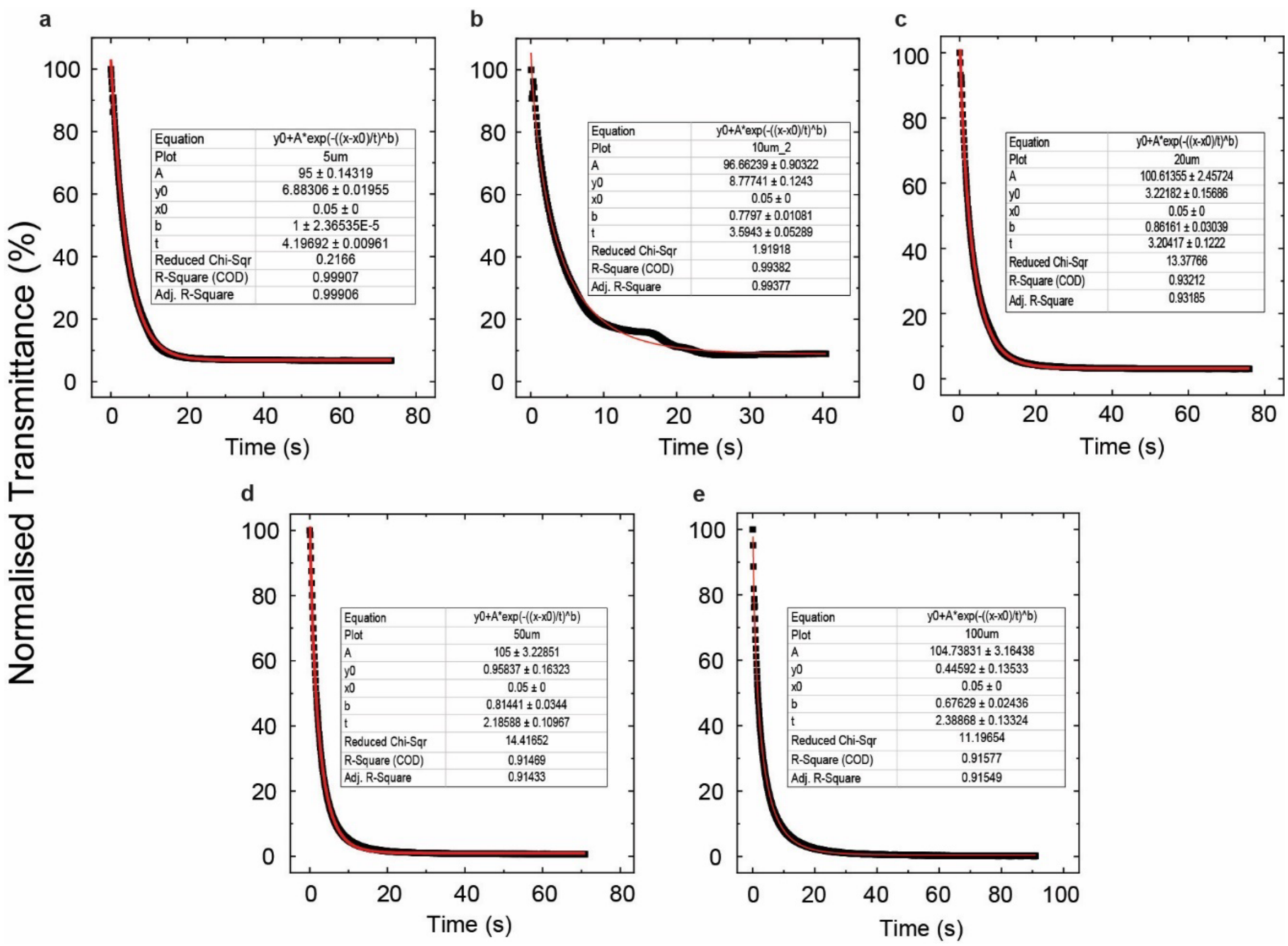

Supplementary Figure 8. Kinetics of transparent-to-white transition for $LCN_{AA+DMA+I}$ films of different thicknesses: (a) 5 µm, (b) 10 µm, (c) 20 µm, (d) 50 µm, and (e) 100 µm, via monitoring transmittance changes at 400nm at 25 °C.

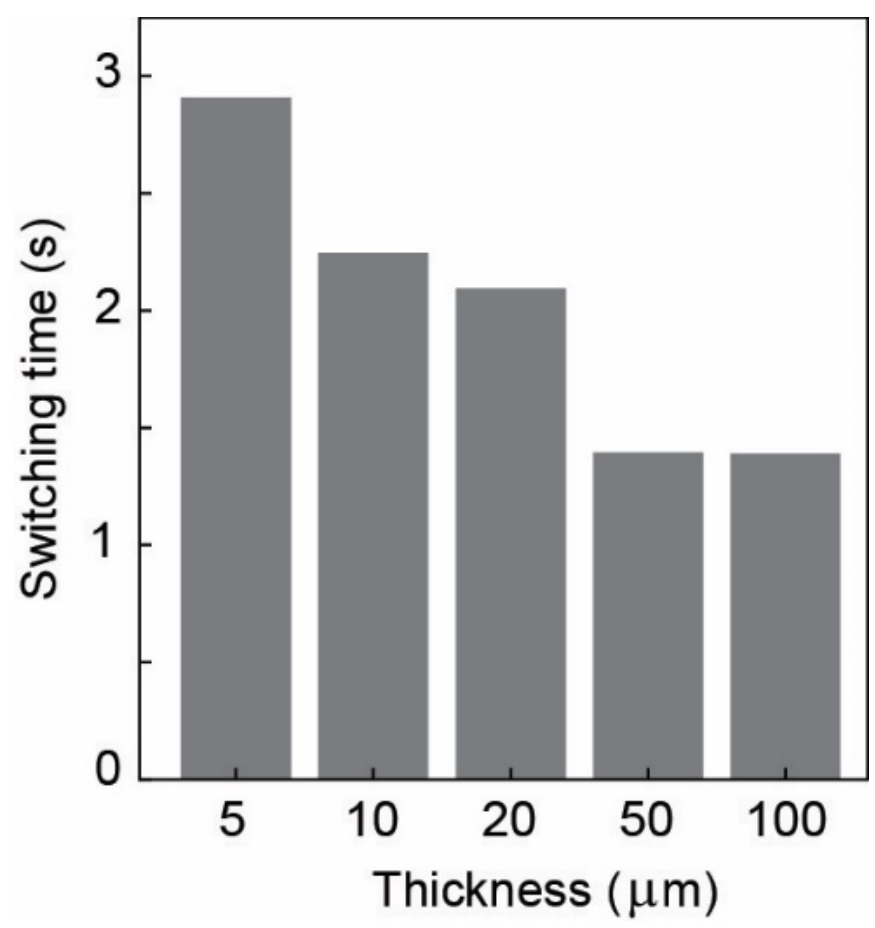

Supplementary Figure 9. Switching time in terms of half-lives for $LCN_{AA+DMA+I}$ films of different thickness, extracted from the data above.

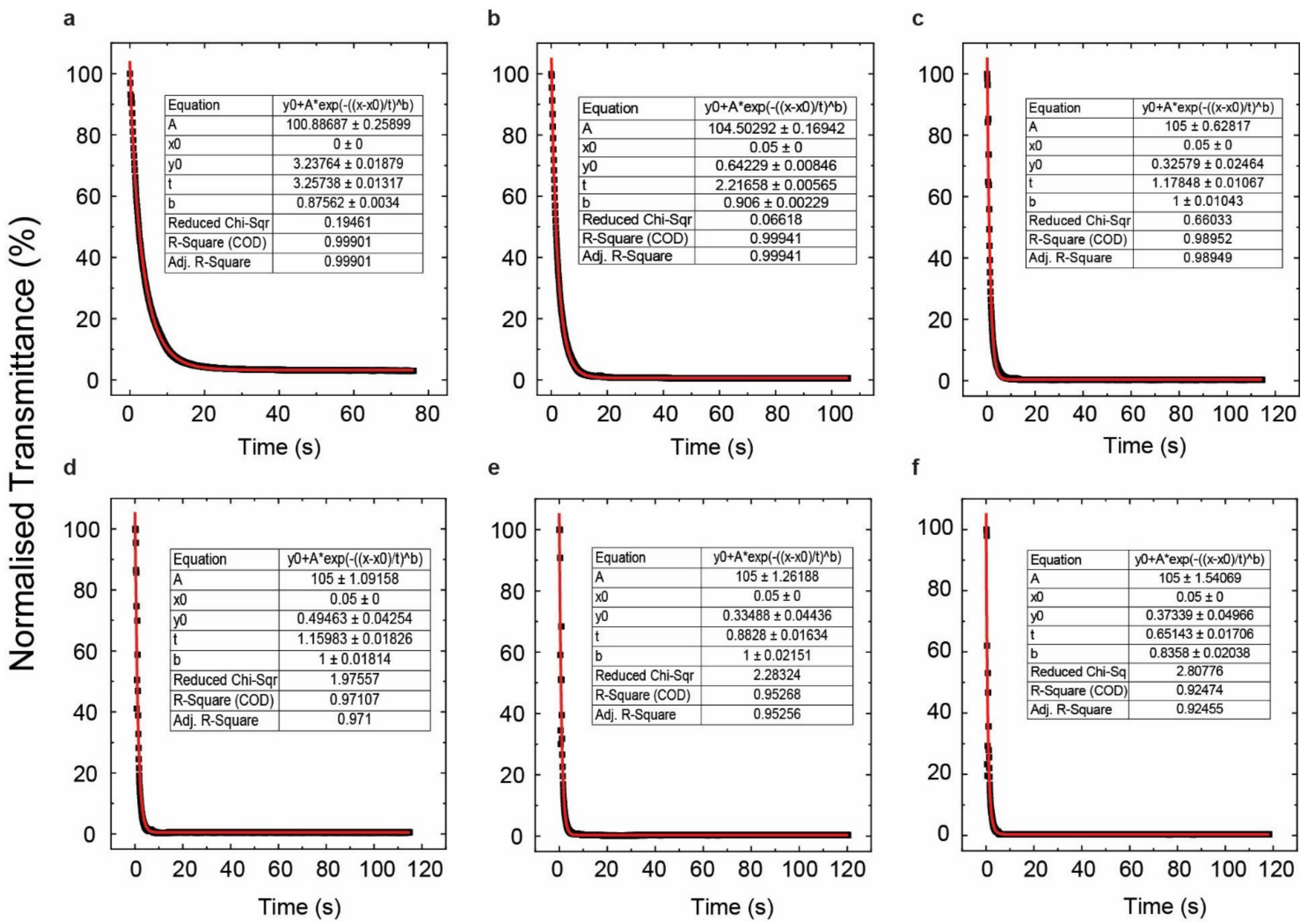

Supplementary Figure 10. Kinetics of transparent-to-white transition for 20 µm $LCN_{AA+DMA+I}$ film at different temperatures: (a) 25 ºC, (b) 30 ºC, (c) 40 ºC, (d) 60 ºC, (e) 80 ºC, and (f) 85 ºC, via monitoring transmittance changes at 400nm.

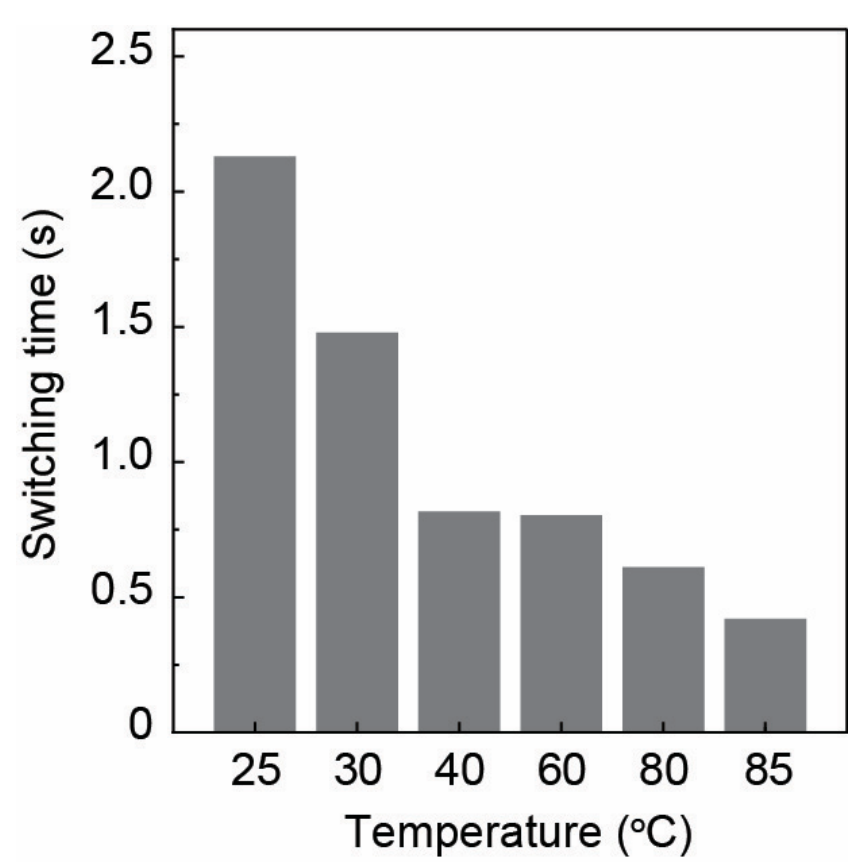

Supplementary Figure 11. Switching time in terms of half-lives for the 20 µm $LCN_{AA+DMA+I}$ film at different temperatures, extracted from the data above.

5. Although the authors demonstrate several proof-of-concept examples (e.g., tactile visualization, underwater recognition, and chemical sensing), the broader practical impact of the reversible whitening phenomenon remains unclear. Given the high-impact scope of the journal, the authors are encouraged to further clarify or demonstrate applications where this reversible optical transition offers clear advantages over existing approaches.

Our answer: The reversible whitening behavior of our LCN offers several advantages over existing approaches. Current dynamic scattering materials, such as hydrogels, typically rely on macroscopic structural changes or require external stimuli such as heating to induce optical transitions, which limits their tunability, response speed, and scalability. In contrast, our system undergoes reversible whitening through solvent-triggered disruption of supramolecular interactions: exposure to polar protic solvents breaks hydrogen bonds and drives nanoscale phase separation, producing scattering domains without significant bulk swelling or mechanical deformation. This mechanism enables rapid, robust, and fatigue-resistant switching, with scattering efficiencies exceeding 90% in 100 µm thick films. Moreover, the optical contrast and switching kinetics can be tuned through molecular design, incorporation of halogen-bonding dopants, and post-treatment with acid or base.

As proof-of-concept, we demonstrate potential applications in solvent discrimination, underwater visibility enhancement, biometric pattern recognition, and detection of poisonous methanol in methanol-ethanol mixture, none of which can be obtained with existing whitening materials relying on heating or other stimuli-induced transitions. Our approach relies solely on water or solvent exposure, which simplifies implementation and broadens practical applicability. These results establish a versatile, tunable platform for dynamic scattering materials with clear advantages in responsiveness, scalability, and energy efficiency.

The discussion in the revised manuscript is modified as follows (p 17): The above-mentioned applications cannot be obtained with existing whitening materials relying on heating or other stimuli-induced transitions. Our approach relies solely on water or solvent exposure, which simplifies implementation and broadens practical applicability.

Reviewer #3:

The authors introduce a liquid crystal network that reversibly switches to a highly scattering white state via solvent-induced disruption of supramolecular interactions. Polar protic solvents break hydrogen bonds, causing nanoscale phase segregation that efficiently scatters visible light without significant swelling or deformation. The response is rapid, robust, and fatigue-resistant, reaching 85% scattering efficiency in relatively thin films.
While the work is technically very good, I am a bit concerned about its novelty in context. It would be great if the author could better contextualize it, as several works of reversible witness have now been reported, for example: https://www.nature.com/articles/s43246-025-00956-3, https://advanced.onlinelibrary.wiley.com/doi/full/10.1002/adom.202302487
Saying that, I like the idea very much.

Our answer: We thank the reviewer for thoughtful comments and for highlighting related works. We fully acknowledge that several reversible whitening or scattering systems have been reported, including the references suggested. These prior works typically rely on stimuli-induced structural changes, which can limit their tunability, response speed, and mechanical integrity.

In contrast, our work introduces a liquid crystal network that undergoes reversible whitening through solvent-triggered disruption of supramolecular interactions, without significant macroscopic swelling or mechanical deformation. The transition is rapid, robust, and fatigue-resistant, with scattering efficiencies exceeding 90% in 100 μm films. The response is robust and can be used over at least 30 repeated wetting–drying cycles with no fatigue. Furthermore, by tailoring molecular design, incorporating XB dopants, and post-treatment with acid or base, we can tune both the optical contrast and switching kinetics, providing a level of dynamic control not reported in previous systems.

Finally, our study demonstrates potential applications in solvent discrimination, underwater visibility enhancement, and biometric pattern recognition, establishing a generalizable, tunable materials platform for dynamic scattering not possible with previously reported dynamic whitening systems. for these reasons, we believe our work is conceptually novel and of general interest to researchers working with dynamic scattering, or dynamic soft materials in general.

Discussion on these points has been added to the revised manuscript as follows (p. 18):

The mechanical robustness of these LCN films markedly exceeds that of previously reported hydrogels exhibiting switchable whiteness, [44] with a breaking stress of *ca.* 27 MPa for 100 μm thick LCN films *vs. ca.* 4.2 MPa for 1000 μm thick hydrogels. Remarkably, even films as thin as 10 μm exhibit up to 43% visible-light scattering in the wet state while maintaining excellent reversibility and showing no signs of mechanical fatigue over repeated wetting–drying cycles (Supplementary Fig. 16). The reflectance contrast reaches 40 % at 500 nm (Fig. 3f), and can be further increased to nearly 50% through simple post-treatment with acid or base. Unlike hydrogel-based dynamic scattering materials that often rely on macroscopic deformation or thermal activation, our LCN provides rapid, robust, and fatigue-resistant optical switching via solvent-triggered nanoscale phase segregation. Furthermore, the scattering

efficiency and response kinetics can be systematically tuned through molecular design, incorporation of XB dopants, and post-synthetic modification.

The observed whitening behavior is not limited to the specific mesogenic components employed here. RM82 and RM105 were selected as representative examples providing network stability and flexibility. When replacing RM82 with common mesogenic crosslinkers RM257 or C6BAPE, the dynamic whitening response is preserved (Supplementary Fig. 22). In fact, even non-liquid-crystalline building blocks (Networks 1-4, Supplementary Table 6) can be used, although in some cases at the expense of the mechanical properties of the network. These results indicate that the whitening transition is governed primarily by solvent-induced disruption of the hydrogen-bonded network, leading to nanoscale phase segregation and light scattering, rather than by the specific chemical identity of the mesogens, highlighting the generality of the underlying mechanism and establishing a broadly applicable design principle for dynamic scattering materials.

What is the volumetric change that is observed, as the author criticizes the hydrogel-based method in the intro? I think they should comment on this point.

Our answer: The weight change (gain) we observe in our film upon immersing in water is around 40% (Figure 4e). In comparison, the reported weight gain (swelling) in PNIPAm-based hydrogels exhibiting switchable whitening is ca 120% (https://doi.org/10.1039/d2ra08052d). Notably here %wt gain is calculated as $(W_{swollen}-W_{dry})/W_{dry}$ x 100%

As per reviewer's suggestion we measured volume change in our $LCN_{AA+DMA+I}$ film. The corresponding volumetric expansion upon water intake is around 150%, while corresponding literature values in thermoresponsive hydrogels are around 400-500% (shrinkage, not expansion like in our case) (https://doi.org/10.3390/gels9120926, https://doi.org/10.1002/adfm.202000754). Now here, %V change is calculated as V_{max}/V_{min} x 100%. Hence, although not absent, the volumetric changes in the LCN-based system are notably lesser than in switchable hydrogels.

While the authors claim that "The response is rapid, reversible, and requires no external stimulus, enabling simple visualization of touch patterns and suggesting potential for transient biometric marking and touch-responsive interfaces." It would be good to have a more critical discussion on its use. Similarly, I like the spring; it is a very nice concept, but I am not sure which concept can be used. I think more than guessing would be useful for quantifying the value of polarity vs. whiteness.

Our answer: We thank the reviewer for the comment. While $LCN_{AA+DMA+I}$ film shows rapid and reversible whitening, practical considerations include the stability of small-molecule dopants under repeated solvent exposure, the role and optimization of liquid crystal units, simplification, and scalability of sample preparation, and long-term performance under repeated use. We have added a discussion of these points in the revised manuscript to provide a critical assessment of both the advantages and limitations of the system.

About the spring concept. In our work, it serves as a conceptual framework linking local solvent polarity to network deformation and the resulting optical contrast. Beyond providing qualitative understanding, the spring also demonstrates the feasibility of fabricating films with complex and stable shapes, which is not possible with hydrogels with similar properties.

As an example, we show that conventional structures immersed in water can be difficult to locate, whereas those employing our responsive LCN network can be rapidly identified, highlighting potential applications in underwater detection. This concept also illustrates the possibility of designing water-triggered shape-morphing structures: by using the reversible HB interactions, water can act as a stimulus to drive shape-memory behavior, such as in shape-memory scaffolds or stents, analogous to our previous work with thermally triggered LCNs (https://doi.org/10.1038/s41467-022-34962-7) but now using water as the trigger. Therefore, the spring is intended to showcase potential applications and guide future designs, rather than serve as a final application. We have clarified this discussion in the revised manuscript.

To provide a quantitative assessment of the relationship between solvent polarity and optical contrast, we performed experiments using mixtures of methanol and ethanol at different ratios. This allowed us to systematically vary solvent polarity and measure the corresponding transmittance of the $LCN_{AA+DMA+I}$ films. The results, shown in Fig. 5d, demonstrate a clear correlation between methanol content and the degree of whitening, moving beyond qualitative observation. Representative schematic is also provided to visualize the effect.

Also, it would be nice to have the system's free path value. The author has already performed measurements on samples of different thicknesses.

Our answer: We thank the reviewer for the suggestion, but believe that such characterization is out of scope of the present work. In a separate study, we are investigating into the utility of dynamic scattering in the context of random lasers. Therein, detailed understanding of the scattering properties and their dynamic control are of utmost relevance.

Re: Switchable Whiteness in Liquid Crystal Networks via Solvent-Induced Microphase Segregation (manuscript NCOMMS-25-105727A), by R. Nasare, et al.

Point by Point Response to Reviewer Comments

Reviewer 1: *The authors have sufficiently addressed comments from my prior review, and I am happy to recommend acceptance for publication.*

Our answer: We thank the reviewer for the positive response and for recommending the manuscript for publication.

Reviewer 2*: The authors have addressed the comments with additional experiments except the following ones:*

Our answer: Thank you for the careful evaluation of our revised manuscript and for acknowledging our efforts in addressing the previous comments through additional experiments. We appreciate the reviewer's continued feedback and have carefully considered the remaining concerns. Detailed responses to each point are provided below.

1. Since mechanical durability is critical for practical applications, the authors are suggested to measure and compare the fracture toughness of the material in both states. No fracture toughness was measured.

Our answer: We apologise for not adding the fracture energy values in the previous revision. As far as we understand, the reviewer is interested in the fracture toughness of the material in both dry and wet states after creating an artificial crack. In the revised manuscript, we address this point by introducing an artificial notch corresponding to 10 % of the sample width (cf. Fig. 2d in https://doi.org/10.1002/adma.73541). We then measured the stress–strain curves of the sample in both transparent (dry) and scattering (wet) states and compared the fracture stress and strain values with those of original unnotched sample (Fig. 2f and Supplementary Fig. 6). In addition, we have now determined the fracture energy in both states and incorporated these results and corresponding discussion into the revised manuscript. We have also expanded the Methods section to describe the procedure used for calculating the fracture energy. The following modifications have been made to the manuscript:

Page 9:
We further evaluated the fracture toughness of $LCN_{AA+DMA+I}$ by introducing a 0.5 mm notch into the 5 mm wide LCN film and subsequently performing tensile testing (Supplementary Fig. 7). The presence of the notch reduced the breaking stress and strain in the dry state from 27 MPa to 21 MPa and from 28% to 4%, respectively. The corresponding fracture energy was calculated to be 36 kJ m^{-2}. In the wet state, the breaking stress decreased from 4 MPa to 0.7 MPa and the breaking strain from 31% to 11%.

The corresponding fracture energy was 3.42 kJ m^{-2}. The reduction in mechanical performance can be attributed to the notch acting as a stress concentrator, promoting crack propagation and lowering material's resistance to deformation and fracture. Nevertheless, the notched film retained the reversible whitening behavior and remained functional under repeated use.

Page 22:

Fracture energy calculations: The fracture energy was calculated according to $G_c = 6wc/\sqrt{\lambda_c}$, where c is the crack length (0.5 mm), and λ_c is the elongation at break of the notched sample. The strain energy w was obtained by integrating the stress–strain curve of the original unnotched sample up to the fracture strain ε_c, where $\varepsilon_c = \lambda_c$.

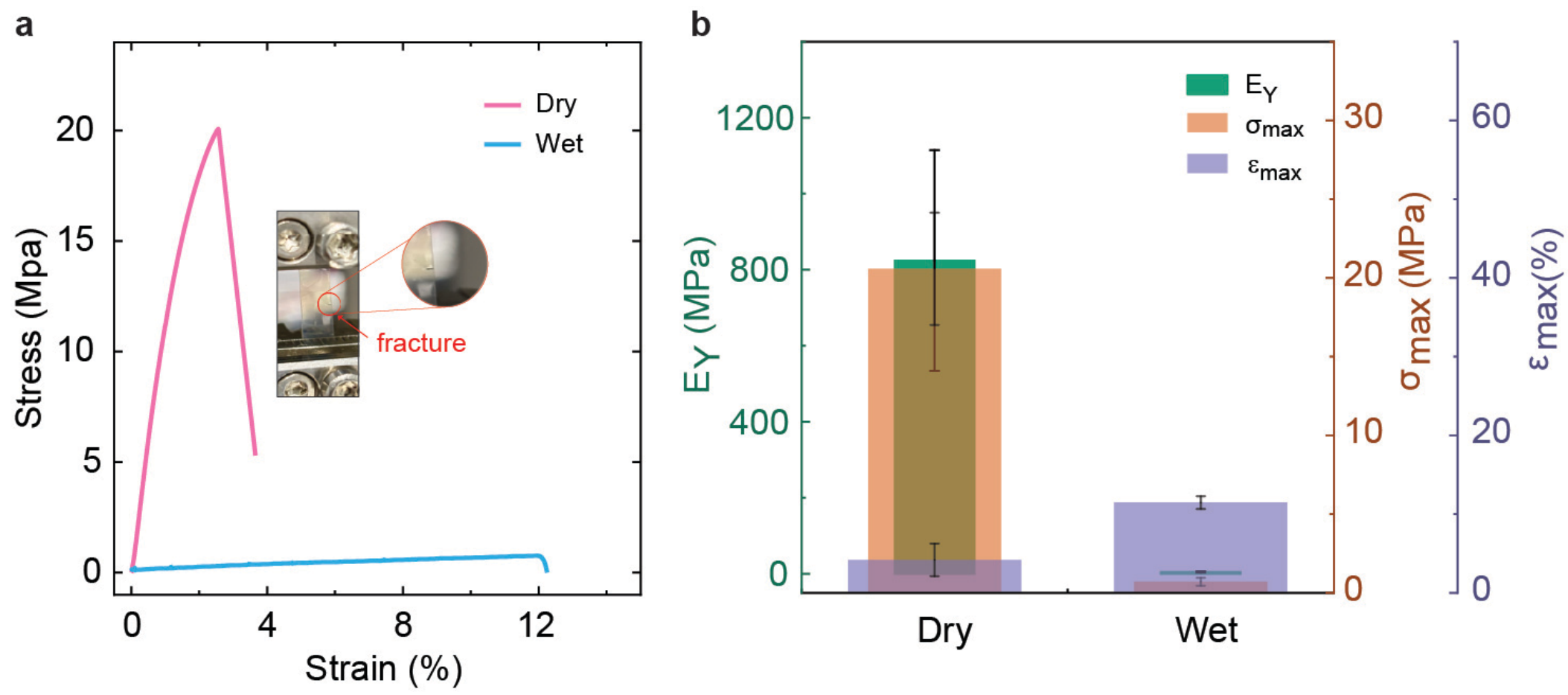

Supplementary Figure 7. (a) Tensile stress–strain curves of fractured $LCN_{AA+DMA+I}$ film in the dry and wet states. Sample width: 5 mm; Notch width 0.5 mm. (b) Corresponding values for Young's modulus, tensile strength, and fracture strain. Error bars represent the standard deviation obtained from three independent measurements. Film thickness: 100 µm.

2. additional demonstrations where this reversible optical transition offers clear advantages over existing approaches.

Our answer: To further demonstrate the advantages of the humidity-induced reversible optical transition, we have added two new application examples to the revised manuscript.

First, we demonstrate humidity-responsive optical modulation in a controlled humidity chamber, where the transparency of the material can be reversibly tuned by varying the humidity level. Second, we fabricated laser-patterned structures that remain optically transparent under dry conditions but become clearly visible when immersed in water, enabling water-triggered information revelation.

These demonstrations complement the applications already presented in the manuscript, incuding fingerprint visualization, underwater optical enhancement, and solvent discrimination. These examples highlight several advantages of our system over conventional dynamic light-scattering materials that lean on heating (hydrogels) or electric control (PDLCs), enabled by solvent-triggered disruption of supramolecular interactions and nanoscale phase separation. As a result, the optical transition occurs under mild conditions, while maintaining high optical contrast, rapid reversibility, and excellent tunability through molecular design and post-treatment strategies.

Together, the results presented establish the broad potential applicability of our platform for dynamic optical scattering materials in, *e.g.*, sensing and security.

The following modifications have been made to the manuscript:

Page 17:
In addition, we demonstrate relative humidity (RH) -dependent optical modulation in a controlled humidity chamber, where film transparency gradually decreases upon increasing RH (>60% RH) due to progressive water-vapor uptake and the resulting nanoscale phase segregation. This enables simple, instrument-free humidity sensing based on optical contrast changes (Supplementary Fig. 22). Furthermore, laser- patterned structures were fabricated to further exploit the spatially controlled scattering transition. The patterned films remain optically transparent under dry conditions but reveal hidden information upon water exposure, allowing on-demand visual decoding without external illumination or processing (Supplementary Fig. 23).

Page 18:
Overall, these results highlight that the same phase-segregation-driven scattering mechanism can be readily extended to environmental, chemical, and spatially encoded optical functions. In contrast to many existing whitening materials that rely on heating, bulk swelling, or electrical stimulus, our approach is triggered simply by exposure to water or polar solvents, enabling straightforward implementation and broadened applicability. The simplicity of stimulus input, combined with rapid optical readout and facile patterning capability, provides a versatile platform for low-energy, visually intuitive sensing and information encoding applications.

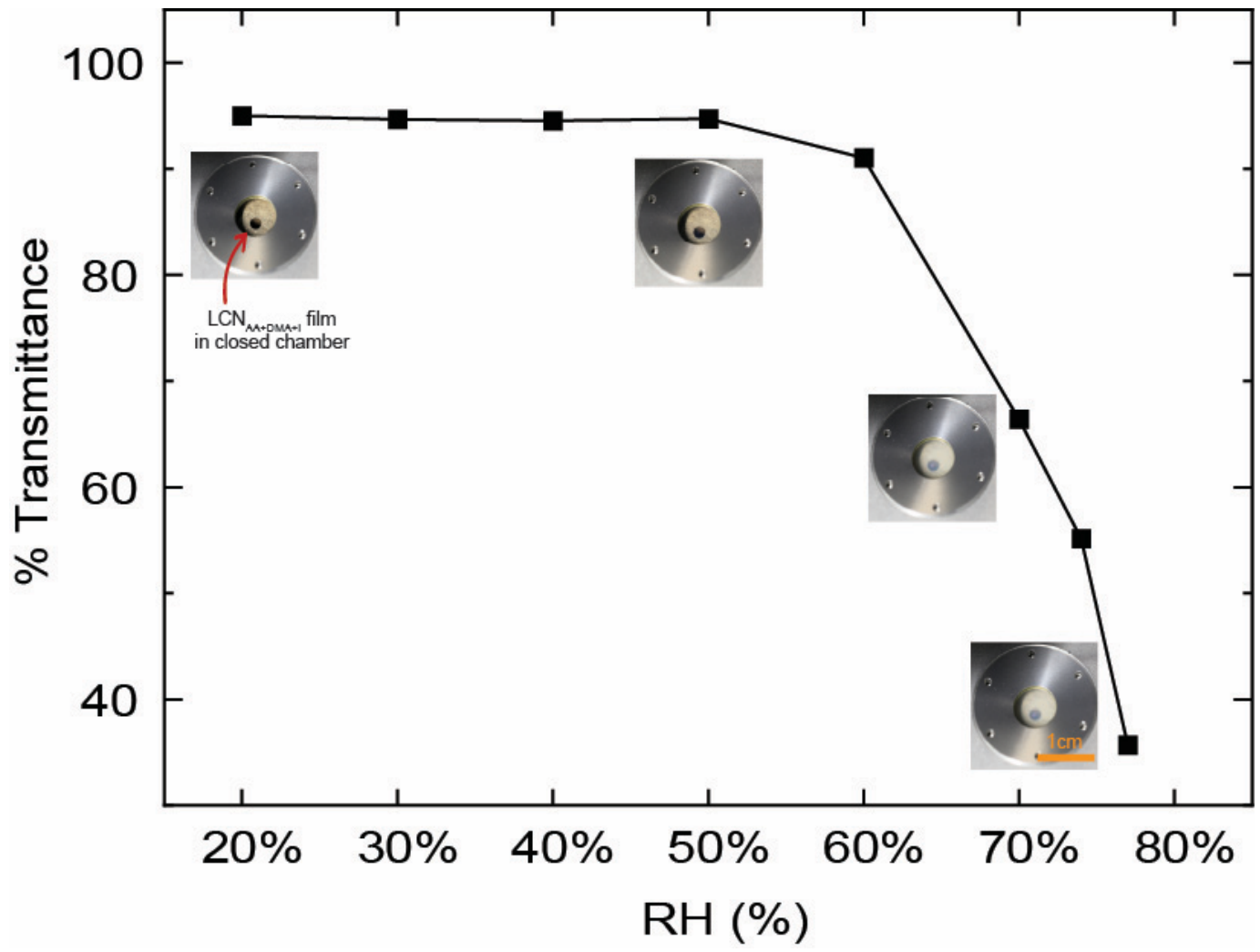

Supplementary Figure 22. Gradual decrease in the transmittance of $LCN_{AA+DMA+I}$ film at 450 nm with increasing relative humidity (RH) in a closed chamber at 22 °C. Insets show photographs of the films at the corresponding RH values. Film thickness: 100 µm.

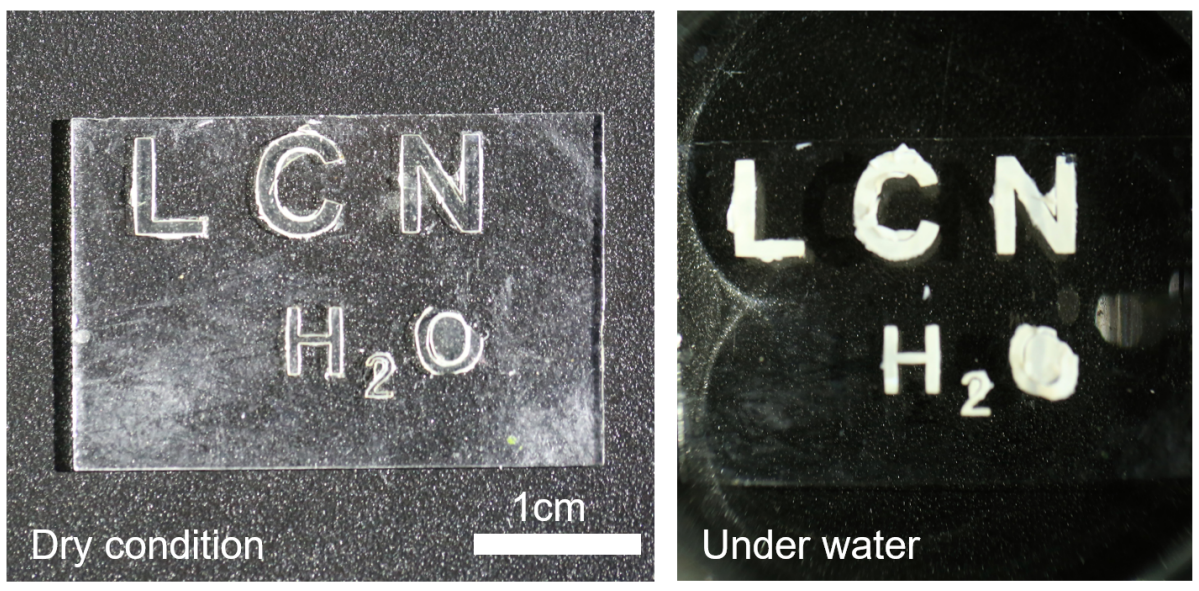

Supplementary Figure 23. Water-triggered revelation of encoded information in an $LCN_{AA+DMA+I}$ film. The laser-patterned film remains nearly transparent under dry conditions (left) but reveals the encoded "LCN H_2O" message upon immersion in water (right) due to the solvent-induced scattering transition. Film thickness: 100 µm. Scale bar: 1 cm.

Reviewer 3: *I appreciate the response of the author and almost all my comments have been addressed, I woudl however, like to insist on reporting the values of scattering mean free path measured from the sample at different thicknesses. I think it is not out of the scope, as it is pertinent to a more rigorous description of the scattering properties of the material produced. Plotting the Transmission vs thickness and showing that the scales are properly scaled also demonstrates that the samples with different thicknesses have the same morphology.*

Our answer: We thank the reviewer for the positive evaluation of our responses and apologize for not-this analysis in the previous revision.

In response to the reviewer's suggestion, we have now measured the total transmittance of $LCN_{AA+DMA+I}$ films with different thicknesses and analyzed their scattering behavior (Supplementary Fig. 9). Following the method reported by Chen *et al*. (https://pubs.acs.org/doi/10.1021/acsami.9b11816), we determined the characteristic attenuation length of the wet samples. Briefly, natural logarithm of the total transmittance (T_{total}) was plotted as a function of film thickness and fitted using linear regression. From the slope of the linear fit, a characteristic attenuation length (at 800 nm) of 116 µm was obtained. The corresponding results and discussion have now been incorporated into the revised manuscript, together with a description of the measurement and analysis procedure.

Page 10:

Additionally, the characteristic attenuation length of wet $LCN_{AA+DMA+I}$ was determined by measuring the total transmittance (T_{total}) of samples with different thicknesses and plotting $\ln(T_{total})$ as a function of sample thickness (Supplementary Fig. 9). The data exhibited a linear dependence, indicating that optical attenuation scales consistently with thickness and that the scattering morphology across samples with different thicknesses is comparable. From the slope of the linear fit, the characteristic attention length was calculated to be 116 µm at 800 nm.

Page 22:

Characteristic attenuation length: *The characteristic attenuation length l_s was determined using the Beer–Lambert law for scattering attenuation, i.e., $T_{total} = e^{-L/l_s}$, where T_{total} is the total transmittance and L is the sample thickness. Accordingly, $\ln(T_{total})$ was plotted as a function of sample thickness for wet $LCN_{AA+DMA+I}$ films. The characteristic attenuation length was obtained from the inverse of the slope for linear fit (0.00857 µm^{-1}), yielding a value of 116 µm.*

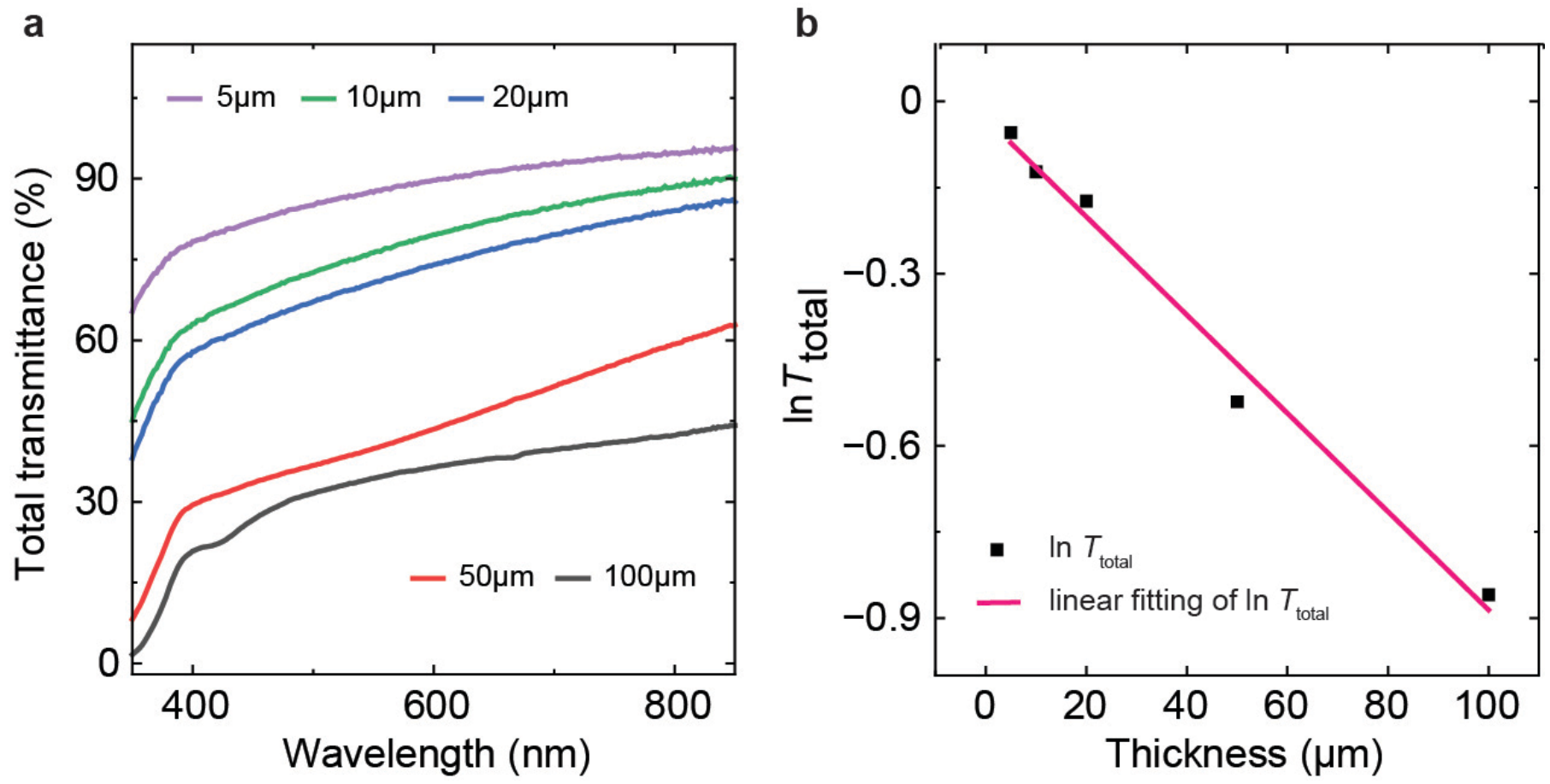

Supplementary Figure 9. (a) Total transmittance spectra of $LCN_{AA+DMA+I}$ film in wet state of different thicknesses. (b) Fitting between the ln (T_{total}) and the film thickness at 800 nm wavelength.

We thank the reviewer for the valuable suggestion. As requested, we have now explicitly reported the experimentally determined characteristic attenuation length in the manuscript (highlighted in yellow, page 7) and expanded the corresponding discussion.

The characteristic attenuation length and transport mean free path both characterize the effective propagation of light in scattering media and therefore provide quantitative measures of scattering strength. However, determination of the transport mean free path generally requires dedicated modelling together with assumptions regarding the scattering regime. Different approaches have been adopted in the literature. For example, transport mean free paths have been extracted by fitting diffusion-theory models to transmission measurements (https://advanced.onlinelibrary.wiley.com/doi/full/10.1002/adma.201704050), by applying Photonic Ohm's law (https://pubs.acs.org/doi/full/10.1021/acsami.6b15986), or by combining time-resolved optical measurements with diffusion theory (https://www.nature.com/articles/srep06075). Furthermore, for some highly scattering white materials, conventional diffusion theory has been reported to fail in describing the measured transmission behaviour, motivating the use of anomalous diffusion models instead (https://advanced.onlinelibrary.wiley.com/doi/full/10.1002/adma.201704050).

Because the present work is based on steady-state thickness-dependent total-transmittance measurements, we do not believe that extracting a transport mean free path using one particular model would be sufficiently rigorous or model-independent. Instead, we report the experimentally determined characteristic attenuation length, obtained directly from the exponential dependence of total transmittance on sample thickness. This quantity provides a robust experimental measure of the attenuation of transmitted light in our material without introducing additional modelling assumptions.

We have now calculated the corresponding attenuation coefficient (86.2 cm^{-1} at 800 nm) and compared it with attenuation coefficients reported for other optically scattering materials, including transparent wood and polymer aerogels (Refs. 53 and 54). Although these values were obtained for different material systems and analysis methods, they provide a useful benchmark demonstrating the strong optical attenuation achieved in the present material.

Accordingly, the discussion has been expanded in the main text (page 7), the experimental procedure has been described in the Methods section (page 16), and the relevant literature discussing attenuation coefficients has been added:

53. Merillas, B., Martín-de Leon, J., Villafane, F. & Rodriguez-Perez, M. A. Optical Properties of Polyisocyanurate–Polyurethane Aerogels: Study of the Scattering Mechanisms. Nanomaterials **12**, 1522 (2022).

54. Chen, H. et al. Thickness Dependence of Optical Transmittance of Transparent Wood: Chemical Modification Effects. ACS Appl. Mater. Interfaces **11**, 35451–35457 (2019).

Text added to the manuscript (page 7):

Additionally, the characteristic attenuation length of wet LCNAA+DMA+I was determined by measuring the total transmittance (Ttotal) of samples with different thicknesses and plotting ln(Ttotal) as a function of sample thickness (Supplementary Fig. 9). The data exhibited a linear dependence, indicating that optical attenuation scales consistently with thickness and that the scattering morphology across samples with different thicknesses is comparable. From the slope of the linear fit, the characteristic attenuation length (ls) was calculated to be 116 µm, corresponding to an attenuation coefficient (1/ls) of 86.2 cm-1 at 800 nm. This value is substantially larger than attenuation coefficients reported for transparent wood (ca. 1.5 cm-1) and polymer aerogels (ca. 4 cm-1),53,54 indicating strong attenuation of transmitted light in the present material.

Text added to the Experimental (page 16):

Characteristic attenuation length. The characteristic attenuation length, ls, was determined by fitting the thickness dependence of the total transmission to an exponential attenuation relation, Ttotal = e −L/ls, where Ttotal is the measured total transmittance and L is the sample thickness. Accordingly, ln(Ttotal) was plotted as a function of sample thickness for wet LCNAA+DMA+I films. The linear fit yielded a slope of 0.00857 µm-1, corresponding to a characteristic attenuation length of 116 µm.

Reviewer #2's Remarks to the Author, Version 0:

The authors report a liquid crystal network (LCN) that exhibits reversible whitening induced by solvent-triggered disruption of supramolecular interactions. Exposure to polar protic solvents disrupts hydrogen bonding and leads to nanoscale phase segregation, forming domains that scatter visible light without inducing significant macroscopic swelling or mechanical deformation. While the underlying mechanism is described and several brief demonstrations are provided, the broader significance and practical impact of this reversible whitening behavior remain insufficiently justified. In particular, the reviewer has the following questions.

1. The rationale for selecting RM82 and RM105 as the mesogenic building blocks is not sufficiently explained. It would be helpful if the authors could clarify why these specific mesogens were chosen and whether the proposed reversible whitening mechanism is generalizable to other commonly used mesogens (e.g., RM257).
2. In Figure 2h, repeatability is demonstrated over five dry–wet cycles. A larger number of cycles (e.g., 30–50 cycles) would be necessary to convincingly establish the robustness and long-term stability of the reversible whitening behavior.
3. Mechanical testing shows a reduction in modulus and breaking stress in the white state compared to the transparent state. Since mechanical durability is critical for practical applications, the authors are suggested to measure and compare the fracture toughness of the material in both states.
4. The kinetics of the transparent-to-white transition are not quantitatively discussed. Based on the supplementary video, the transition appears relatively slow. The authors should provide quantitative measurements of the switching speed and discuss the factors governing the transition dynamics.
5. Although the authors demonstrate several proof-of-concept examples (e.g., tactile visualization, underwater recognition, and chemical sensing), the broader practical impact of the reversible whitening phenomenon remains unclear. Given the high-impact scope of the journal, the authors are encouraged to further clarify or demonstrate applications where this reversible optical transition offers clear advantages over existing approaches.
